# Phenotyping patients with ischaemic heart disease at risk of developing heart failure: an analysis of the HOMAGE trial

**DOI:** 10.1002/ehf2.14465

**Published:** 2023-11-08

**Authors:** Diogo Santos‐Ferreira, Sílvia O. Diaz, João Pedro Ferreira, Nicolas Girerd, Pierpaolo Pellicori, Beatrice Mariottoni, Franco Cosmi, Mark Hazebroek, Job A.J. Verdonschot, Joe Cuthbert, Johannes Petutschnigg, Stephane Heymans, Jan A. Staessen, Burkert Pieske, Frank Edelmann, Andrew L. Clark, Patrick Rossignol, Ricardo Fontes‐Carvalho, John G.F. Cleland, Faiez Zannad

**Affiliations:** ^1^ Department of Cardiology Centro Hospitalar Vila Nova de Gaia/Espinho Vila Nova de Gaia Portugal; ^2^ Department of Surgery and Physiology, Cardiovascular R&D Centre ‐ UnIC@RISE Faculty of Medicine of the University of Porto Porto Portugal; ^3^ Inserm, Centre d'Investigation Clinique Plurithématique 1433, CHRU de Nancy, F‐CRIN INI‐CRCT Université de Lorraine Nancy France; ^4^ School of Cardiovascular and Metabolic Health University of Glasgow Glasgow UK; ^5^ Department of Cardiology Cortona Hospital Arezzo Italy; ^6^ Department of Cardiology Maastricht University Medical Center Maastricht The Netherlands; ^7^ Department of Cardiology University of Hull, Castle Hill Hospital Cottingham UK; ^8^ Department of Internal Medicine and Cardiology Charité University Medicine, Berlin Institute of Health (BIH), and German Centre for Cardiovascular Research (DZHK), Partner Site Berlin Berlin Germany; ^9^ Non‐Profit Research Association Alliance for the Promotion of Preventive Medicine (APPREMED) Mechelen Belgium; ^10^ German Heart Center Berlin Berlin Germany

**Keywords:** Coronary artery disease, Heart failure, Myocardial infarction, Proteomics, Spironolactone

## Abstract

**Aims:**

We aim to characterize the clinical and proteomic profiles of patients at risk of developing heart failure (HF), with and without coronary artery disease (CAD) or prior myocardial infarction (MI).

**Methods and results:**

HOMAGE evaluated the effect of spironolactone on plasma and serum markers of fibrosis over 9 months of follow‐up in participants with (or at risk of having) CAD, and raised natriuretic peptides. In this *post hoc* analysis, patients were classified as (i) neither CAD nor MI; (ii) CAD; or (iii) MI. Proteomic between‐group differences were evaluated through logistic regression and narrowed using backward stepwise selection and bootstrapping. Among the 527 participants, 28% had neither CAD or MI, 31% had CAD, and 41% had prior MI. Compared with people with neither CAD nor MI, those with CAD had higher baseline plasma concentrations of matrix metalloproteinase‐7 (MMP‐7), galectin‐4 (GAL4), plasminogen activator inhibitor 1 (PAI‐1), and lower plasma peptidoglycan recognition protein 1 (PGLYRP1), whilst those with a history of MI had higher plasma MMP‐7, neurotrophin‐3 (NT3), pulmonary surfactant‐associated protein D (PSPD), and lower plasma tumour necrosis factor‐related activation‐induced cytokine (TRANCE). Proteomic signatures were similar for patients with CAD or prior MI. Treatment with spironolactone was associated with an increase of MMP7, NT3, and PGLYRP1 at 9 months.

**Conclusions:**

In patients at risk of developing HF, those with CAD or MI had a different proteomic profile regarding inflammatory, immunological, and collagen catabolic processes.

## Introduction

Many patients with heart failure (HF) have coronary artery disease (CAD), which may lead to myocardial dysfunction that may or may not be preceded by a clinically overt myocardial infarction (MI).^1^, ^2^, ^3^ Clinically overt HF is often preceded by a prodrome characterized by sub‐clinical myocardial dysfunction and congestion, leading to atrial dilation and a rise in natriuretic peptides.^4^ Differences in pathophysiological pathways leading from cardiac dysfunction to HF might be reflected in the proteome. Patients with HF exhibit unique proteomic profiles, reflecting differences between ischaemic‐ and non‐ischaemic pathophysiological pathways,^5^, ^6^ and further helping risk stratification^7^, ^8^ and the development of new therapeutic targets.^9^ HF disease‐modifying drugs exert pleiotropic effects, including an associated change in proteomics that additionally helps further our understanding of the mechanisms behind the prognostic benefits of treatment.^10^, ^11^, ^12^, ^13^

In this *post hoc* analysis of the Heart ‘OMics’ in AGEing (HOMAGE) trial, we investigated differences in the clinical characteristics, plasma proteins, and collagen metabolism among participants classified by the presence or absence of CAD and MI.

## Materials and methods

### Trial design, population, and randomization

The design of the HOMAGE trial has been previously described.^14^, ^15^ Briefly, HOMAGE was a prospective, randomized, open‐label, blinded‐endpoint (PROBE) multi‐centre trial that evaluated the effects of spironolactone on markers of fibrosis and cardiac structure and function in people at risk of developing HF (ClinicalTrials.gov Identifier: NCT02556450).^15^ The trial was approved by relevant ethics committees and regulators. All participants provided written informed consent to participate in the trial.

HOMAGE included people aged 60 years or older with established CAD or at least two risk factors for CAD, namely, type 2 diabetes mellitus, hypertension, microalbuminuria, or abnormal electrocardiogram, who had an increased plasma concentration of natriuretic peptides (N‐terminal pro‐B type natriuretic peptide [NT‐proBNP] between 125 and 1000 ng/L or B‐type natriuretic peptide [BNP] between 35 and 280 ng/L).^14^, ^15^ Patients with known HF, an estimated glomerular filtration rate < 30 mL/min/1.73 m^2^, serum potassium > 5.0 mmol/L, left ventricular (LV) ejection fraction < 45%, atrial fibrillation, HF diagnosis, prescribed loop diuretics, or cardiovascular events in the prior 3 months were excluded.^15^

Patients were randomly allocated to either spironolactone or control in addition to their background medical therapy. Spironolactone was initiated at 25 mg/day and titrated to 50 mg/day, if tolerated, on top of usual care.

After randomization, patient visits were scheduled at 1 and 9 months, for clinical assessment, collection of blood samples, electrocardiogram, and echocardiographic examination.

### Clinical classification of participants

In this analysis, patients were classified as having (i) ‘no CAD/no MI’—with neither documented CAD nor prior MI, but having multiple risk factors for CAD; (ii) ‘CAD’—with known CAD but without prior MI; (iii) ‘MI’—prior MI, based on clinical history, supported by evidence of disease or procedures in medical records.

### Proteomic biomarkers

Plasma samples collected at baseline, month 1 and month 9 (or last) visits were analysed by the TATAA‐biocenter using the Olink® Proseek Multiplex cardiovascular II, cardiovascular III, and inflammation panels, resulting in a total of 276 proteins, as previously described.^10^, ^14^, ^15^ The abbreviations, respective full names, and Olink multiplex panels used are described in *Table* *S1*. Olink uses a proximity extension assay technology with dual‐recognition DNA‐coupled readout, where 92 oligonucleotide‐labelled antibody probe pairs are allowed to bind to their respective targets in the sample.^10^ All proteomic assays were blinded to randomization and clinical data. Protein expressions are log_2_‐transformed and provide relative quantification (NPX).^10^

### Laboratory assays

Serum procollagen type III N‐terminal peptide (PIIINP) and carboxy‐terminal telopeptide (CITP) were measured by radioimmunoassay (Orion DiagnosticaVR), procollagen type I carboxy‐terminal propeptide (PICP) by enzyme immunoassay (METRA; Quidel CorporationVR) and galectin‐3 by enzyme‐linked immunosorbent assay (ELISA) (BG MedicineVR). Plasma NT‐proBNP, high‐sensitivity troponin T (hsTnT) and growth differentiation factor 15 (GDF‐15) were measured by electro‐chemi‐luminescence (ELECSYSVR 2010 analyser: Roche Diagnostics, Mannheim, Germany). All laboratory assays were done blinded to randomization or clinical data and intra‐assay variations were < 10%.^14^

### Statistical analysis

Categorical variables are expressed as counts and percentages and continuous variables as median and interquartile range. Missing values were imputed with median of non‐missing values for continuous variables and mode for categorical variables. The absolute frequencies and relative percentages of missing values are listed in *Tables* *S2* and *S3*.

Baseline characteristics were compared between participants with neither CAD nor MI, CAD or MI, using Pearson's *χ* ^2^ test or Kruskal–Wallis rank sum test for categorical and continuous data, respectively. Statistical significance was assumed if *P* < 0.05.

Using logistic regression models, protein expression at baseline was compared between (i) ‘no CAD/no MI’ and ‘CAD’; (ii) ‘no CAD/no MI’ and ‘MI’; and (iii) ‘CAD’ and ‘MI’. Each protein was adjusted by the following clinically relevant variables: sex, age, waist circumference, diabetes, heart rate, systolic blood pressure, estimated glomerular filtration rate, blood potassium, haemoglobin, and urea.

The panel of differently expressed proteins (DEPs) was then narrowed through backward stepwise selection, using a *P*‐value < 0.05 and lower Akaike information criteria for protein retention. This showed the subset of proteins (‘proteomic profile’) associated with the presence of CAD and MI. Statistical correlation of the resulting proteomic profile was assessed through Pearson correlation. A bootstrap resampling with replacement (5000 bootstraps) was then applied to rank the most relevant proteins within each profile. For each bootstrap, we fitted a logistic regression model of the selected protein adjusted by clinically relevant variables, recovered raw *P*‐values, and corrected for multiple testing using a false discovery rate of 5%. Proteins associated with CAD or MI in at least 80% of the bootstrapped models were retained for mechanistic exploration.^16^

The effect of treatment with spironolactone on the change of all differentially expressed proteins was evaluated through an analysis of covariance. A linear regression model was fitted with each protein change (calculated as the protein expression at baseline subtracted from protein expression after 1 or 9 months of treatment) as the dependent variable, and treatment (spironolactone or usual care) plus the protein level at baseline as independent variables.

The influence of CAD and MI on the effect of spironolactone on the trial outcomes (systolic blood pressure, PICP, CITP, PIIINP, NT‐proBNP, LV mass, and left atrial volume) was investigated through an analysis of covariance model. A linear regression model was fitted with each endpoint change (calculated by subtracting each measurement after 1 and 9 months of treatment minus its baseline value) as the dependent variable, and its baseline value plus an interaction term between treatment and the presence of CAD or MI as independent variables.

Statistical analyses were performed using R statistical software, version 4.1.12.^17^, ^18^, ^19^, ^20^

## Results

### Baseline characterization of the population

A total of 527 participants was included, of whom 148 (28%) had no history of CAD or MI (‘no CAD/no MI’ group), 165 (31%) had CAD without MI (‘CAD’ group), and 214 (41%) had a previous MI (‘MI’ group). The population by groups is shown in *Table* *1*.

**Table 1:** Description of the population by group (‘no CAD/no MI’, ‘CAD’, ‘MI’)

Characteristic	No CAD/no MI (*N* = 148^1^)	CAD (*N* = 165^1^)	MI (*N* = 214^1^)	*P*‐value^2^
Men	86 (58%)	129 (78%)	177 (83%)	**<0.001**
Age (years)	77 (71, 81)	73 (69, 78)	71 (67, 76)	**<0.001**
Body mass index (kg/m^2^)	28.8 (25.7, 32.7)	28.1 (25.8, 30.6)	27.8 (25.2, 31.5)	0.3
Waist circumference (cm)	103 (97, 113)	102 (95, 110)	102 (94, 109)	0.088
Smoker (current or past)	82 (55%)	119 (72%)	148 (69%)	**0.004**
Arterial hypertension	146 (99%)	131 (79%)	136 (64%)	**<0.001**
Diabetes mellitus	116 (78%)	50 (30%)	51 (24%)	**<0.001**
Stroke/TIA	7 (4.7%)	10 (6.1%)	11 (5.1%)	0.9
COPD	6 (4.1%)	9 (5.5%)	18 (8.4%)	0.2
Standing heart rate (b.p.m.)	68 (62, 77)	64 (57, 72)	63 (57, 70)	**<0.001**
Standing SBP (mmHg)	142 (132, 157)	140 (127, 158)	133 (120, 152)	**<0.001**
Standing DBP (mmHg)	76 (70, 83)	78 (72, 87)	80 (72, 88)	**0.038**
Potassium (mmol/L)	4.2 (4.0, 4.4)	4.3 (4.2, 4.6)	4.3 (4.1, 4.6)	**<0.001**
Haemoglobin (g/dL)	13.5 (12.4, 14.4)	14.1 (13.3, 15.1)	14.3 (13.4, 15.1)	**<0.001**
Cholesterol (mg/dL)	158 (155, 193)	155 (135, 174)	155 (134, 166)	**<0.001**
Creatinine (μmol/L)	0.9 (0.8, 1.2)	1.0 (0.8, 1.1)	0.9 (0.8, 1.1)	0.7
Urea (mmol/L)	8.2 (6.2, 11.2)	6.7 (5.6, 9.7)	6.6 (5.5, 9.6)	**<0.001**
eGFR‐MDRD (mL/min/1.73 m^2^)	71 (55, 84)	72 (63, 81)	73 (63, 86)	0.2
Aspirin	68 (46%)	135 (82%)	170 (79%)	**<0.001**
Other antiplatelets	15 (10%)	33 (20%)	51 (24%)	**0.004**
Anticoagulant	8 (5.4%)	8 (4.8%)	13 (6.1%)	0.9
Beta‐blocker	65 (44%)	124 (75%)	176 (82%)	**<0.001**
Thiazides	45 (30%)	21 (13%)	19 (8.9%)	**<0.001**
ACEI or ARB	131 (89%)	109 (66%)	176 (82%)	**<0.001**
Statin/lipid‐lowering agent	80 (54%)	146 (88%)	205 (96%)	**<0.001**
CCB	38 (26%)	33 (20%)	37 (17%)	0.15
Randomized to spironolactone	75 (51%)	78 (47%)	112 (52%)	0.6
Galectin‐3 (μg/L)	17.5 (15.0, 21.4)	15.6 (13.2, 18.6)	15.5 (13.1, 19.3)	**<0.001**
GDF‐15 (ng/L)	1.939 (1.298, 2.664)	1.347 (1.061, 1.656)	1.400 (946, 1.812)	**<0.001**
NT‐proBNP (ng/L)	264 (176, 442)	240 (166, 362)	263 (146, 422)	0.4
MMP‐1 (μg/L)	10.9 (7.0, 17.7)	9.8 (6.5, 14.1)	10.0 (6.6, 16.1)	0.2
PIIINP (μg/L)	3.9 (3.2, 4.8)	3.9 (3.3, 5.0)	3.9 (3.0, 5.0)	0.5
PICP (μg/L)	81.4 (64.8, 98.6)	79.2 (66.9, 96.8)	80.6 (64.9, 95.5)	0.7
CITP (μg/L)	4.1 (3.0, 5.9)	3.8 (2.9, 4.8)	3.5 (2.7, 4.5)	**0.001**
QRS duration (ms)	90 (82, 118)	92 (85, 104)	92 (84, 102)	0.7
LVEDVI (mL/m^2^)	42 (35, 45)	42 (36, 48)	43 (38, 51)	**<0.001**
LVEF (%)	63 (62, 65)	63 (62, 66)	63 (58, 64)	**0.004**
LVMI (g/m^2^)	97 (88, 114)	94 (79, 108)	95 (82, 103)	**0.003**
Men	98 (89, 120)	95 (79, 108)	95 (84, 110)	**0.022**
Women	96 (86, 109)	90 (72, 104)	86 (79, 95)	**0.018**
LAVI (mL/m^2^)	30 (27, 35)	30 (27, 34)	30 (26, 35)	0.9
E (m/s)	0.7 (0.6, 0.8)	0.7 (0.6, 0.8)	0.7 (0.6, 0.8)	0.066
A (m/s)	0.8 (0.8, 1.0)	0.8 (0.7, 0.9)	0.8 (0.6, 0.9)	**<0.001**
E/A ratio	0.77 (0.63, 0.92)	0.84 (0.72, 1.03)	0.83 (0.68, 1.02)	**0.003**
E/e′ ratio	9.6 (8.4, 12.1)	9.3 (8.0, 10.8)	9.3 (7.5, 10.8)	**0.005**
TAPSE (mm)	22.1 (19.3, 26.9)	19.9 (16.2, 24.4)	22.1 (19.3, 25.9)	**<0.001**

Statistically significant *P*‐values are presented in bold.

^1^ *n*/*N* (%); Median (interquartile range).

^2^ Pearson's chi‐squared test; Kruskal–Wallis rank sum test.

A, late wave mitral valve flow velocity; ACEI, angiotensin‐converting enzyme inhibitor; ARB, angiotensin receptor blocker; CCB, calcium‐channel blockers; CITP, collagen type‐1 C‐terminal telopeptide; COPD, chronic obstructive pulmonary disease; DBP, diastolic blood pressure; E, early wave mitral valve flow velocity; e′, early diastolic tissue velocity; eGFR‐MDRD, estimated glomerular filtration rate, modification of diet in renal disease; GDF‐15, growth differentiation factor 15; LAVI, left atrial volume index; LVEDV, left end‐diastolic volume index; LVEF, left ventricular ejection fraction (biplane); LVMI, left ventricular mass index; MMP‐1, matrix metalloproteinase‐1; NT‐proBNP, N‐terminal pro‐brain natriuretic peptide; PICP, procollagen type‐I C‐terminal pro‐peptide; PIIINP, procollagen type‐III N‐terminal pro‐peptide; SBP, systolic blood pressure; TAPSE, tricuspid annular plane systolic excursion; TIA, transient ischaemic attack.

Compared with ‘no CAD/no MI’, CAD subjects were younger, more frequently men and current or past smokers, and less frequently hypertensive and diabetic, had higher haemoglobin levels and lower total cholesterol, had lower LV mass index, tricuspid annular plane systolic excursion and E/e’ (early diastolic tissue velocity) ratio, and higher E/A ratio, were more frequently medicated with anti‐platelet agents (including aspirin), beta‐blockers and lipid‐lowering agents, and less frequently medicated with angiotensin‐converting enzyme inhibitors (ACEI) or angiotensin receptor blockers (ARB), and thiazides.

Compared with ‘no CAD/no MI’, MI participants were younger, more frequently men and current or past smokers, and less likely to be hypertensive or diabetic, had lower total cholesterol and higher haemoglobin levels, had lower LV mass, a lower E/e′ and higher E/A ratios. Patients with previous MI were more often treated with aspirin and other antiplatelets, beta‐blockers, lipid‐lowering agents, and less frequently thiazides.

Compared with CAD group, participants with MI were younger, less frequently hypertensive, and more frequently medicated with ACEI or ARB, beta‐blockers, and lipid‐lowering drugs. Participants with prior MI also had higher TAPSE values.

### Association between proteomics and coronary disease

Proteins found differentially expressed between groups are listed in *Table* *2*, while the full list of proteins is shown in the *Tables* *S4* to *S9*.

**Table 2:** Proteins included in the logistic regression model, adjusted for the defined clinically relevant variables^#^.

CAD (vs. no CAD/no MI)				MI (vs. no CAD/no MI)			
Protein	OR (95% CI)	*z*	*P*	Protein	OR (95% CI)	*z*	*P*
MMP7^1^ ^,^ ^2^	4.37 (2.32, 8.85)	4.3	<0.001	MMP7^1^ ^,^ ^2^	3.27 (1.85, 6.01)	4.0	<0.001
GAL4^1^ ^,^ ^2^	3.06 (1.66, 5.83)	3.5	<0.001	NT3^1^ ^,^ ^2^	3.06 (1.64, 5.98)	3.4	<0.001
OSM	0.55 (0.37, 0.81)	−3.0	0.003	PSPD^1^ ^,^ ^2^	1.92 (1.31, 2.85)	3.3	0.001
PAI^1^ ^,^ ^2^	1.65 (1.19, 2.31)	2.9	0.003	CTSD	3.04 (1.54, 6.19)	3.1	0.002
TGF‐α	0.25 (0.10, 0.64)	−2.9	0.004	KIM1	1.82 (1.25, 2.70)	3.1	0.002
TRANCE	0.51 (0.31, 0.81)	−2.8	0.006	GRN	4.21 (1.67, 11.03)	3.0	0.003
PGLYRP^1^ ^,^ ^2^	0.42 (0.23, 0.77)	−2.8	0.006	OPG	4.03 (1.60, 10.57)	2.9	0.004
TPA	1.84 (1.20, 2.88)	2.7	0.006	TRANCE^1^ ^,^ ^2^	0.48 (0.29, 0.78)	−2.9	0.004
CD8A^1^	0.55 (0.35, 0.84)	−2.7	0.007	TPA	1.58 (1.09, 2.34)	2.4	0.018
GRN^1^	3.61 (1.44, 9.57)	2.7	0.008	ADA	2.21 (1.15, 4.35)	2.3	0.019
ADAMTS13	5.62 (1.57, 21.69)	2.6	0.010	UPA	2.00 (1.08, 3.58)	2.3	0.020
PCSK9	3.24 (1.35, 8.09)	2.6	0.010	GDF15	1.89 (1.08, 3.32)	2.2	0.026
TNFRSF9	0.45 (0.24, 0.82)	−2.5	0.012	ACE2	1.68 (1.07, 2.69)	2.2	0.027
CCL23	0.44 (0.22, 0.86)	−2.4	0.017	AGRP	1.88 (1.08, 3.33)	2.2	0.028
MCP1	2.69 (1.20, 6.32)	2.3	0.020	IL8	1.63 (1.06, 2.56)	2.2	0.028
GIG	1.37 (1.06, 1.79)	2.3	0.021	PCSK9	2.51 (1.10, 5.85)	2.2	0.030
IGFBP2	0.53 (0.30, 0.91)	−2.3	0.024	CCL16	1.61 (1.04, 2.50)	2.2	0.031
OPN	0.50 (0.27, 0.91)	−2.2	0.025	VWF	1.29 (1.02, 1.64)	2.1	0.036
CST5^1^	0.54 (0.30, 0.94)	−2.1	0.033	NTproBNP (log)^1^	1.59 (1.03, 2.49)	2.1	0.040
TLT2	0.51 (0.27, 0.94)	−2.1	0.034	IL5^1^	0.75 (0.57, 0.99)	−2.1	0.040
CASP3	1.24 (1.02, 1.53)	2.1	0.038	HSTNT (log)	1.90 (1.03, 3.58)	2.0	0.042
HSP27	1.95 (1.03, 3.79)	2.0	0.043	HAOX1	1.27 (1.01, 1.60)	2.0	0.042
PECAM1	1.47 (1.01, 2.15)	2.0	0.044	MMP12	1.58 (1.02, 2.49)	2.0	0.042
MMP9	0.37 (0.14, 0.97)	−2.0	0.048	BLMHYDROLASE	2.02 (1.04, 4.06)	2.0	0.043
MMP1^1^	0.76 (0.58, 1.00)	−2.0	0.050	GAL4	1.74 (1.02, 3.04)	2.0	0.045
				CXCL6	1.36 (1.01, 1.86)	2.0	0.046
MI *vs*. CAD				BNP	1.32 (1.01, 1.74)	2.0	0.047
Protein	OR (95% CI)	z	*p*	TNFRSF10A	2.37 (1.02, 5.67)	2.0	0.048
MMP9^1^	2.29 (1.26, 4.47)	2.60	0.010	IGFBP7	2.64 (1.03, 7.11)	2.0	0.049
NTproBNP (log)	1.45 (1.03, 2.06)	2.10	0.036				
PAI^1^	0.80 (0.64, 0.99)	−2.10	0.038				
MB	1.49 (1.03, 2.21)	2.10	0.040				
IL7	0.75 (0.57, 0.99)	−2.10	0.041				
CHI3L1^1^	1.28 (1.01, 1.63)	2.00	0.042				
KIM1	1.35 (1.00, 1.83)	2.00	0.049				

Only proteins with *P* < 0.05 are shown.

CI, confidence interval; OR, odds ratio.

^#^ Sex, age, waist circumference, diabetes, heart rate, systolic blood pressure, estimated glomerular filtration rate, blood potassium, haemoglobin, and urea).

^1^ Proteins selected by stepwise backward.

^2^ Proteins retained by bootstrapping (FDR < 0.05 in at least 80% of bootstrapped models). Proteins common to both phenotypes are highlighted.

Twenty‐five differentially expressed proteins (DEPs) at baseline were found between ‘no CAD/no MI’ and CAD. Of these, eight were retained in the ‘proteomic profile’ by stepwise selection, and further narrowed to four after bootstrapping. CAD participants had higher levels of matrilysin (MMP‐7), galectin‐4 (GAL4) and plasminogen activator inhibitor 1 (PAI), and lower levels of peptidoglycan recognition protein 1 (PGLYRP1). After adjusting for baseline medication (aspirin, other anti‐platelets, beta‐blockers, thiazides, ACEI/ARB, and lipid‐lowering agents) in the logistic regression, differences in MMP7, GAL4, and PAI remained statistically significant, but not PGLYRP1 (*Table* *S7*).

Twenty‐nine DEPs were found between ‘no CAD/no MI’ and MI before spironolactone treatment (or control strategy). Of these, six were retained in the ‘proteomic profile’, and further narrowed to four after bootstrapping. MI participants had higher levels of MMP‐7, neurotrophin‐3 (NT3) and pulmonary surfactant‐associated protein D (PSPD), and lower levels of tumour necrosis factor‐related activation‐induced cytokine (TRANCE). The differences in proteins were also statistically significant in a logistic regression model adjusted for baseline medication (*Table* *S8*).

Seven DEPs were found between CAD and MI groups before HOMAGE intervention. Of these, three were selected in the initial stepwise analysis, but none was retained from the bootstrapped models.

No significant statistical correlation was found between proteins included the proteomic profile (*Table* *S10*).

### Changes in proteomic profile with treatment

The effect of treatment with spironolactone on the main protein changes described above is summarized in *Table* *3*. Treatment with spironolactone increased the circulating levels of NT3, MMP‐7 and PGLYRP1 at month 9.

**Table 3:** Changes in protein concentrations after 1 and 9 months of treatment with spironolactone (vs. control)

		Month 1			Month 9		
Protein	Associated with	β (95% CI)	*z*	*P*	β (95% CI)	*z*	*P*
GAL4	CAD	0.03 (−0.04, 0.09)	0.7	0.48	0.04 (−0.03, 0.11)	1.1	0.27
PGLYRP1	CAD	0.07 (0.00, 0.14)	1.8	0.068	0.12 (0.04, 0.21)	3	**0.003**
PAI	CAD	0.05 (−0.09, 0.18)	0.6	0.53	0.09 (−0.05, 0.24)	1.3	0.20
NT3	MI	0.03 (−0.05, 0.11)	0.7	0.47	0.11 (0.02,0.19)	2.5	**0.012**
PSPD	MI	−0.05 (−0.14, 0.03)	−1.2	0.22	−0.08 (−0.17, 0.01)	−1.7	0.083
TRANCE	MI	−0.02 (−0.09, 0.06)	−0.5	0.65	−0.01 (−0.09, 0.07)	−0.2	0.84
MMP7	CAD & MI	0.01 (−0.06, 0.08)	0.3	0.78	0.10 (0.02, 0.17)	2.6	**0.009**

Results reported as β coefficient (95% CI), z‐score and *P*‐value for the treatment variable. Statistically significant *P*‐values are presented in bold.

GAL4, galectin‐4; MMP7, matrilysin; NT3, neurotrophin‐3; PAI, plasminogen activator inhibitor 1; PGLYRP1, peptidoglycan recognition protein 1; PSPD, pulmonary surfactant associated protein D; TRANCE, tumour necrosis factor‐related activation induced cytokine.

### Influence of coronary disease and HOMAGE results

The impact of atherosclerotic group on study endpoints is detailed in Table *4*. There was no influence of group on the effect of spironolactone on the change of systolic blood pressure, PICP, CITP, PIIINP, NT‐proBNP, LV mass, or left atrial volume (interaction *P* > 0.1 for all).

**Table 4:** Effect of treatment with spironolactone *vs.* control in patients classified by the presence of CAD and MI

		Month 1			Month 9		
	Group	β (95% CI)	*z*	*P*	β (95% CI)	*z*	*P*
SBP	No CAD/no MI	−3.74 (−8.31, 0.84)	−1.6	0.50	−6.83 (−11.60, −2.06)	−2.8	0.16
	CAD without MI	−6.46 (−10.80, −2.12)	−2.0		−12.83 (−17.35, −8.31)	−5.6	
	MI	−7.11 (−10.91, −3.30)	−3.7		−8.16 (−12.12, −4.19)	−4.0	
PICP	No CAD/no MI	−5.36 (−10.73, 0.02)	−2.0	0.83	−6.82 (−13.54, −0.09)	−2.0	0.94
	CAD without MI	−6.35 (−11.45, −1.26)	−2.5		−8.30 (−14.67, −1.94)	−2.6	
	MI	−4.25 (−8.72, 0.21)	−1.9		−7.07 (−12.65, −1.48)	−2.5	
CITP	No CAD/no MI	0.08 (−0.38, 0.54)	0.3	0.27	0.54 (−1.34, 0.27)	−1.3	0.22
	CAD without MI	0.20 (−0.24, 0.63)	0.9		0.30 (−0.46, 1.06)	0.8	
	MI	0.54 (0.16, 0.93)	2.8		0.30 (−0.37, 0.97)	0.9	
PIIINP	No CAD/no MI	−0.13 (−0.56, 0.30)	−0.6	0.89	−0.36 (−0.87, 0.15)	−1.4	0.23
	CAD without MI	−0.19 (−0.59, 0.21)	−0.9		0.23 (−0.25, 0.71)	0.9	
	MI	−0.06 (−0.41, 0.30)	−0.3		−0.19 (−0.61, 0.24)	−0.9	
NT‐proBNP	No CAD/no MI	−17.27 (−28.04, −6.50)	−3.2	0.080	−14.34 (−24.70, −3.98)	−2.7	0.23
	CAD without MI	−9.18 (−19.40, 1.03)	−1.8		−2.88 (−12.71, 6.94)	−0.6	
	MI	−1.37 (−10.34, 7.60)	−0.3		−4.37 (−13.00, 4.25)	−1.0	
LVM	No CAD/no MI	−3.35 (−6.66, −0.05)	−2.0	0.23	−0.14 (−3.57, 3.29)	−0.1	0.39
	CAD without MI	−0.10 (−3.23, 3.04)	−0.1		−3.38 (−6.63, −0.13)	−2.0	
	MI	0.17 (−2.58, 2.92)	0.1		−1.36 (−4.21, 1.50)	−0.9	
LAV	No CAD/no MI	−1.39 (2.86, 0.09)	−1.8	0.26	−1.7 (−3.26, −0.13)	−2.1	0.42
	CAD without MI	0.32 (−1.08, 1.71)	0.4		−2.3 (−3.78, −0.81)	−3.0	
	MI	−0.37 (−1.59, 0.86)	−0.6		−0.98 (−2.28, 0.32)	−1.5	

Results reported as β coefficients, *z*‐score, and *P*‐value for the interaction.

CITP, collagen type‐1 C‐terminal telopeptide; LAV, left atrial volume; LVM, left ventricular mass; NT‐proBNP, N‐terminal pro‐brain natriuretic peptide; PICP, procollagen type‐I C‐terminal pro‐peptide; PIIINP, procollagen type‐III N‐terminal pro‐peptide; SBP, systolic blood pressure.

## Discussion

**Figure 1:**
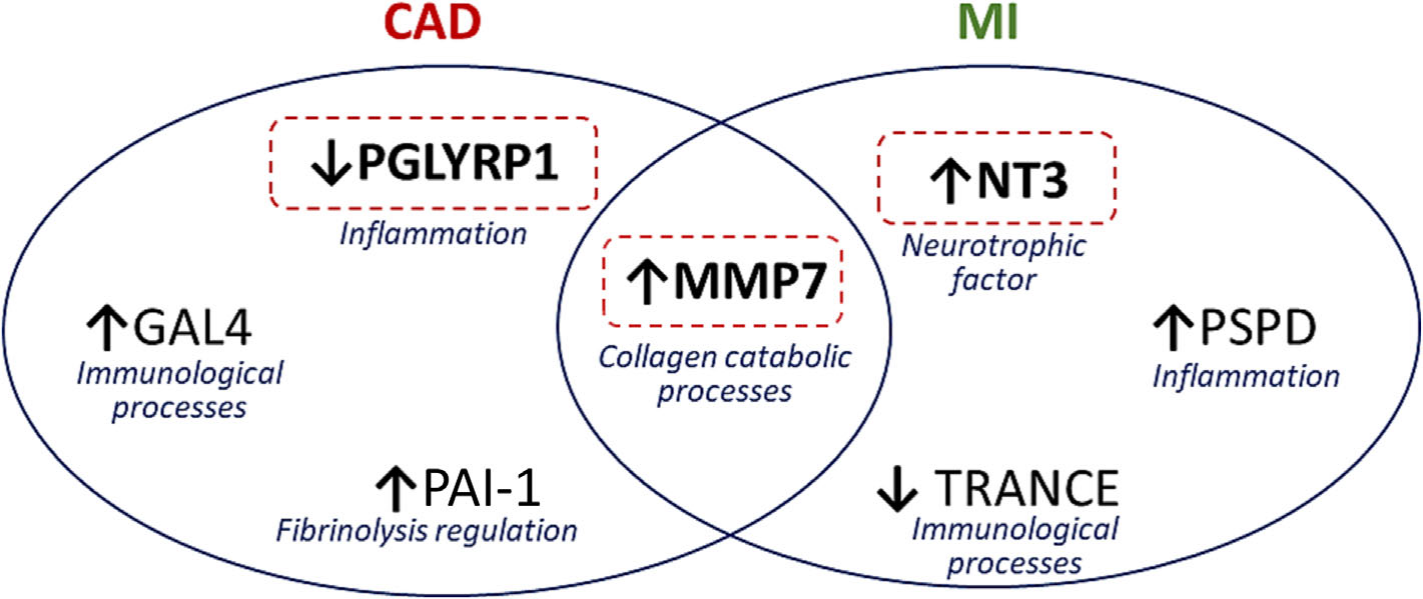
Differentially expressed proteins in CAD and MI participants, when compared with ‘no CAD/no MI’ group, and respective pathways involved. Arrows indicate higher or lower proteins levels. Dashed red boxes indicate proteins that were increased with spironolactone treatment. GAL4, galectin‐4; MMP7, matrilysin; NT3, neurotrophin‐3, PAI, plasminogen activator inhibitor 1; PGLYRP1, peptidoglycan recognition protein 1; PSPD, pulmonary surfactant‐associated protein D; TRANCE, tumour necrosis factor‐related activation‐induced cytokine.

This study shows that the plasma proteome of patients at risk of developing HF but without history of CAD or MI differs substantially from those with clinical CAD with or without previous known MI. Several mechanisms of disease may be implied in these differential processes towards HF, including differential activity of proteins involved in inflammatory and immunological pathways, as well as collagen catabolic processes (*Figure* *1*).

Proteomic differences may reflect distinct participants' characteristics. Those with CAD and MI were more likely to be men and to have a smoking history, but had a lower prevalence of hypertension and diabetes mellitus, when compared with those with no overt coronary disease. These differences may reflect the trial design, as part of the inclusion criteria was the presence of risk factors, such as hypertension and diabetes, in the absence of known CAD. Additionally, baseline medication was also different between groups, reflecting an underlying condition ‐ specifically, more antiplatelet, lipid‐lowering, and beta‐blocking agents in both CAD and MI groups, following the ischaemic cardiomyopathy's usual care,^21^, ^22^, ^23^ and higher use of thiazides among the more frequently hypertensive ‘no CAD/MI’ patients. Total serum cholesterol was lower in patients with CAD or MI, as they were more likely to be prescribed lipid‐lowering therapies.^24^

The echocardiographic data (*Table* *1*) show that the ‘no CAD/no MI’ group had a higher LV mass index and E/e′ ratio, and a lower E/A ratio, reflecting higher LV filling pressures, hypertrophy and diastolic dysfunction consistent with the higher prevalence of diabetes and hypertension in this group,^25^, ^26^, ^27^ and perhaps suggesting a different pathway towards HF in this subset.

Several DEPs were identified between groups at baseline, with clustering around collagen and inflammatory response processes. MMP‐7 was higher in those with coronary disease, whether or not they had a prior known MI, compared with the ‘no CAD/no MI’ group. MMP‐7 is high in both remote and infarct regions after MI.^28^, ^29^ The role of MMP‐7 in cardiovascular disease is controversial: although in animal models MMP‐7 gene knockout reduces atherosclerotic burden, mice with the knockout have a greater risk of sudden death and cardiac fibrosis. MMP‐7 activity after MI may thus prevent excessive fibrosis.^30^ However, other models suggest that the absence of MMP‐7 has positive effects on LV remodelling post‐MI, as well as increasing survival and myocardial conduction.^28^, ^31^ MMP‐7 also mediates cardiac adverse remodelling in uraemic mice,^32^ and higher MMP‐7 expression was associated with greater risk of death and HF hospitalization in patients with HF with preserved EF in the TOPCAT trial.^33^ GAL4, a lectin mostly expressed in the gastrointestinal tract and involved in immunoregulatory functions,^34^ was increased in CAD group. Plasma concentrations of this protein are elevated in patients with HF,^35^ especially if they have diabetes,^36^ and is associated with worse prognosis.^35^, ^37^ Higher plasma GAL4 in the general population is associated with all‐cause and cardiovascular mortality, coronary events and incident HF.^38^ PAI—a fibrinolysis regulator^39^—was also higher in patients with CAD. Higher PAI concentrations are associated with a higher risk for MI,^39^ as well as with adverse cardiac events in HF patients.^40^ Plasma PGLYRP1 was lower among patients with CAD without MI. Higher plasma concentrations of this immune system protein are associated with both acute and chronic CAD, as well as HF. Long‐term administration of recombinant PGLYRP1 leads to atherogenesis and LV systolic dysfunction in animal models.^41^ Also, in a healthy population study, higher PGLYRP1 was associated with an increased risk of atherosclerotic cardiovascular events.^42^

PSPD was overexpressed in participants with MI, versus ‘no CAD/no MI’. The impact of this inflammatory protein on cardiovascular disease is controversial—although most studies demonstrate that higher PSPD levels are associated with a greater risk of cardiovascular events (including HF hospitalization, sudden cardiac death, MI, and stroke),^43^ others have shown that it is not linked to the development of subclinical atherosclerosis.^44^ TRANCE was lower among those with prior MI. Lower concentration of this cytokine involved in immune response regulation, angiogenesis, and bone remodelling^45^ has also been associated with a greater risk of cardiovascular disease in patients with psoriasis.^46^

There were no DEPs after bootstrapping between CAD and MI groups. This suggests that the pattern of gene expression seems to be similar in all patients with clinical evidence of coronary disease, regardless of previous overt acute coronary ischaemic events.

Treatment with spironolactone changed the plasma concentrations of proteins associated with the presence of CAD regardless of prior MI. Spironolactone significantly increased serum MMP‐7, unravelling a potential anti‐fibrotic mechanism of the drug, as described above.^30^ NT3 is also increased by MRA use, which is a neurotrophic factor that has been shown to be reduced in experimental models of HF.^47^ Spironolactone increased circulating levels of PGLYRP1, a pro‐inflammatory protein with atherogenic properties, as described above.^42^ Further studies should follow regarding the impact of modulating these proteomic pathways and HF progression.

There was no significant interaction between treatment with spironolactone and the presence of CAD or MI on the HOMAGE trial endpoints. This suggests that the effect of spironolactone on systolic blood pressure, PICP, CITP, PIIINP, NT‐proBNP, LV mass, or left atrial volume is not influenced by underlying atherosclerotic/ischaemic presentation.

### Clinical perspectives

The majority of HF trials have focused on symptomatic phases of the disease (ACC/AHA stages C and D^48^), and there are few data on pre‐HF patients (ACC/AHA stage B^48^), in whom preventive strategies are more relevant. One promising study suggests that spironolactone may induce reversal of subclinical LV dysfunction.^49^

Further understanding of the proteomic pathways leading to HF in an asymptomatic phase helps clarify the underlying mechanisms involved, improving diagnosis and risk stratification, and may, ultimately, lead to new pharmacological approaches to limit disease progression. The present analysis identified seven DEPs in people with raised natriuretic peptides and a previous history of CAD or MI versus those with atherosclerotic risk factors but no documented CAD or MI. The effect of spironolactone was not affected by the presence of coronary disease, consistent with the view that current drug therapy for HF should not restricted by aetiology.^48^, ^50^

### Limitations

Being a *post hoc* analysis of a randomized trial, our findings are exploratory and hypothesis‐generating. Subgroup division was based only on the available clinical data. As there was no systematic investigation to exclude coronary disease and prior MI in all patients, some participants classified as ‘no CAD/no MI’ might, in fact, have had subclinical coronary atherosclerosis or have previously suffered from silent MI, possibly causing grouping misclassification. Additionally, covariate adjustment influenced directionally some of the associations, suggesting that these biomarker associations should be interpreted in light of patients' characteristics. No information was available regarding the exact time between MI and trial inclusion, although any history of vascular (including coronary) events in the previous 3 months was part of the exclusion criteria. In consequence, we cannot analyse temporal proteomic variation after coronary events. Moreover, some of the differences between groups might reflect interaction between cardiovascular risk factors and usual medication—although this was partially corrected in the adjusted logistic regression model, the potential effect of non‐included variables cannot be foreseen. Finally, cardiac amyloidosis is a common undiagnosed entity leading to HF in a more elderly population, and no systematic studies were carried out in HOMAGE to exclude the diagnosis formally. It is possible that some participants with subclinical cardiac amyloidosis might have been included and be unevenly distributed throughout group classification, potentially influencing the proteomic results. However, as we have excluded patients with NT‐proBNP >1000, it is very unlikely we have included anyone with clinically significant cardiac amyloidosis.

## Conclusions

This analysis found differentially expressed proteins associated with clinical CAD, with or without a history of MI, consistent with abnormalities in the immune system and collagen catabolism. Treatment with spironolactone increased plasma/serum concentrations of MMP7, NT3, and PGLYRP1. The presence of CAD or MI did not influence the effect of spironolactone in HOMAGE endpoints, namely systolic blood pressure, PICP, CITP, PIIINP, NT‐proBNP, LV mass, or left atrial volume.

## Funding

HOMAGE trial was funded by the European Union 7th Framework Programme for Research and Technological Development (grant: 305507 http://www.homage‐hf.eu).^14^, ^15^ SOD is financed by the European Regional Development Fund (ERDF), through the North Regional Operational Program in the framework of the project HEALTH‐UNORTE: Setting‐up biobanks and regenerative medicine strategies to boost research in cardiovascular, musculoskeletal, neurological, oncological, immunological and infectious diseases (reference NORTE‐01‐0145‐FEDER‐000039). JGFC is supported by a British Heart Foundation Centre of Research Excellence (grant number RE/18/6/34217).

## Author contributions

D.S.F. designed the first version of the manuscript and adapted it according to co‐authors' suggestions. S.O.D. performed the statistical analysis. J.P.F. supervised the analysis and construction of the manuscript. All co‐authors have read and approved the final version of the article.

## Conflict of interest

The authors declare that there are no conflicts of interest related to the present analysis.

## Supporting information


**Table S1.**Protein names and corresponding Olink panel, in alphabetical order.
**Table S2.** Absolute and relative frequencies of missing clinical, analytical and echocardiographic data.
**Table S3.** Absolute and relative frequencies of missing protein data. Full names of proteins can be found in *supplemental table 1*.
**Table S4.** Proteins included in the logistic regression model, adjusted to the defined clinically relevant variables, comparing ‘CAD’ *versus* ‘no CAD/no MI’ participants. Full names of proteins can be found in *supplemental table 1*.
**Table S5.** Proteins included in the logistic regression model, adjusted to the defined clinically relevant variables, comparing ‘MI’ *versus* ‘no CAD/no MI’ participants. Full names of proteins can be found in *supplemental table 1*.
**Table S6.** Proteins included in the logistic regression model, adjusted to the defined clinically relevant variables, comparing ‘MI’ *versus* ‘CAD’ participants. Full names of proteins can be found in *supplemental table 1*.
**Table S7.** Proteins included in the logistic regression model, adjusted to the defined clinically relevant variables and medication, comparing ‘CAD’ *versus* ‘no CAD/no MI’ participants. Full names of proteins can be found in *supplemental table 1*.
**Table S8.** Proteins included in the logistic regression model, adjusted to the defined clinically relevant variables and medication, comparing ‘MI’ *versus* ‘no CAD/no MI’ participants. Full names of proteins can be found in *supplemental table 1*.
**Table S9.** Proteins included in the logistic regression model, adjusted to the defined clinically relevant variables and medication, comparing ‘MI’ *versus* ‘CAD’ participants. Full names of proteins can be found in *supplemental table 1*.
**Table S10.** Pearsons' correlation coefficient between proteins included in the proteomic profile.
