# Supplementary material for: Phenotyping patients with ischaemic heart disease at risk of developing heart failure: an analysis of the HOMAGE trial
Source: ESC Heart Fail. 2023 Nov 8;11(1):209–18. doi: 10.1002/ehf2.14465 (PMC10804163; doi:10.1002/ehf2.14465)
Supplement: Supplementary file 1 — Table S1.Protein names and corresponding Olink panel, in alphabetical order. Table S2. Absolute and relative frequencies of missing clinical, analytical and echocardiographic data. Table S3. Absolute and relative frequencies of missing protein data. Full names of proteins can be found in supplemental table 1. Table S4. Proteins included in the logistic regression model, adjusted to the defined clinically relevant variables, comparing ‘CAD’ versus ‘no CAD/no MI’ participants. Full names of proteins can be found in supplemental table 1. Table S5. Proteins included in the logistic regression model, adjusted to the defined clinically relevant variables, comparing ‘MI’ versus ‘no CAD/no MI’ participants. Full names of proteins can be found in supplemental table 1. Table S6. Proteins included in the logistic regression model, adjusted to the defined clinically relevant variables, comparing ‘MI’ versus ‘CAD’ participants. Full names of proteins can be found in supplemental table 1. Table S7. Proteins included in the logistic regression model, adjusted to the defined clinically relevant variables and medication, comparing ‘CAD’ versus ‘no CAD/no MI’ participants. Full names of proteins can be found in supplemental table 1. Table S8. Proteins included in the logistic regression model, adjusted to the defined clinically relevant variables and medication, comparing ‘MI’ versus ‘no CAD/no MI’ participants. Full names of proteins can be found in supplemental table 1. Table S9. Proteins included in the logistic regression model, adjusted to the defined clinically relevant variables and medication, comparing ‘MI’ versus ‘CAD’ participants. Full names of proteins can be found in supplemental table 1. Table S10. Pearsons' correlation coefficient between proteins included in the proteomic profile. [file EHF2-11-209-s001.docx]

Supplemental table 1 – Protein names and corresponding Olink panel, in alphabetical order.

| **Protein** | **Protein full name** | **Olink panel** |
| --- | --- | --- |
| ACE2 | Angiotensin-converting enzyme 2 | Cardiovascular II |
| ADA | Adenosine deaminase | Inflammation |
| ADAMTS13 | A disintegrin and metalloproteinase with thrombospondin motifs 13 | Cardiovascular II |
| ADM | Pro-adrenomedullin | Cardiovascular II |
| AGRP | Agouti-related protein | Cardiovascular II |
| ALCAM | CD166 antigen | Cardiovascular III |
| AMBP | Protein AMBP | Cardiovascular II |
| ANG1 | Angiopoietin-1 | Cardiovascular II |
| APN | Aminopeptidase N | Cardiovascular III |
| ARTN | Artemin | Inflammation |
| AXIN1 | Axin-1 | Inflammation |
| AXL | Tyrosine-protein kinase receptor UFO | Cardiovascular III |
| AZU1 | Azurocidin | Cardiovascular III |
| BetaNGF | Beta-nerve growth factor | Inflammation |
| BLMHYDROLASE | Bleomycin hydrolase | Cardiovascular III |
| BMP6 | Bone morphogenetic protein 6 | Cardiovascular II |
| BOC | Brother of CDO | Cardiovascular II |
| BP14E | Eukaryotic translation initiation factor 4E-binding protein 1 | Inflammation |
| CA5A | Carbonic anhydrase 5A, mitochondrial | Cardiovascular II |
| CASP3 | Caspase-3 | Cardiovascular III |
| CASP8 | Caspase-8 | Inflammation |
| CCL11 | Eotaxin | Inflammation |
| CCL15 | C-C motif chemokine 15 | Cardiovascular III |
| CCL16 | C-C motif chemokine 16 | Cardiovascular III |
| CCL17 | C-C motif chemokine 17 | Cardiovascular II |
| CCL19 | C-C motif chemokine 19 | Inflammation |
| CCL20 | C-C motif chemokine 20 | Inflammation |
| CCL23 | C-C motif chemokine 23 | Inflammation |
| CCL24 | C-C motif chemokine 24 | Cardiovascular III |
| CCL25 | C-C motif chemokine 25 | Inflammation |
| CCL28 | C-C motif chemokine 28 | Inflammation |
| CCL3 | C-C motif chemokine 3 | Cardiovascular II, Inflammation |
| CCL4 | C-C motif chemokine 4 | Inflammation |
| CD163 | Scavenger receptor cysteine-rich type 1 protein M130 | Cardiovascular III |
| CD244 | Natural killer cell receptor 2B4 | Inflammation |
| CD4 | T-cell surface glycoprotein CD4 | Cardiovascular II |
| CD40 | Tumor necrosis factor receptor superfamily member 5 | Inflammation |
| CD40-L | CD40 ligand | Cardiovascular II |
| CD5 | T-cell surface glycoprotein CD5 | Inflammation |
| CD6 | T-cell differentiation antigen CD6 | Inflammation |
| CD84 | SLAM family member 5 | Cardiovascular II |
| CD8A | T-cell surface glycoprotein CD8 alpha chain | Inflammation |
| CD93 | Complement component C1q receptor | Cardiovascular III |
| CDCP1 | CUB domain-containing protein 1 | Inflammation |
| CDH5 | Cadherin-5 | Cardiovascular III |
| CEACAM8 | Carcinoembryonic antigen-related cell adhesion molecule 8 | Cardiovascular II |
| CHI3L1 | Chitinase-3-like protein 1 | Cardiovascular III |
| CHIT1 | Chitotriosidase-1 | Cardiovascular III |
| CNTN1 | Contactin-1 | Cardiovascular III |
| COL1A1 | Collagen alpha-1(I) chain | Cardiovascular III |
| CPA1 | Carboxypeptidase A1 | Cardiovascular III |
| CPB1 | Carboxypeptidase B | Cardiovascular III |
| CSF1 | Macrophage colony-stimulating factor 1 | Inflammation |
| CST5 | Cystatin-D | Inflammation |
| CSTB | Cystatin-B | Cardiovascular III |
| CTRC | Chymotrypsin-C | Cardiovascular II |
| CTSD | Cathepsin D | Cardiovascular III |
| CTSL1 | Cathepsin L1 | Cardiovascular II |
| CTSZ | Cathepsin Z | Cardiovascular III |
| CX3CL1 | Fractalkine | Inflammation |
| CXCL1 | Growth-regulated alpha protein | Cardiovascular II, Inflammation |
| CXCL10 | C-X-C motif chemokine 10 | Inflammation |
| cxcl11 | C-X-C motif chemokine 11 | Inflammation |
| CXCL16 | C-X-C motif chemokine 16 | Cardiovascular III |
| CXCL5 | C-X-C motif chemokine 5 | Inflammation |
| CXCL6 | C-X-C motif chemokine 6 | Inflammation |
| CXCL9 | C-X-C motif chemokine 9 | Inflammation |
| DCN | Decorin | Cardiovascular II |
| DECR1 | 2,4-dienoyl-CoA reductase, mitochondrial | Cardiovascular II |
| DKK1 | Dickkopf-related protein 1 | Cardiovascular II |
| DLK1 | Protein delta homolog 1 | Cardiovascular III |
| DNER | Delta and Notch-like epidermal growth factor-related receptor | Inflammation |
| EGFR | Epidermal growth factor receptor | Cardiovascular III |
| ENRAGE | Protein S100-A12 | Inflammation |
| EPCAM | Epithelial cell adhesion molecule | Cardiovascular III |
| EPHB4 | Ephrin type-B receptor 4 | Cardiovascular III |
| FABP2 | Fatty acid-binding protein, intestinal | Cardiovascular II |
| FABP4 | Fatty acid-binding protein, adipocyte | Cardiovascular III |
| FAZ | Tumor necrosis factor receptor superfamily member 6 | Cardiovascular III |
| FGF19 | Fibroblast growth factor 19 | Inflammation |
| FGF21 | Fibroblast growth factor 21 | Cardiovascular II, Inflammation |
| FGF23 | Fibroblast growth factor 23 | Cardiovascular II, Inflammation |
| FGF5 | Fibroblast growth factor 5 | Inflammation |
| FLT3L | Fms-related tyrosine kinase 3 ligand | Inflammation |
| FS | Follistatin | Cardiovascular II |
| GAL4 | Galectin-4 | Cardiovascular III |
| GAL9 | Galectin-9 | Cardiovascular II |
| GDF2 | Growth/differentiation factor 2 | Cardiovascular II |
| GDNF | Glial cell line-derived neurotrophic factor | Inflammation |
| GH | Somatotropin | Cardiovascular II |
| GIF | Cobalamin binding intrinsic factor | Cardiovascular II |
| GLO1 | Lactoylglutathione lyase | Cardiovascular II |
| GP6 | Platelet glycoprotein VI | Cardiovascular III |
| GRN | Progranulin | Cardiovascular III |
| GT | Gastrotropin | Cardiovascular II |
| HAOX1 | Hydroxyacid oxidase 1 | Cardiovascular II |
| HBEGF | Proheparin-binding EGF-like growth factor | Cardiovascular II |
| HGF | Hepatocyte growth factor | Inflammation |
| HO1 | Heme oxygenase 1 | Cardiovascular II |
| hOSCAR | Osteoclast-associated immunoglobulin-like receptor | Cardiovascular II |
| HSP27 | Heat shock protein beta-1 | Cardiovascular II |
| ICAM2 | Intercellular adhesion molecule 2 | Cardiovascular III |
| IDUA | Alpha-L-iduronidase | Cardiovascular II |
| IFNGAMMA | Interferon gamma | Inflammation |
| IGFBP1 | Insulin-like growth factor-binding protein 1 | Cardiovascular III |
| IGFBP2 | Insulin-like growth factor-binding protein 2 | Cardiovascular III |
| IGFBP7 | Insulin-like growth factor-binding protein 7 | Cardiovascular III |
| IgGFcreceptorIlb | Low affinity immunoglobulin gamma Fc region receptor II-b | Cardiovascular II |
| IL1alpha | Interleukin-1 alpha | Inflammation |
| IL10 | Interleukin-10 | Inflammation |
| IL10RA | Interleukin-10 receptor subunit alpha | Inflammation |
| IL10RB | Interleukin-10 receptor subunit beta | Inflammation |
| IL13 | Interleukin-13 | Inflammation |
| IL15RA | Interleukin-15 receptor subunit alpha | Inflammation |
| IL16 | Pro-interleukin-16 | Cardiovascular II |
| IL17A | Interleukin-17A | Inflammation |
| IL17C | Interleukin-17C | Inflammation |
| IL17D | Interleukin-17D | Cardiovascular II |
| IL17RA | Interleukin-17 receptor A | Cardiovascular III |
| IL18 | Interleukin-18 | Cardiovascular II, Inflammation |
| IL18BP | Interleukin-18-binding protein | Cardiovascular III |
| IL18R1 | Interleukin-18 receptor 1 | Inflammation |
| IL1ra | Interleukin-1 receptor antagonist protein | Cardiovascular II |
| IL1RL2 | Interleukin-1 receptor-like 2 | Cardiovascular II |
| IL1RT1 | Interleukin-1 receptor type 1 | Cardiovascular III |
| IL1RT2 | Interleukin-1 receptor type 2 | Cardiovascular III |
| IL2 | Interleukin-2 | Inflammation |
| IL20 | Interleukin-20 | Inflammation |
| IL20RA | Interleukin-20 receptor subunit alpha | Inflammation |
| IL21B | Interleukin-12 subunit beta | Inflammation |
| IL22RA1 | Interleukin-22 receptor subunit alpha-1 | Inflammation |
| IL24 | Interleukin-24 | Inflammation |
| IL27 | Interleukin-27 | Cardiovascular II |
| IL2RA | Interleukin-2 receptor subunit alpha | Cardiovascular III |
| IL2RB | Interleukin-2 receptor subunit beta | Inflammation |
| IL33 | Interleukin-33 | Inflammation |
| IL4 | Interleukin-4 | Inflammation |
| IL4RA | Interleukin-4 receptor subunit alpha | Cardiovascular II |
| IL5 | Interleukin-5 | Inflammation |
| IL6 | Interleukin-6 | Cardiovascular II, Inflammation |
| IL6RA | Interleukin-6 receptor subunit alpha | Cardiovascular III |
| IL7 | Interleukin-7 | Inflammation |
| IL8 | Interleukin-8 | Inflammation |
| ITGB1BP2 | Integrin beta-1-binding protein 2 | Cardiovascular II |
| ITGB2 | Integrin beta-2 | Cardiovascular III |
| JAMA | Junctional adhesion molecule A | Cardiovascular III |
| KIM1 | Hepatitis A virus cellular receptor 1 | Cardiovascular II |
| KLK6 | Kallikrein-6 | Cardiovascular III |
| LAPTGFbeta1 | Transforming growth factor beta-1 proprotein | Inflammation |
| LDLRECEPTOR | Low-density lipoprotein receptor | Cardiovascular III |
| LEP | Leptin | Cardiovascular II |
| LIF | Leukemia inhibitory factor | Inflammation |
| LIFR | Leukemia inhibitory factor receptor | Inflammation |
| LOX1 | Oxidized low-density lipoprotein receptor 1 | Cardiovascular II |
| LPL | Lipoprotein lipase | Cardiovascular II |
| LTBR | Tumor necrosis factor receptor superfamily member 3 | Cardiovascular III |
| MARCO | Macrophage receptor MARCO | Cardiovascular II |
| MB | Myoglobin | Cardiovascular III |
| MCP1 | C-C motif chemokine 2 | Cardiovascular III, Inflammation |
| MCP2 | C-C motif chemokine 8 | Inflammation |
| MCP3 | C-C motif chemokine 7 | Inflammation |
| MCP4 | C-C motif chemokine 13 | Inflammation |
| MEPE | Matrix extracellular phosphoglycoprotein | Cardiovascular III |
| MERTK | Tyrosine-protein kinase Mer | Cardiovascular II |
| MMP1 | Interstitial collagenase | Inflammation |
| MMP10 | Stromelysin-2 | Inflammation |
| MMP12 | Macrophage metalloelastase | Cardiovascular II |
| MMP2 | 72 kDa type IV collagenase | Cardiovascular III |
| MMP3 | Stromelysin-1 | Cardiovascular III |
| MMP7 | Matrilysin | Cardiovascular II |
| MMP9 | Matrix metalloproteinase-9 | Cardiovascular III |
| MPO | Myeloperoxidase | Cardiovascular III |
| NEMO | NF-kappa-B essential modulator | Cardiovascular II |
| NOTCH3 | Neurogenic locus notch homolog protein 3 | Cardiovascular III |
| NRTN | Neurturin | Inflammation |
| NT3 | Neurotrophin-3 | Inflammation |
| OPG | Tumor necrosis factor receptor superfamily member 11B | Cardiovascular III, Inflammation |
| OPN | Osteopontin | Cardiovascular III |
| OSM | Oncostatin-M | Inflammation |
| PAI | Plasminogen activator inhibitor 1 | Cardiovascular III |
| PAPPA | Pappalysin-1 | Cardiovascular II |
| PAR1 | Proteinase-activated receptor 1 | Cardiovascular II |
| PARP1 | Poly [ADP-ribose] polymerase 1 | Cardiovascular II |
| PCSK9 | Proprotein convertase subtilisin/kexin type 9 | Cardiovascular III |
| PDGFsubunitA | Platelet-derived growth factor subunit A | Cardiovascular III |
| PDGFsubunitB | Platelet-derived growth factor subunit B | Cardiovascular II |
| PDL1 | Programmed cell death 1 ligand 1 | Inflammation |
| PDL2 | Programmed cell death 1 ligand 2 | Cardiovascular II |
| PECAM1 | Platelet endothelial cell adhesion molecule | Cardiovascular III |
| PGF | Placenta growth factor | Cardiovascular II |
| PGLYRP1 | Peptidoglycan recognition protein 1 | Cardiovascular III |
| PI3 | Elafin | Cardiovascular III |
| PIGR | Polymeric immunoglobulin receptor | Cardiovascular II |
| PLC | Basement membrane-specific heparan sulfate proteoglycan core protein | Cardiovascular III |
| PON3 | Serum paraoxonase/lactonase 3 | Cardiovascular III |
| PRELP | Prolargin | Cardiovascular II |
| PRSS27 | Serine protease 27 | Cardiovascular II |
| PRSS8 | Prostasin | Cardiovascular II |
| PRTN3 | Myeloblastin | Cardiovascular III |
| PSGL1 | P-selectin glycoprotein ligand 1 | Cardiovascular II |
| PSPD | Pulmonary surfactant-associated protein D | Cardiovascular III |
| PTX3 | Pentraxin-related protein PTX3 | Cardiovascular II |
| RAGE | Advanced glycosylation end product-specific receptor | Cardiovascular II |
| RARRES2 | Retinoic acid receptor responder protein 2 | Cardiovascular III |
| REN | Renin | Cardiovascular II |
| RETN | Resistin | Cardiovascular III |
| SCF | Kit ligand | Cardiovascular II, Inflammation |
| SCGB3A2 | Secretoglobin family 3A member 2 | Cardiovascular III |
| SELE | E-selectin | Cardiovascular III |
| SELP | P-selectin | Cardiovascular III |
| SERPINA12 | Serpin A12 | Cardiovascular II |
| SHPS1 | Tyrosine-protein phosphatase non-receptor type substrate 1 | Cardiovascular III |
| SIRT2 | NAD-dependent protein deacetylase sirtuin-2 | Inflammation |
| SLAMF1 | Signaling lymphocytic activation molecule | Inflammation |
| SLAMF7 | SLAM family member 7 | Cardiovascular II |
| SOD2 | Superoxide dismutase [Mn], mitochondrial | Cardiovascular II |
| SORT1 | Sortilin | Cardiovascular II |
| SPON1 | Spondin-1 | Cardiovascular III |
| SPON2 | Spondin-2 | Cardiovascular II |
| SRC | roto-oncogene tyrosine-protein kinase Src | Cardiovascular II |
| ST1A1 | Sulfotransferase 1A1 | Inflammation |
| ST2 | Interleukin-1 receptor-like 1 | Cardiovascular III |
| STAMBP | STAM-binding protein | Inflammation |
| STK4 | Serine/threonine-protein kinase 4 | Cardiovascular II |
| TF | Tissue factor | Cardiovascular II |
| TFF3 | Trefoil factor 3 | Cardiovascular III |
| TFPI | Tissue factor pathway inhibitor | Cardiovascular III |
| TGFALPHA | Protransforming growth factor alpha | Inflammation |
| TGM2 | Protein-glutamine gamma-glutamyltransferase 2 | Cardiovascular II |
| THBS2 | Thrombospondin-2 | Cardiovascular II |
| THPO | Thrombopoietin | Cardiovascular II |
| TIE2 | Angiopoietin-1 receptor | Cardiovascular II |
| TIMP4 | Metalloproteinase inhibitor 4 | Cardiovascular III |
| TLT2 | Trem-like transcript 2 protein | Cardiovascular III |
| TM | Thrombomodulin | Cardiovascular II |
| TNF | Tumor necrosis factor | Inflammation |
| TNFB | Lymphotoxin-alpha | Inflammation |
| TNFR1 | Tumor necrosis factor receptor superfamily member 1A | Cardiovascular III |
| TNFR2 | Tumor necrosis factor receptor superfamily member 1B | Cardiovascular III |
| TNFRSF10A | Tumor necrosis factor receptor superfamily member 10A | Cardiovascular II |
| TNFRSF10C | Tumor necrosis factor receptor superfamily member 10C | Cardiovascular III |
| TNFRSF11A | Tumor necrosis factor receptor superfamily member 11A | Cardiovascular II |
| TNFRSF13B | Tumor necrosis factor receptor superfamily member 13B | Cardiovascular II |
| TNFRSF14 | Tumor necrosis factor receptor superfamily member 14 | Cardiovascular III |
| TNFRSF9 | Tumor necrosis factor receptor superfamily member 9 | Inflammation |
| TNFSF13B | Tumor necrosis factor ligand superfamily member 13B | Cardiovascular III |
| TNFSF14 | Tumor necrosis factor ligand superfamily member 14 | Inflammation |
| TPA | Tissue-type plasminogen activator | Cardiovascular III |
| TR | Transferrin receptor protein 1 | Cardiovascular III |
| TRAIL | Tumor necrosis factor ligand superfamily member 10 | Inflammation |
| TRAILR2 | Tumor necrosis factor receptor superfamily member 10B | Cardiovascular II |
| TRANCE | Tumor necrosis factor ligand superfamily member 11 | Inflammation |
| TRAP | Tartrate-resistant acid phosphatase type 5 | Cardiovascular III |
| TSLP | Thymic stromal lymphopoietin | Inflammation |
| TWEAK | Tumor necrosis factor ligand superfamily member 12 | Inflammation |
| UPA | Urokinase-type plasminogen activator | Cardiovascular III, Inflammation |
| UPAR | Urokinase plasminogen activator surface receptor | Cardiovascular III |
| VEGFA | Vascular endothelial growth factor A | Inflammation |
| VEGFD | Vascular endothelial growth factor D | Cardiovascular II |
| VSIG2 | V-set and immunoglobulin domain-containing protein 2 | Cardiovascular II |
| VWF | von Willebrand factor | Cardiovascular III |
| XCL1 | Lymphotactin | Cardiovascular II |

Supplemental table 2 – Absolute and relative frequencies of missing clinical, analytical and echocardiographic data.

| Variable | No. missing (%) |
| --- | --- |
| Sex | 0 |
| Age (years) | 0 |
| Body Mass Index (kg/m^2^) | 2 (0.4%) |
| Waist circumference (cm) | 17 (3.2%) |
| Current or past smoker | 5 (0.9%) |
| Arterial Hypertension | 0 |
| Diabetes mellitus | 0 |
| Stroke/Transient ischemic attack | 0 |
| Chronic obstructive pulmonar disease | 0 |
| Standing heart rate (bpm) | 11 (2.1%) |
| Standing systolic blood pressure (mmHg) | 11 (2.1%) |
| Standing diastolic blood pressure (mmHg) | 11 (2.1%) |
| Potassium (mmol/L) | 5 (0.9%) |
| Haemoglobin (g/dL) | 2 (0.4%) |
| Cholesterol (mg/dL) | 114 (21.6%) |
| Creatinine (µmol/L)) | 0 |
| Urea (mmol/l) | 1 (0.2%) |
| eGFR-MDRD (ml/min/1.73m^2^) | 1 (0.2%) |
| Aspirin | 0 |
| Beta Blocker | 0 |
| Thiazides | 0 |
| ACEI or ARB | 0 |
| Statin/Lipid lowering drug | 0 |
| Calcium-channel blocker | 0 |
| Anticoagulant | 3 (0.6%) |
| Antiplatelet (excluding aspirin) | 0 |
| Galectin-3 (µg/L) | 9 (1.7%) |
| Growth differentiation factor 15 (ng/L) | 9 (1.7%) |
| NT-proBNP (ng/L) | 9 (1.7%) |
| MMP-1 (µg/L) | 11 (2.1%) |
| PIIINP (µg/L) | 15 (2.8%) |
| PICP (µg/L) | 10 (1.9%) |
| CITP (µg/L) | 9 (1.7%) |
| QRS duration (ms) | 10 (1.9%) |
| Left end-diastolic volume index (mL/m^2^) | 71 (13.4%) |
| Left ventricular ejection fraction Biplane (%) | 163 (30.9%) |
| Left Ventricular Mass index (g/m^2^) | 54 (10.2%) |
| Left atrial volume index (mL/m^2^) | 96 (18.2%) |
| Early wave mitral valve flow velocity E (m/s) | 32 (6.1%) |
| Late wave mitral valve flow velocity A (m/s) | 34 (6.5%) |
| E/A ratio | 32 (6.1%) |
| E/ early diastolic tissue velocity (e’) ratio | 62 (11.8%) |
| Tricuspid annular plane systolic excursion (mm) | 59 (11.2%) |

ACE – angiotensin-converting enzyme inhibitors; ARB - angiotensin receptor blockers; bpm – beats per minute; CITP - Collagen Type-1 C-terminal telopeptide; eGFR-MDRD – estimated glomerular filtration rate, using Modification of Diet in Renal Disease formula; NT-proBNP - N-terminal pro-brain natriuretic peptide; MMP-1 - Matrix Metalloproteinase-1; PICP - Procollagen type-I C-terminal pro-peptide; PIIINP - procollagen type-III N-terminal pro-peptide.

Supplemental table 3 – Absolute and relative frequencies of missing protein data. Full names of proteins can be found in *supplemental table 1*.

| Protein | No. missing at baseline (%) | No. missing after 1 month (%) | No. missing after 9 months (%) |
| --- | --- | --- | --- |
| NTproBNP | 9 (1.7%) | 42 (8%) | 32 (6.1%) |
| GAL3 | 9 (1.7%) | 41 (7.8%) | 37 (7%) |
| HSTNT | 9 (1.7%) | 42 (8%) | 32 (6.1%) |
| GDF15 | 9 (1.7%) | 42 (8%) | 32 (6.1%) |
| CITP | 9 (1.7%) | 41 (7.8%) | 31 (5.9%) |
| PICP | 10 (1.9%) | 42 (8%) | 32 (6.1%) |
| PIIINP | 15 (2.8%) | 42 (8%) | 32 (6.1%) |
| BMP6 | 11 (2.1 %) | 37 (7%) | 29 (5.5%) |
| ANG1 | 11 (2.1 %) | 37 (7%) | 29 (5.5%) |
| ADM | 11 (2.1 %) | 37 (7%) | 29 (5.5%) |
| CD40L | 11 (2.1 %) | 37 (7%) | 29 (5.5%) |
| SLAMF7 | 11 (2.1 %) | 37 (7%) | 29 (5.5%) |
| PGF | 11 (2.1 %) | 37 (7%) | 29 (5.5%) |
| ADAMTS13 | 11 (2.1 %) | 37 (7%) | 29 (5.5%) |
| BOC | 11 (2.1 %) | 37 (7%) | 29 (5.5%) |
| IL4RA | 11 (2.1 %) | 37 (7%) | 29 (5.5%) |
| SRC | 11 (2.1 %) | 37 (7%) | 29 (5.5%) |
| IL1RA | 11 (2.1 %) | 37 (7%) | 29 (5.5%) |
| IL6 | 11 (2.1 %) | 37 (7%) | 29 (5.5%) |
| TNFRSF10A | 11 (2.1 %) | 37 (7%) | 29 (5.5%) |
| STK4 | 11 (2.1 %) | 37 (7%) | 29 (5.5%) |
| IDUA | 11 (2.1 %) | 37 (7%) | 29 (5.5%) |
| TNFRSF11A | 11 (2.1 %) | 37 (7%) | 29 (5.5%) |
| PAR1 | 11 (2.1 %) | 37 (7%) | 29 (5.5%) |
| TRAILR2 | 11 (2.1 %) | 37 (7%) | 29 (5.5%) |
| PRSS27 | 11 (2.1 %) | 37 (7%) | 29 (5.5%) |
| TIE2 | 11 (2.1 %) | 37 (7%) | 29 (5.5%) |
| TF | 11 (2.1 %) | 37 (7%) | 29 (5.5%) |
| IL1RL2 | 11 (2.1 %) | 37 (7%) | 29 (5.5%) |
| PDGFsubunitB | 11 (2.1 %) | 37 (7%) | 29 (5.5%) |
| IL27 | 11 (2.1 %) | 37 (7%) | 29 (5.5%) |
| IL17D | 11 (2.1 %) | 37 (7%) | 29 (5.5%) |
| CXCL1 | 11 (2.1 %) | 37 (7%) | 29 (5.5%) |
| LOX1 | 11 (2.1 %) | 37 (7%) | 29 (5.5%) |
| GAL9 | 11 (2.1 %) | 37 (7%) | 29 (5.5%) |
| GIF | 11 (2.1 %) | 37 (7%) | 29 (5.5%) |
| SCF | 11 (2.1 %) | 37 (7%) | 29 (5.5%) |
| IL18 | 11 (2.1 %) | 37 (7%) | 29 (5.5%) |
| FGF21 | 11 (2.1 %) | 37 (7%) | 29 (5.5%) |
| PIGR | 11 (2.1 %) | 37 (7%) | 29 (5.5%) |
| RAGE | 11 (2.1 %) | 37 (7%) | 29 (5.5%) |
| SOD2 | 11 (2.1 %) | 37 (7%) | 29 (5.5%) |
| CTRC | 11 (2.1 %) | 37 (7%) | 29 (5.5%) |
| FGF23 | 11 (2.1 %) | 37 (7%) | 29 (5.5%) |
| SPON2 | 11 (2.1 %) | 37 (7%) | 29 (5.5%) |
| GH | 11 (2.1 %) | 37 (7%) | 29 (5.5%) |
| FS | 11 (2.1 %) | 37 (7%) | 29 (5.5%) |
| GLO1 | 11 (2.1 %) | 37 (7%) | 29 (5.5%) |
| CD84 | 11 (2.1 %) | 37 (7%) | 29 (5.5%) |
| PAPPA | 11 (2.1 %) | 37 (7%) | 29 (5.5%) |
| SERPINA12 | 11 (2.1 %) | 37 (7%) | 29 (5.5%) |
| REN | 11 (2.1 %) | 37 (7%) | 29 (5.5%) |
| DECR1 | 11 (2.1 %) | 37 (7%) | 29 (5.5%) |
| MERTK | 11 (2.1 %) | 37 (7%) | 29 (5.5%) |
| KIM1 | 11 (2.1 %) | 37 (7%) | 29 (5.5%) |
| THBS2 | 11 (2.1 %) | 37 (7%) | 29 (5.5%) |
| TM | 11 (2.1 %) | 37 (7%) | 29 (5.5%) |
| VSIG2 | 11 (2.1 %) | 37 (7%) | 29 (5.5%) |
| AMBP | 11 (2.1 %) | 37 (7%) | 29 (5.5%) |
| PRELP | 11 (2.1 %) | 37 (7%) | 29 (5.5%) |
| HO1 | 11 (2.1 %) | 37 (7%) | 29 (5.5%) |
| XCL1 | 11 (2.1 %) | 37 (7%) | 29 (5.5%) |
| IL16 | 11 (2.1 %) | 37 (7%) | 29 (5.5%) |
| SORT1 | 11 (2.1 %) | 37 (7%) | 29 (5.5%) |
| CEACAM8 | 11 (2.1 %) | 37 (7%) | 29 (5.5%) |
| PTX3 | 11 (2.1 %) | 37 (7%) | 29 (5.5%) |
| PSGL1 | 11 (2.1 %) | 37 (7%) | 29 (5.5%) |
| CCL17 | 11 (2.1 %) | 37 (7%) | 29 (5.5%) |
| CCL33 | 11 (2.1 %) | 37 (7%) | 29 (5.5%) |
| MMP7 | 11 (2.1 %) | 37 (7%) | 29 (5.5%) |
| IgGFcreceptorIlb | 11 (2.1 %) | 37 (7%) | 29 (5.5%) |
| ITGB1BP2 | 11 (2.1 %) | 37 (7%) | 29 (5.5%) |
| DCN | 11 (2.1 %) | 37 (7%) | 29 (5.5%) |
| DKK1 | 11 (2.1 %) | 37 (7%) | 29 (5.5%) |
| LPL | 11 (2.1 %) | 37 (7%) | 29 (5.5%) |
| PRSS8 | 11 (2.1 %) | 37 (7%) | 29 (5.5%) |
| AGRP | 11 (2.1 %) | 37 (7%) | 29 (5.5%) |
| HBEGF | 11 (2.1 %) | 37 (7%) | 29 (5.5%) |
| GDF2 | 11 (2.1 %) | 37 (7%) | 29 (5.5%) |
| FABP2 | 11 (2.1 %) | 37 (7%) | 29 (5.5%) |
| THPO | 11 (2.1 %) | 37 (7%) | 29 (5.5%) |
| MARCO | 11 (2.1 %) | 37 (7%) | 29 (5.5%) |
| GT | 11 (2.1 %) | 37 (7%) | 29 (5.5%) |
| MMP12 | 11 (2.1 %) | 37 (7%) | 29 (5.5%) |
| ACE2 | 11 (2.1 %) | 37 (7%) | 29 (5.5%) |
| PDL2 | 11 (2.1 %) | 37 (7%) | 29 (5.5%) |
| CTSL1 | 11 (2.1 %) | 37 (7%) | 29 (5.5%) |
| hOSCAR | 11 (2.1 %) | 37 (7%) | 29 (5.5%) |
| TNFRSF13B | 11 (2.1 %) | 37 (7%) | 29 (5.5%) |
| TGM2 | 11 (2.1 %) | 37 (7%) | 29 (5.5%) |
| LEP | 11 (2.1 %) | 37 (7%) | 29 (5.5%) |
| CA5A | 11 (2.1 %) | 37 (7%) | 29 (5.5%) |
| HSP27 | 11 (2.1 %) | 37 (7%) | 29 (5.5%) |
| CD4 | 11 (2.1 %) | 37 (7%) | 29 (5.5%) |
| NEMO | 11 (2.1 %) | 37 (7%) | 29 (5.5%) |
| VEGFD | 11 (2.1 %) | 37 (7%) | 29 (5.5%) |
| PARP1 | 11 (2.1 %) | 37 (7%) | 29 (5.5%) |
| HAOX1 | 11 (2.1 %) | 37 (7%) | 29 (5.5%) |
| TNFRSF14 | 11 (2.1 %) | 37 (7%) | 29 (5.5%) |
| LDLRECEPTOR | 11 (2.1 %) | 37 (7%) | 29 (5.5%) |
| ITGB2 | 11 (2.1 %) | 37 (7%) | 29 (5.5%) |
| IL17RA | 11 (2.1 %) | 37 (7%) | 29 (5.5%) |
| TNFR2 | 11 (2.1 %) | 37 (7%) | 29 (5.5%) |
| MMP9 | 11 (2.1 %) | 37 (7%) | 29 (5.5%) |
| EPHB4 | 11 (2.1 %) | 37 (7%) | 29 (5.5%) |
| IL2RA | 11 (2.1 %) | 37 (7%) | 29 (5.5%) |
| OPG | 11 (2.1 %) | 37 (7%) | 29 (5.5%) |
| ALCAM | 11 (2.1 %) | 37 (7%) | 29 (5.5%) |
| TFF3 | 11 (2.1 %) | 37 (7%) | 29 (5.5%) |
| SELP | 11 (2.1 %) | 37 (7%) | 29 (5.5%) |
| CSTB | 11 (2.1 %) | 37 (7%) | 29 (5.5%) |
| MCP1 | 11 (2.1 %) | 37 (7%) | 29 (5.5%) |
| CD163 | 11 (2.1 %) | 37 (7%) | 29 (5.5%) |
| GRN | 11 (2.1 %) | 37 (7%) | 29 (5.5%) |
| BLMHYDROLASE | 11 (2.1 %) | 37 (7%) | 29 (5.5%) |
| PLC | 11 (2.1 %) | 37 (7%) | 29 (5.5%) |
| LTBER | 11 (2.1 %) | 37 (7%) | 29 (5.5%) |
| NOTCH3 | 11 (2.1 %) | 37 (7%) | 29 (5.5%) |
| TIMP4 | 11 (2.1 %) | 37 (7%) | 29 (5.5%) |
| CNTN1 | 11 (2.1 %) | 37 (7%) | 29 (5.5%) |
| CDH5 | 11 (2.1 %) | 37 (7%) | 29 (5.5%) |
| TLT2 | 11 (2.1 %) | 37 (7%) | 29 (5.5%) |
| FABP4 | 11 (2.1 %) | 37 (7%) | 29 (5.5%) |
| TFPI | 11 (2.1 %) | 37 (7%) | 29 (5.5%) |
| PAI | 11 (2.1 %) | 37 (7%) | 29 (5.5%) |
| CCL24 | 11 (2.1 %) | 37 (7%) | 29 (5.5%) |
| TR | 11 (2.1 %) | 37 (7%) | 29 (5.5%) |
| RBFRSF10C | 11 (2.1 %) | 37 (7%) | 29 (5.5%) |
| SELE | 11 (2.1 %) | 37 (7%) | 29 (5.5%) |
| AZU1 | 11 (2.1 %) | 37 (7%) | 29 (5.5%) |
| DLK1 | 11 (2.1 %) | 37 (7%) | 29 (5.5%) |
| SPON1 | 11 (2.1 %) | 37 (7%) | 29 (5.5%) |
| MPO | 11 (2.1 %) | 37 (7%) | 29 (5.5%) |
| CXCL16 | 11 (2.1 %) | 37 (7%) | 29 (5.5%) |
| IL6RA | 11 (2.1 %) | 37 (7%) | 29 (5.5%) |
| RETN | 11 (2.1 %) | 37 (7%) | 29 (5.5%) |
| IGFBP1 | 11 (2.1 %) | 37 (7%) | 29 (5.5%) |
| CHIT1 | 11 (2.1 %) | 37 (7%) | 29 (5.5%) |
| TRAP | 11 (2.1 %) | 37 (7%) | 29 (5.5%) |
| GP6 | 11 (2.1 %) | 37 (7%) | 29 (5.5%) |
| PSPD | 11 (2.1 %) | 37 (7%) | 29 (5.5%) |
| PI3 | 11 (2.1 %) | 37 (7%) | 29 (5.5%) |
| EPCAM | 11 (2.1 %) | 37 (7%) | 29 (5.5%) |
| APN | 11 (2.1 %) | 37 (7%) | 29 (5.5%) |
| AXL | 11 (2.1 %) | 37 (7%) | 29 (5.5%) |
| IL1RT1 | 11 (2.1 %) | 37 (7%) | 29 (5.5%) |
| MMP2 | 11 (2.1 %) | 37 (7%) | 29 (5.5%) |
| FAS | 11 (2.1 %) | 37 (7%) | 29 (5.5%) |
| MB | 11 (2.1 %) | 37 (7%) | 29 (5.5%) |
| TNFSF13B | 11 (2.1 %) | 37 (7%) | 29 (5.5%) |
| PRTN3 | 11 (2.1 %) | 37 (7%) | 29 (5.5%) |
| PCSK9 | 11 (2.1 %) | 37 (7%) | 29 (5.5%) |
| UPAR | 11 (2.1 %) | 37 (7%) | 29 (5.5%) |
| OPN | 11 (2.1 %) | 37 (7%) | 29 (5.5%) |
| CTSD | 11 (2.1 %) | 37 (7%) | 29 (5.5%) |
| PGLYRP1 | 11 (2.1 %) | 37 (7%) | 29 (5.5%) |
| CPA1 | 11 (2.1 %) | 37 (7%) | 29 (5.5%) |
| JAMA | 11 (2.1 %) | 37 (7%) | 29 (5.5%) |
| GAL4 | 11 (2.1 %) | 37 (7%) | 29 (5.5%) |
| IL1RT2 | 11 (2.1 %) | 37 (7%) | 29 (5.5%) |
| SHPS1 | 11 (2.1 %) | 37 (7%) | 29 (5.5%) |
| CCL15 | 11 (2.1 %) | 37 (7%) | 29 (5.5%) |
| CASP3 | 11 (2.1 %) | 37 (7%) | 29 (5.5%) |
| UPA | 11 (2.1 %) | 37 (7%) | 29 (5.5%) |
| CPB1 | 11 (2.1 %) | 37 (7%) | 29 (5.5%) |
| CHI3L1 | 11 (2.1 %) | 37 (7%) | 29 (5.5%) |
| ST2 | 11 (2.1 %) | 37 (7%) | 29 (5.5%) |
| TPA | 11 (2.1 %) | 37 (7%) | 29 (5.5%) |
| SCGB3A2 | 11 (2.1 %) | 37 (7%) | 29 (5.5%) |
| EGFR | 11 (2.1 %) | 37 (7%) | 29 (5.5%) |
| IGFBP7 | 11 (2.1 %) | 37 (7%) | 29 (5.5%) |
| CD93 | 11 (2.1 %) | 37 (7%) | 29 (5.5%) |
| IL18BP | 11 (2.1 %) | 37 (7%) | 29 (5.5%) |
| COL1A1 | 11 (2.1 %) | 37 (7%) | 29 (5.5%) |
| PON3 | 11 (2.1 %) | 37 (7%) | 29 (5.5%) |
| CTSZ | 11 (2.1 %) | 37 (7%) | 29 (5.5%) |
| MMP3 | 11 (2.1 %) | 37 (7%) | 29 (5.5%) |
| RARRES2 | 11 (2.1 %) | 37 (7%) | 29 (5.5%) |
| ICAM2 | 11 (2.1 %) | 37 (7%) | 29 (5.5%) |
| KLK6 | 11 (2.1 %) | 37 (7%) | 29 (5.5%) |
| PDGFsubunitA | 11 (2.1 %) | 37 (7%) | 29 (5.5%) |
| TNFR1 | 11 (2.1 %) | 37 (7%) | 29 (5.5%) |
| IGFBP2 | 11 (2.1 %) | 37 (7%) | 29 (5.5%) |
| VWF | 11 (2.1 %) | 37 (7%) | 29 (5.5%) |
| PECAM1 | 11 (2.1 %) | 37 (7%) | 29 (5.5%) |
| MEPE | 11 (2.1 %) | 37 (7%) | 29 (5.5%) |
| CCL16 | 11 (2.1 %) | 37 (7%) | 29 (5.5%) |
| IL8 | 11 (2.1 %) | 37 (7%) | 29 (5.5%) |
| VEGFA | 11 (2.1 %) | 37 (7%) | 29 (5.5%) |
| CD8A | 11 (2.1 %) | 37 (7%) | 29 (5.5%) |
| MCP3 | 11 (2.1 %) | 37 (7%) | 29 (5.5%) |
| GDNF | 11 (2.1 %) | 37 (7%) | 29 (5.5%) |
| CDCP1 | 11 (2.1 %) | 37 (7%) | 29 (5.5%) |
| CD244 | 11 (2.1 %) | 37 (7%) | 29 (5.5%) |
| IL7 | 11 (2.1 %) | 37 (7%) | 29 (5.5%) |
| LAPTGFbeta1 | 11 (2.1 %) | 37 (7%) | 29 (5.5%) |
| IL17C | 11 (2.1 %) | 37 (7%) | 29 (5.5%) |
| IL17A | 11 (2.1 %) | 37 (7%) | 29 (5.5%) |
| CXCL11 | 11 (2.1 %) | 37 (7%) | 29 (5.5%) |
| AXIN1 | 11 (2.1 %) | 37 (7%) | 29 (5.5%) |
| TRAIL | 11 (2.1 %) | 37 (7%) | 29 (5.5%) |
| IL20RA | 11 (2.1 %) | 37 (7%) | 29 (5.5%) |
| CXCL9 | 11 (2.1 %) | 37 (7%) | 29 (5.5%) |
| CST5 | 11 (2.1 %) | 37 (7%) | 29 (5.5%) |
| IL2RB | 11 (2.1 %) | 37 (7%) | 29 (5.5%) |
| IL1alpha | 11 (2.1 %) | 37 (7%) | 29 (5.5%) |
| OSM | 11 (2.1 %) | 37 (7%) | 29 (5.5%) |
| IL2 | 11 (2.1 %) | 37 (7%) | 29 (5.5%) |
| TSLP | 11 (2.1 %) | 37 (7%) | 29 (5.5%) |
| CCL4 | 11 (2.1 %) | 37 (7%) | 29 (5.5%) |
| CD6 | 11 (2.1 %) | 37 (7%) | 29 (5.5%) |
| SLAMF1 | 11 (2.1 %) | 37 (7%) | 29 (5.5%) |
| TGFALPHA | 11 (2.1 %) | 37 (7%) | 29 (5.5%) |
| MCP4 | 11 (2.1 %) | 37 (7%) | 29 (5.5%) |
| CCL11 | 11 (2.1 %) | 37 (7%) | 29 (5.5%) |
| TNFSF14 | 11 (2.1 %) | 37 (7%) | 29 (5.5%) |
| IL10RA | 11 (2.1 %) | 37 (7%) | 29 (5.5%) |
| GFG5 | 11 (2.1 %) | 37 (7%) | 29 (5.5%) |
| MMP1 | 11 (2.1 %) | 37 (7%) | 29 (5.5%) |
| LIFR | 11 (2.1 %) | 37 (7%) | 29 (5.5%) |
| CCL19 | 11 (2.1 %) | 37 (7%) | 29 (5.5%) |
| IL15RA | 11 (2.1 %) | 37 (7%) | 29 (5.5%) |
| IL10RB | 11 (2.1 %) | 37 (7%) | 29 (5.5%) |
| IL22RA1 | 11 (2.1 %) | 37 (7%) | 29 (5.5%) |
| IL18R1 | 11 (2.1 %) | 37 (7%) | 29 (5.5%) |
| PDL1 | 11 (2.1 %) | 37 (7%) | 29 (5.5%) |
| BetaNGF | 11 (2.1 %) | 37 (7%) | 29 (5.5%) |
| CXCL5 | 11 (2.1 %) | 37 (7%) | 29 (5.5%) |
| TRANCE | 11 (2.1 %) | 37 (7%) | 29 (5.5%) |
| HGF | 11 (2.1 %) | 37 (7%) | 29 (5.5%) |
| IL12B | 11 (2.1 %) | 37 (7%) | 29 (5.5%) |
| IL24 | 11 (2.1 %) | 37 (7%) | 29 (5.5%) |
| IL13 | 11 (2.1 %) | 37 (7%) | 29 (5.5%) |
| ARTN | 11 (2.1 %) | 37 (7%) | 29 (5.5%) |
| MMP10 | 11 (2.1 %) | 37 (7%) | 29 (5.5%) |
| IL10 | 11 (2.1 %) | 37 (7%) | 29 (5.5%) |
| TNF | 11 (2.1 %) | 37 (7%) | 29 (5.5%) |
| CCL23 | 11 (2.1 %) | 37 (7%) | 29 (5.5%) |
| CD5 | 11 (2.1 %) | 37 (7%) | 29 (5.5%) |
| FLT3L | 11 (2.1 %) | 37 (7%) | 29 (5.5%) |
| CXCL6 | 11 (2.1 %) | 37 (7%) | 29 (5.5%) |
| CXCL10 | 11 (2.1 %) | 37 (7%) | 29 (5.5%) |
| BP14E | 11 (2.1 %) | 37 (7%) | 29 (5.5%) |
| IL20 | 11 (2.1 %) | 37 (7%) | 29 (5.5%) |
| SIRT2 | 11 (2.1 %) | 37 (7%) | 29 (5.5%) |
| CCL28 | 11 (2.1 %) | 37 (7%) | 29 (5.5%) |
| DNER | 11 (2.1 %) | 37 (7%) | 29 (5.5%) |
| ENRAGE | 11 (2.1 %) | 37 (7%) | 29 (5.5%) |
| CD40 | 11 (2.1 %) | 37 (7%) | 29 (5.5%) |
| IL33 | 11 (2.1 %) | 37 (7%) | 29 (5.5%) |
| IFNGAMMA | 11 (2.1 %) | 37 (7%) | 29 (5.5%) |
| FGF19 | 11 (2.1 %) | 37 (7%) | 29 (5.5%) |
| IL4 | 11 (2.1 %) | 37 (7%) | 29 (5.5%) |
| LIF | 11 (2.1 %) | 37 (7%) | 29 (5.5%) |
| NRTN | 11 (2.1 %) | 37 (7%) | 29 (5.5%) |
| MCP2 | 11 (2.1 %) | 37 (7%) | 29 (5.5%) |
| CASP8 | 11 (2.1 %) | 37 (7%) | 29 (5.5%) |
| CCL25 | 11 (2.1 %) | 37 (7%) | 29 (5.5%) |
| CX3CL1 | 11 (2.1 %) | 37 (7%) | 29 (5.5%) |
| TNFRSF9 | 11 (2.1 %) | 37 (7%) | 29 (5.5%) |
| NT3 | 11 (2.1 %) | 37 (7%) | 29 (5.5%) |
| TWEAK | 11 (2.1 %) | 37 (7%) | 29 (5.5%) |
| CCL20 | 11 (2.1 %) | 37 (7%) | 29 (5.5%) |
| ST1A1 | 11 (2.1 %) | 37 (7%) | 29 (5.5%) |
| STAMBP | 11 (2.1 %) | 37 (7%) | 29 (5.5%) |
| IL5 | 11 (2.1 %) | 37 (7%) | 29 (5.5%) |
| ADA | 11 (2.1 %) | 37 (7%) | 29 (5.5%) |
| TNFB | 11 (2.1 %) | 37 (7%) | 29 (5.5%) |
| CSF1 | 11 (2.1 %) | 37 (7%) | 29 (5.5%) |

Supplemental table 4 - Proteins included in the logistic regression model, adjusted to the defined clinically relevant variables, comparing “CAD” *versus* “no CAD/no MI” participants. Full names of proteins can be found in *supplemental table 1*.

| Protein | AIC | r^2^ | OR (95% CI) | Z | *p*-value | FDR adjusted *p*-value | Proportion of significant FDR adjusted p-values (bootstraped models) |
| --- | --- | --- | --- | --- | --- | --- | --- |
| MMP7 | 288.1 | 0.566 | 4.37 (2.32, 8.85) | 4.3 | <0.001 | 0.004 | 1.00 |
| GAL4 | 298.0 | 0.542 | 3.06 (1.66, 5.83) | 3.5 | <0.001 | 0.06 | 0.96 |
| OSM | 302.0 | 0.531 | 0.55 (0.37, 0.81) | -3.0 | 0.003 | 0.22 |  |
| PAI | 302.2 | 0.531 | 1.65 (1.19, 2.31) | 2.9 | 0.003 | 0.22 | 0.81 |
| TGFALPHA | 302.7 | 0.530 | 0.25 (0.10, 0.64) | -2.9 | 0.004 | 0.22 |  |
| TRANCE | 303.4 | 0.528 | 0.51 (0.31, 0.81) | -2.8 | 0.006 | 0.22 |  |
| PGLYR1 | 303.2 | 0.529 | 0.42 (0.23, 0.77) | -2.8 | 0.006 | 0.22 | 0.80 |
| TPA | 303.5 | 0.528 | 1.84 (1.20, 2.88) | 2.7 | 0.006 | 0.22 |  |
| CD8A | 303.8 | 0.527 | 0.55 (0.35, 0.84) | -2.7 | 0.007 | 0.22 | 0.73 |
| GRN | 303.8 | 0.527 | 3.61 (1.44, 9.57) | 2.7 | 0.008 | 0.22 |  |
| ADAMTS13 | 304.3 | 0.526 | 5.62 (1.57, 21.69) | 2.6 | 0.010 | 0.23 |  |
| PCSK9 | 304.5 | 0.525 | 3.24 (1.35, 8.09) | 2.6 | 0.010 | 0.23 |  |
| TNFRSF9 | 304.7 | 0.525 | 0.45 (0.24, 0.82) | -2.5 | 0.012 | 0.25 |  |
| CCL23 | 305.6 | 0.522 | 0.44 (0.22, 0.86) | -2.4 | 0.017 | 0.35 |  |
| MCP1 | 305.4 | 0.523 | 2.69 (1.20, 6.32) | 2.3 | 0.020 | 0.36 |  |
| GIF | 305.7 | 0.522 | 1.37 (1.06, 1.79) | 2.3 | 0.021 | 0.36 |  |
| IGFBP2 | 306.2 | 0.521 | 0.53 (0.30, 0.91) | -2.3 | 0.024 | 0.40 |  |
| OPN | 306.3 | 0.521 | 0.50 (0.27, 0.91) | -2.2 | 0.025 | 0.40 |  |
| CST5 | 306.7 | 0.519 | 0.54 (0.30, 0.94) | -2.1 | 0.033 | 0.49 | 0.41 |
| TLT2 | 306.8 | 0.519 | 0.51 (0.27, 0.94) | -2.1 | 0.034 | 0.49 |  |
| CASP3 | 307.0 | 0.519 | 1.24 (1.02, 1.53) | 2.1 | 0.038 | 0.51 |  |
| HSP27 | 307.2 | 0.518 | 1.95 (1.03, 3.79) | 2.0 | 0.043 | 0.51 |  |
| PECAM1 | 307.3 | 0.518 | 1.47 (1.01, 2.15) | 2.0 | 0.044 | 0.51 |  |
| MMP9 | 307.3 | 0.518 | 0.37 (0.14, 0.97) | -2.0 | 0.048 | 0.51 |  |
| MMP1 | 307.5 | 0.517 | 0.76 (0.58, 1.00) | -2.0 | 0.050 | 0.51 | 0.35 |
| SRC | 307.5 | 0.517 | 1.34 (1.00, 1.81) | 1.9 | 0.051 | 0.51 |  |
| MMP12 | 307.5 | 0.517 | 1.54 (1.01, 2.40) | 1.9 | 0.051 | 0.51 |  |
| FLT3L | 307.5 | 0.517 | 2.05 (1.00, 4.30) | 1.9 | 0.052 | 0.51 |  |
| PSPD | 307.5 | 0.517 | 1.44 (1.00, 2.11) | 1.9 | 0.053 | 0.51 |  |
| PDL1 | 307.8 | 0.517 | 0.53 (0.27, 1.02) | -1.9 | 0.058 | 0.51 |  |
| LTBR | 307.7 | 0.517 | 0.41 (0.16, 1.02) | -1.9 | 0.059 | 0.51 |  |
| CITP (Log) | 307.8 | 0.517 | 0.58 (0.33, 1.01) | -1.9 | 0.061 | 0.51 |  |
| JAMA | 307.9 | 0.516 | 1.28 (0.99, 1.68) | 1.9 | 0.063 | 0.51 |  |
| PI3 | 307.9 | 0.516 | 0.62 (0.38, 1.02) | -1.9 | 0.064 | 0.51 |  |
| CSF1 | 307.9 | 0.516 | 0.29 (0.08, 1.06) | -1.8 | 0.066 | 0.52 |  |
| IL2RA | 307.6 | 0.517 | 0.58 (0.30, 1.00) | -1.8 | 0.077 | 0.57 |  |
| MB | 308.3 | 0.515 | 0.62 (0.36, 1.06) | -1.7 | 0.081 | 0.57 |  |
| BLMHYDROLASE | 308.3 | 0.515 | 1.84(0.94, 3.70) | 1.7 | 0.083 | 0.57 |  |
| MPO | 307.7 | 0.517 | 3.95 (0.97, 20.87) | 1.7 | 0.083 | 0.57 |  |
| CTSD | 308.4 | 0.515 | 1.79 (0.93, 3.52) | 1.7 | 0.085 | 0.57 |  |
| PICP (Log) | 308.4 | 0.515 | 0.54 (0.26, 1.09) | -1.7 | 0.086 | 0.57 |  |
| IL10 | 308.4 | 0.515 | 1.38 (0.96, 2.02) | 1.7 | 0.088 | 0.57 |  |
| SELP | 308.5 | 0.515 | 1.35 (0.96, 1.93) | 1.7 | 0.092 | 0.57 |  |
| HAOX1 | 308.6 | 0.514 | 1.22 (0.97, 1.56) | 1.7 | 0.094 | 0.57 |  |
| GAL9 | 308.7 | 0.514 | 0.44 (0.16, 1.17) | -1.6 | 0.10 | 0.57 |  |
| TM | 308.7 | 0.514 | 0.44 (0.16, 1.16) | -1.6 | 0.10 | 0.57 |  |
| UPAR | 308.7 | 0.514 | 0.49 (0.20, 1.14) | -1.6 | 0.10 | 0.57 |  |
| IL18BP | 308.7 | 0.514 | 0.49 (0.21, 1.15) | -1.6 | 0.11 | 0.57 |  |
| FGF5 | 308.7 | 0.514 | 5.49 (0.72, 46.35) | 1.6 | 0.11 | 0.57 |  |
| MMP10 | 308.8 | 0.514 | 0.64 (0.37, 1.09) | -1.6 | 0.11 | 0.57 |  |
| NEMO | 308.8 | 0.514 | 1.20 (0.96, 1.50) | 1.6 | 0.11 | 0.57 |  |
| PRELP | 308.9 | 0.514 | 2.95 (0.78, 11.52) | 1.6 | 0.11 | 0.57 |  |
| IL7 | 308.9 | 0.514 | 1.34 (0.93, 1.93) | 1.6 | 0.11 | 0.57 |  |
| GDNF | 308.9 | 0.514 | 1.85 (0.87, 4.03) | 1.6 | 0.11 | 0.57 |  |
| EPCAM | 308.9 | 0.514 | 1.31 (0.94, 1.83) | 1.6 | 0.11 | 0.57 |  |
| TIE2 | 309.0 | 0.513 | 0.45 (0.16, 1.23) | -1.6 | 0.12 | 0.57 |  |
| UPA | 309.2 | 0.513 | 1.52 (0.87, 2.61) | 1.5 | 0.12 | 0.57 |  |
| IL24 | 308.4 | 0.515 | 2.05 (0.92, 5.77) | 1.5 | 0.12 | 0.57 |  |
| TRAILR2 | 309.0 | 0.513 | 0.52 (0.23, 1.18) | -1.5 | 0.12 | 0.57 |  |
| GAL3 (Log) | 309.0 | 0.513 | 0.51 (0.21, 1.20) | -1.5 | 0.12 | 0.57 |  |
| OPG | 309.0 | 0.513 | 2.08 (0.82, 5.46) | 1.5 | 0.13 | 0.57 |  |
| CCL28 | 309.1 | 0.513 | 0.49 (0.20, 1.22) | -1.5 | 0.13 | 0.57 |  |
| TNFR2 | 309.1 | 0.513 | 0.59 (0.29, 1.16) | -1.5 | 0.13 | 0.57 |  |
| CXCL5 | 309.2 | 0.513 | 1.14 (0.96, 1.36) | 1.5 | 0.14 | 0.58 |  |
| CD5 | 309.2 | 0.513 | 0.58 (0.28, 1.19) | -1.5 | 0.14 | 0.58 |  |
| TNFR1 | 309.2 | 0.513 | 0.53 (0.23, 1.22) | -1.5 | 0.14 | 0.58 |  |
| IL8 | 309.3 | 0.513 | 1.40 (0.88, 2.24) | 1.4 | 0.15 | 0.58 |  |
| hOSCAR | 309.3 | 0.513 | 0.44 (0.14, 1.33) | -1.4 | 0.15 | 0.59 |  |
| FABP2 | 309.3 | 0.512 | 1.33 (0.90, 1.99) | 1.4 | 0.15 | 0.59 |  |
| PARP1 | 309.2 | 0.513 | 0.77 (0.51, 1.09) | -1.4 | 0.16 | 0.59 |  |
| GDF2 | 309.4 | 0.512 | 1.73 (0.82, 3.76) | 1.4 | 0.16 | 0.59 |  |
| STK4 | 309.4 | 0.512 | 1.19 (0.93, 1.52) | 1.4 | 0.16 | 0.59 |  |
| CEACAM8 | 309.5 | 0.512 | 0.72 (0.45, 1.15) | -1.4 | 0.17 | 0.60 |  |
| SELE | 309.5 | 0.512 | 1.43 (0.87, 2.38) | 1.4 | 0.17 | 0.61 |  |
| TNFRSF10A | 309.5 | 0.512 | 1.81 (0.77, 4.30) | 1.4 | 0.17 | 0.61 |  |
| IL5 | 309.5 | 0.512 | 0.81 (0.59, 1.09) | -1.4 | 0.17 | 0.61 |  |
| IGFBP7 | 309.5 | 0.512 | 1.84 (0.77, 4.56) | 1.3 | 0.18 | 0.61 |  |
| IL6RA | 309.5 | 0.512 | 0.58 (0.26, 1.26) | -1.3 | 0.18 | 0.61 |  |
| TGM2 | 309.5 | 0.512 | 0.79 (0.54, 1.11) | -1.3 | 0.18 | 0.61 |  |
| PRTN3 | 309.3 | 0.512 | 2.92 (0.70, 17.38) | 1.3 | 0.19 | 0.62 |  |
| PTX3 | 309.7 | 0.512 | 0.64 (0.32, 1.24) | -1.3 | 0.19 | 0.63 |  |
| IL16 | 309.7 | 0.512 | 0.69 (0.38, 1.21) | -1.3 | 0.20 | 0.64 |  |
| VEGFA | 309.7 | 0.511 | 0.59 (0.26, 1.31) | -1.3 | 0.20 | 0.64 |  |
| TNFRSF13B | 309.7 | 0.511 | 0.68 (0.37, 1.21) | -1.3 | 0.20 | 0.64 |  |
| EGFR | 309.8 | 0.511 | 2.26 (0.64, 8.11) | 1.3 | 0.21 | 0.64 |  |
| LOX1 | 309.8 | 0.511 | 0.73 (0.45, 1.19) | -1.3 | 0.21 | 0.64 |  |
| CXCL6 | 309.9 | 0.511 | 1.23 (0.89, 1.71) | 1.3 | 0.21 | 0.64 |  |
| ITGB1BP2 | 309.8 | 0.511 | 1.14 (0.93, 1.41) | 1.3 | 0.21 | 0.64 |  |
| IL18 | 309.8 | 0.511 | 0.70 (0.40, 1.22) | -1.2 | 0.21 | 0.64 |  |
| HGF | 309.8 | 0.511 | 0.66 (0.33, 1.25) | -1.2 | 0.21 | 0.64 |  |
| TNFRSF10C | 309.9 | 0.511 | 0.67 (0.35, 1.26) | -1.2 | 0.22 | 0.64 |  |
| XCL1 | 309.9 | 0.511 | 0.73 (0.44, 1.21) | -1.2 | 0.22 | 0.65 |  |
| AXL | 310.0 | 0.511 | 0.58 (0.24, 1.40) | -1.2 | 0.23 | 0.65 |  |
| EPHB4 | 310.0 | 0.511 | 0.58 (0.23, 1.42) | -1.2 | 0.23 | 0.68 |  |
| IL12B | 310.1 | 0.511 | 0.78 (0.51, 1.19) | -1.2 | 0.24 | 0.68 |  |
| TFF3 | 310.0 | 0.511 | 0.65 (0.31, 1.33) | -1.2 | 0.25 | 0.69 |  |
| NT3 | 310.1 | 0.511 | 1.37 (0.81, 2.38) | 1.1 | 0.25 | 0.70 |  |
| TNFRSF11A | 310.1 | 0.510 | 0.66 (0.32, 1.34) | -1.1 | 0.26 | 0.70 |  |
| PSGL1 | 310.1 | 0.510 | 1.75 (0.67, 4.71) | 1.1 | 0.26 | 0.70 |  |
| CA5A | 310.1 | 0.510 | 0.82 (0.57, 1.16) | -1.1 | 0.26 | 0.70 |  |
| TNF | 308.8 | 0.514 | 0.41 (0.07, 1.13) | -1.1 | 0.26 | 0.70 |  |
| CTRC | 310.2 | 0.510 | 0.84 (0.61, 1.16) | -1.1 | 0.27 | 0.71 |  |
| MMP2 | 310.2 | 0.510 | 1.65 (0.68, 4.15) | 1.1 | 0.28 | 0.72 |  |
| CX3CL1 | 310.2 | 0.510 | 0.69 (0.35, 1.34) | -1.1 | 0.28 | 0.72 |  |
| SERPINA12 | 310.3 | 0.510 | 0.81 (0.55, 1.20) | -1.1 | 0.28 | 0.72 |  |
| PGF | 310.3 | 0.510 | 0.60 (0.23, 1.52) | -1.1 | 0.28 | 0.73 |  |
| VWF | 310.3 | 0.510 | 1.14 (0.90, 1.45) | 1.0 | 0.29 | 0.73 |  |
| AZU1 | 310.2 | 0.510 | 1.34 (0.81, 2.45) | 1.0 | 0.30 | 0.73 |  |
| HBEGF | 310.3 | 0.510 | 0.81 (0.53, 1.21) | -1.0 | 0.30 | 0.73 |  |
| IgGFcreceptorIlb | 310.3 | 0.510 | 1.21 (0.84, 1.76) | 1.0 | 0.30 | 0.73 |  |
| TRAIL | 310.3 | 0.510 | 0.57 (0.19, 1.65) | -1.0 | 0.30 | 0.73 |  |
| CCL20 | 310.4 | 0.510 | 0.86 (0.65, 1.14) | -1.0 | 0.30 | 0.73 |  |
| HSTNT (Log) | 310.4 | 0.510 | 0.71 (0.37, 1.36) | -1.0 | 0.31 | 0.73 |  |
| SCGB3A2 | 310.4 | 0.510 | 0.81 (0.53, 1.21) | -1.0 | 0.31 | 0.73 |  |
| VEGFD | 310.4 | 0.510 | 0.68 (0.31, 1.45) | -1.0 | 0.32 | 0.73 |  |
| TF | 310.4 | 0.510 | 0.63 (0.25, 1.56) | -1.0 | 0.32 | 0.74 |  |
| TNFSF13B | 310.5 | 0.510 | 1.44 (0.69, 3.07) | 1.0 | 0.33 | 0.75 |  |
| ADA | 310.5 | 0.509 | 1.39 (0.71, 2.72) | 1.0 | 0.34 | 0.77 |  |
| SOD2 | 310.5 | 0.509 | 2.85 (0.32, 24.94) | 0.9 | 0.34 | 0.77 |  |
| AXIN1 | 310.5 | 0.509 | 1.10 (0.90, 1.36) | 0.9 | 0.35 | 0.77 |  |
| RETN | 310.5 | 0.509 | 0.75 (0.40, 1.37) | -0.9 | 0.35 | 0.77 |  |
| cCXCL1 | 310.5 | 0.509 | 1.11 (0.89, 1.38) | 0.9 | 0.35 | 0.77 |  |
| SLAMF1 | 310.5 | 0.509 | 0.79 (0.48, 1.29) | -0.9 | 0.35 | 0.77 |  |
| IL10RB | 310.5 | 0.509 | 0.65 (0.26, 1.60) | -0.9 | 0.35 | 0.77 |  |
| PDGFsubunitA | 310.5 | 0.509 | 1.14 (0.86, 1.52) | 0.9 | 0.35 | 0.77 |  |
| SHPS1 | 310.6 | 0.509 | 1.41 (0.67, 2.98) | 0.9 | 0.37 | 0.77 |  |
| TR | 310.6 | 0.509 | 0.81 (0.51, 1.28) | -0.9 | 0.37 | 0.79 |  |
| CHI3L1 | 310.6 | 0.509 | 0.86 (0.62, 1.20) | -0.9 | 0.37 | 0.79 |  |
| CNTN1 | 310.6 | 0.509 | 1.50 (0.61, 3.72) | 0.9 | 0.37 | 0.79 |  |
| CCL24 | 310.6 | 0.509 | 1.17 (0.83, 1.65) | 0.9 | 0.38 | 0.79 |  |
| CTSL1 | 310.6 | 0.509 | 0.68 (0.29, 1.59) | -0.9 | 0.38 | 0.79 |  |
| AMBP | 310.7 | 0.509 | 0.50 (0.10, 2.35) | -0.9 | 0.39 | 0.79 |  |
| ALCAM | 310.7 | 0.509 | 1.66 (0.52, 5.38) | 0.9 | 0.40 | 0.80 |  |
| MEPE | 310.7 | 0.509 | 0.72 (0.33, 1.53) | -0.8 | 0.40 | 0.81 |  |
| BMP6 | 310.7 | 0.509 | 0.79 (0.44, 1.37) | -0.8 | 0.40 | 0.81 |  |
| CPA1 | 310.7 | 0.509 | 1.16 (0.82, 1.67) | 0.8 | 0.41 | 0.81 |  |
| GH | 310.7 | 0.509 | 1.08 (0.90, 1.29) | 0.8 | 0.41 | 0.81 |  |
| ADM | 310.7 | 0.509 | 0.70 (0.29, 1.64) | -0.8 | 0.41 | 0.81 |  |
| IL18R1 | 310.7 | 0.509 | 1.36 (0.66, 2.85) | 0.8 | 0.41 | 0.81 |  |
| PON3 | 310.8 | 0.509 | 0.79 (0.44, 1.40) | -0.8 | 0.42 | 0.81 |  |
| IDUA | 310.8 | 0.509 | 1.32 (0.66, 2.65) | 0.8 | 0.43 | 0.82 |  |
| IL20RA | 310.7 | 0.509 | 0.79 (0.40, 1.36) | -0.8 | 0.43 | 0.82 |  |
| BOC | 310.8 | 0.509 | 0.66 (0.23, 1.89) | -0.8 | 0.44 | 0.82 |  |
| FAS | 310.8 | 0.509 | 1.28 (0.66, 2.42) | 0.8 | 0.44 | 0.82 |  |
| TNFSF14 | 310.8 | 0.509 | 0.83 (0.52, 1.34) | -0.8 | 0.44 | 0.82 |  |
| SCF | 310.8 | 0.509 | 1.39 (0.60, 3.25) | 0.8 | 0.44 | 0.82 |  |
| STAMBP | 310.8 | 0.509 | 1.11 (0.85, 1.46) | 0.8 | 0.45 | 0.82 |  |
| GLO1 | 310.8 | 0.509 | 1.15 (0.80, 1.68) | 0.8 | 0.45 | 0.82 |  |
| THPO | 310.8 | 0.509 | 1.38 (0.59, 3.27) | 0.7 | 0.45 | 0.82 |  |
| NETANGF | 310.8 | 0.509 | 0.73 (0.29, 1.63) | -0.7 | 0.45 | 0.82 |  |
| CXCL9 | 310.9 | 0.509 | 0.86 (0.58, 1.27) | -0.7 | 0.46 | 0.82 |  |
| SIRT2 | 310.9 | 0.509 | 1.08 (0.88, 1.33) | 0.7 | 0.46 | 0.82 |  |
| IL1RT1 | 310.9 | 0.508 | 0.71 (0.28, 1.78) | -0.7 | 0.47 | 0.82 |  |
| PRSS8 | 310.9 | 0.508 | 1.39 (0.57, 3.46) | 0.7 | 0.47 | 0.82 |  |
| CD84 | 310.9 | 0.508 | 0.81 (0.45, 1.44) | -0.7 | 0.48 | 0.82 |  |
| LEP | 310.9 | 0.508 | 1.15 (0.79, 1.71) | 0.7 | 0.48 | 0.82 |  |
| FGF23 | 310.9 | 0.508 | 1.18 (0.74, 1.84) | 0.7 | 0.48 | 0.82 |  |
| MMP3 | 310.9 | 0.508 | 0.84 (0.52, 1.35) | -0.7 | 0.49 | 0.82 |  |
| IL13 | 311.0 | 0.508 | 0.89 (0.65, 1.26) | -0.7 | 0.49 | 0.82 |  |
| SLAMF7 | 310.9 | 0.508 | 0.87 (0.57, 1.31) | -0.7 | 0.49 | 0.82 |  |
| CTSZ | 310.9 | 0.508 | 0.76 (0.34, 1.68) | -0.7 | 0.50 | 0.82 |  |
| IL2RB | 310.9 | 0.508 | 1.29 (0.64, 2.89) | 0.7 | 0.50 | 0.82 |  |
| HO1 | 311.0 | 0.508 | 0.79 (0.39, 1.57) | -0.7 | 0.50 | 0.82 |  |
| COL1A1 | 311.0 | 0.508 | 0.77 (0.35, 1.67) | -0.7 | 0.50 | 0.82 |  |
| APR1 | 311.0 | 0.508 | 0.81 (0.43, 1.51) | -0.7 | 0.51 | 0.82 |  |
| PIGR | 311.0 | 0.508 | 1.77 (0.33, 9.57) | 0.7 | 0.51 | 0.82 |  |
| DLK1 | 311.0 | 0.508 | 1.20 (0.70, 2.09) | 0.7 | 0.51 | 0.82 |  |
| CCL3 | 311.0 | 0.508 | 0.87 (0.57, 1.28) | -0.7 | 0.51 | 0.82 |  |
| CD6 | 311.0 | 0.508 | 1.20 (0.69, 2.11) | 0.7 | 0.51 | 0.82 |  |
| IL27 | 311.0 | 0.508 | 1.28 (0.60, 2.75) | 0.6 | 0.52 | 0.82 |  |
| CSTB | 311.0 | 0.508 | 0.83 (0.45, 1.49) | -0.6 | 0.52 | 0.82 |  |
| IL10RA | 311.0 | 0.508 | 1.13 (0.77, 1.66) | 0.6 | 0.52 | 0.82 |  |
| TWEAK | 311.0 | 0.508 | 1.30 (0.57, 3.00) | 0.6 | 0.53 | 0.82 |  |
| IGFBP1 | 311.0 | 0.508 | 0.91 (0.68, 1.22) | -0.6 | 0.53 | 0.82 |  |
| DECR1 | 311.0 | 0.508 | 1.06 (0.87, 1.30) | 0.6 | 0.54 | 0.82 |  |
| CD40 | 311.0 | 0.508 | 0.82 (0.42, 1.57) | -0.6 | 0.54 | 0.84 |  |
| ITGB2 | 311.0 | 0.508 | 0.79 (0.37, 1.67) | -0.6 | 0.55 | 0.84 |  |
| CDCP1 | 311.1 | 0.508 | 0.87 (0.53, 1.39) | -0.6 | 0.55 | 0.84 |  |
| MCP2 | 311.1 | 0.508 | 0.87 (0.55, 1.37) | -0.6 | 0.56 | 0.84 |  |
| TIMP4 | 311.1 | 0.508 | 0.83 (0.45, 1.55) | -0.6 | 0.57 | 0.84 |  |
| CPB1 | 311.1 | 0.508 | 1.12 (0.77, 1.66) | 0.6 | 0.57 | 0.85 |  |
| GT | 311.1 | 0.508 | 1.13 (0.74, 1.73) | 0.6 | 0.57 | 0.85 |  |
| ST1A1 | 311.1 | 0.508 | 1.10 (0.79, 1.54) | 0.5 | 0.58 | 0.86 |  |
| IL17D | 311.1 | 0.508 | 1.18 (0.61, 2.17) | 0.5 | 0.59 | 0.86 |  |
| NTproBNP (Log) | 311.1 | 0.508 | 0.87 (0.52, 1.45) | -0.5 | 0.59 | 0.86 |  |
| AGRP | 311.1 | 0.508 | 0.85 (0.47, 1.56) | -0.5 | 0.60 | 0.86 |  |
| ACE2 | 311.2 | 0.508 | 1.13 (0.70, 1.82) | 0.5 | 0.61 | 0.86 |  |
| CD40L | 311.2 | 0.508 | 1.05 (0.86, 1.31) | 0.5 | 0.62 | 0.88 |  |
| REN | 311.2 | 0.508 | 1.09 (0.78, 1.50) | 0.5 | 0.62 | 0.89 |  |
| IL1RT2 | 311.2 | 0.508 | 1.24 (0.51, 3.05) | 0.5 | 0.63 | 0.89 |  |
| CCL25 | 311.2 | 0.508 | 0.87 (0.50, 1.53) | -0.5 | 0.63 | 0.89 |  |
| IL22RA1 | 311.2 | 0.508 | 0.77 (0.23, 2.11) | -0.5 | 0.64 | 0.89 |  |
| MCP3 | 311.2 | 0.508 | 0.89 (0.52, 1.45) | -0.5 | 0.64 | 0.89 |  |
| LDLRECEPTOR | 311.2 | 0.508 | 1.15 (0.63, 2.13) | 0.5 | 0.65 | 0.89 |  |
| IL4 | 311.2 | 0.508 | 0.88 (0.51, 1.49) | -0.4 | 0.65 | 0.89 |  |
| MERTK | 311.2 | 0.508 | 1.20 (0.55, 2.62) | 0.4 | 0.65 | 0.89 |  |
| TSLP | 311.2 | 0.508 | 0.84 (0.38, 1.83) | -0.4 | 0.66 | 0.89 |  |
| KIM1 | 311.2 | 0.508 | 0.91 (0.61, 1.37) | -0.4 | 0.66 | 0.89 |  |
| IL33 | 311.2 | 0.508 | 2.95 (0.04, 1.25E+3) | 0.4 | 0.66 | 0.89 |  |
| CASP8 | 311.2 | 0.507 | 0.90 (0.55, 1.46) | -0.4 | 0.68 | 0.89 |  |
| ANG1 | 311.2 | 0.507 | 0.95 (0.74, 1.22) | -0.4 | 0.68 | 0.90 |  |
| MARCO | 311.2 | 0.507 | 0.76 (0.21, 2.83) | -0.4 | 0.69 | 0.90 |  |
| CXCL16 | 311.3 | 0.507 | 1.24 (0.42, 3.62) | 0.4 | 0.69 | 0.90 |  |
| IL15RA | 311.3 | 0.507 | 0.82 (0.29, 2.26) | -0.4 | 0.70 | 0.91 |  |
| CCL19 | 311.3 | 0.507 | 1.07 (0.77, 1.50) | 0.4 | 0.70 | 0.92 |  |
| CD244 | 311.3 | 0.507 | 0.88 (0.43, 1.73) | -0.4 | 0.71 | 0.92 |  |
| PAPPA | 311.3 | 0.507 | 0.90 (0.52, 1.56) | -0.4 | 0.71 | 0.92 |  |
| NRTN | 311.3 | 0.507 | 1.15 (0.58, 2.75) | 0.4 | 0.71 | 0.92 |  |
| CXCL11 | 311.3 | 0.507 | 1.06 (0.76, 1.47) | 0.4 | 0.73 | 0.92 |  |
| IL1RL2 | 311.3 | 0.507 | 1.14 (0.55, 2.35) | 0.3 | 0.73 | 0.92 |  |
| IL17C | 311.3 | 0.507 | 0.93 (0.60, 1.44) | -0.3 | 0.75 | 0.93 |  |
| RAGE | 311.3 | 0.507 | 1.12 (0.54, 2.33) | 0.3 | 0.75 | 0.93 |  |
| ENRAGE | 311.3 | 0.507 | 0.95 (0.66, 1.35) | -0.3 | 0.76 | 0.93 |  |
| CCL17 | 311.3 | 0.507 | 1.04 (0.79, 1.39) | 0.3 | 0.76 | 0.93 |  |
| MCP4 | 311.3 | 0.507 | 0.93 (0.60, 1.45) | -0.3 | 0.76 | 0.93 |  |
| IFNGAMMA | 311.3 | 0.507 | 1.72 (0.01, 22.16) | 0.3 | 0.76 | 0.93 |  |
| CD93 | 311.3 | 0.507 | 0.86 (0.32, 2.29) | -0.3 | 0.77 | 0.93 |  |
| ST2 | 311.3 | 0.507 | 1.09 (0.62, 1.91) | 0.3 | 0.77 | 0.93 |  |
| PLC | 311.3 | 0.507 | 0.84 (0.27, 2.65) | -0.3 | 0.77 | 0.93 |  |
| PRSS27 | 311.3 | 0.507 | 0.91 (0.49, 1.68) | -0.3 | 0.77 | 0.93 |  |
| PIIINP (Log) | 311.3 | 0.507 | 0.93 (0.56, 1.54) | -0.3 | 0.77 | 0.93 |  |
| CCL11 | 311.3 | 0.507 | 1.10 (0.57, 2.12) | 0.3 | 0.77 | 0.93 |  |
| DNER | 311.3 | 0.507 | 1.16 (0.43, 3.12) | 0.3 | 0.78 | 0.93 |  |
| THBS2 | 311.3 | 0.507 | 1.18 (0.36, 3.85) | 0.3 | 0.78 | 0.93 |  |
| RARRES2 | 311.3 | 0.507 | 1.15 (0.41, 3.26) | 0.3 | 0.78 | 0.93 |  |
| CHIT1 | 311.3 | 0.507 | 1.04 (0.80, 1.35) | 0.3 | 0.79 | 0.93 |  |
| TNFB | 311.3 | 0.507 | 1.09 (0.57, 2.07) | 0.3 | 0.79 | 0.93 |  |
| CCL4 | 311.3 | 0.507 | 0.95 (0.64, 1.41) | -0.3 | 0.79 | 0.93 |  |
| PDGFsubunitB | 311.3 | 0.507 | 0.97 (0.79, 1.20) | -0.3 | 0.79 | 0.93 |  |
| IL4RA | 311.3 | 0.507 | 1.11 (0.48, 2.60) | 0.2 | 0.80 | 0.93 |  |
| CCL15 | 311.3 | 0.507 | 1.09 (0.55, 2.17) | 0.2 | 0.80 | 0.93 |  |
| BetaNGF | 311.4 | 0.507 | 0.89 (0.34, 2.28) | -0.2 | 0.81 | 0.93 |  |
| CCL16 | 311.4 | 0.507 | 0.94 (0.58, 1.53) | -0.2 | 0.81 | 0.93 |  |
| IL1alpha | 311.4 | 0.507 | 0.77 (0.04, 4.73) | -0.2 | 0.81 | 0.93 |  |
| VSIG2 | 311.4 | 0.507 | 1.06 (0.66, 1.69) | 0.2 | 0.81 | 0.93 |  |
| ARTN | 311.4 | 0.507 | 0.93 (0.48, 1.77) | -0.2 | 0.82 | 0.93 |  |
| GP6 | 311.4 | 0.507 | 1.04 (0.74, 1.47) | 0.2 | 0.82 | 0.93 |  |
| LAPTGFbeta1 | 311.4 | 0.507 | 0.93 (0.49, 1.76) | -0.2 | 0.83 | 0.93 |  |
| GDF15 (Log) | 311.4 | 0.507 | 0.93 (0.47, 1.82) | -0.2 | 0.83 | 0.93 |  |
| TNFRSF14 | 311.4 | 0.507 | 0.93 (0.48, 1.83) | -0.2 | 0.83 | 0.93 |  |
| CD4 | 311.4 | 0.507 | 0.89 (0.31, 2.56) | -0.2 | 0.83 | 0.93 |  |
| DCN | 311.4 | 0.507 | 1.10 (0.46, 2.64) | 0.2 | 0.84 | 0.93 |  |
| SPON1 | 311.4 | 0.507 | 1.10 (0.44, 2.77) | 0.2 | 0.84 | 0.93 |  |
| FS | 311.4 | 0.507 | 1.07 (0.53, 2.18) | 0.2 | 0.85 | 0.94 |  |
| ICAM2 | 311.4 | 0.507 | 0.94 (0.46, 1.94) | -0.2 | 0.86 | 0.94 |  |
| TRAP | 311.4 | 0.507 | 0.93 (0.40, 2.15) | -0.2 | 0.87 | 0.94 |  |
| SORT1 | 311.4 | 0.507 | 1.07 (0.42, 2.73) | 0.1 | 0.88 | 0.96 |  |
| IL6 | 311.4 | 0.507 | 0.98 (0.70, 1.36) | -0.1 | 0.89 | 0.96 |  |
| BP14E | 311.4 | 0.507 | 1.03 (0.72, 1.46) | 0.1 | 0.89 | 0.96 |  |
| IL17A | 311.4 | 0.507 | 1.03 (0.71, 1.48) | 0.1 | 0.89 | 0.96 |  |
| LPL | 311.4 | 0.507 | 0.96 (0.53, 1.72) | -0.1 | 0.89 | 0.96 |  |
| IL1RA | 311.4 | 0.507 | 0.96 (0.56, 1.66) | -0.1 | 0.89 | 0.96 |  |
| TFPI | 311.4 | 0.507 | 0.95 (0.46, 1.95) | -0.1 | 0.90 | 0.96 |  |
| LIF | 311.4 | 0.507 | 1.05 (0.47, 2.46) | 0.1 | 0.90 | 0.96 |  |
| KLK6 | 311.4 | 0.507 | 0.96 (0.49, 1.90) | -0.1 | 0.91 | 0.96 |  |
| FGF19 | 311.4 | 0.507 | 1.02 (0.73, 1.41) | 0.1 | 0.92 | 0.96 |  |
| IL17RA | 311.4 | 0.507 | 1.03 (0.62, 1.70) | 0.1 | 0.92 | 0.96 |  |
| CDH5 | 311.4 | 0.507 | 1.04 (0.46, 2.35) | 0.1 | 0.92 | 0.96 |  |
| CD163 | 311.4 | 0.507 | 0.97 (0.51, 1.84) | -0.1 | 0.93 | 0.96 |  |
| PDL2 | 311.4 | 0.507 | 1.04 (0.42, 2.53) | 0.1 | 0.94 | 0.97 |  |
| APN | 311.4 | 0.507 | 0.96 (0.37, 2.48) | -0.1 | 0.94 | 0.97 |  |
| CXCL10 | 311.4 | 0.507 | 1.01 (0.70, 1.49) | 0.1 | 0.94 | 0.97 |  |
| FABP4 | 311.4 | 0.507 | 1.02 (0.64, 1.62) | 0.1 | 0.95 | 0.97 |  |
| NOTCH3 | 311.4 | 0.507 | 1.03 (0.44, 2.42) | 0.1 | 0.95 | 0.97 |  |
| FGF21 | 311.4 | 0.507 | 0.99 (0.79, 1.26) | 0.0 | 0.97 | 0.97 |  |
| DKK1 | 311.4 | 0.507 | 0.99 (0.64, 1.53) | 0.0 | 0.97 | 0.98 |  |
| IL20 | 311.4 | 0.507 | 1.01 (0.43, 2.01) | 0.0 | 0.99 | 0.98 |  |
| IL2 | 311.4 | 0.507 | 0.00 (.00 - 0.00) | 0.0 | 0.99 | 0.99 |  |
| SPON2 | 311.4 | 0.507 | 0.99 (0.20, 4.90) | 0.0 | 0.99 | 0.99 |  |

Supplemental table 5 - Proteins included in the logistic regression model, adjusted to the defined clinically relevant variables, comparing “MI” *versus* “no CAD/no MI” participants. Full names of proteins can be found in *supplemental table 1*.

| Protein | AIC | r2 | OR (95% CI) | Z | *p*-value | FDR adjusted *p*-value | Proportion of significant FDR adjusted p-values (bootstraped models) |
| --- | --- | --- | --- | --- | --- | --- | --- |
| MMP7 | 309.2 | 0.591 | 3.27 (1.85, 6.01) | 4.0 | <0.001 | 0.02 | 0.98 |
| NT3 | 313.7 | 0.581 | 3.06 (1.64, 5.98) | 3.4 | <0.001 | 0.09 | 0.96 |
| PSPD | 315.2 | 0.578 | 1.92 (1.31, 2.85) | 3.3 | 0.001 | 0.09 | 0.93 |
| CTSD | 316.3 | 0.576 | 3.04 (1.54, 6.19) | 3.1 | 0.002 | 0.12 |  |
| KIM1 | 317.1 | 0.574 | 1.82 (1.25, 2.70) | 3.1 | 0.002 | 0.13 |  |
| GRN | 317.4 | 0.573 | 4.21 (1.67, 11.03) | 3.0 | 0.003 | 0.13 |  |
| OPG | 317.9 | 0.572 | 4.03 (1.60, 10.57) | 2.9 | 0.004 | 0.13 |  |
| TRANCE | 317.9 | 0.572 | 0.48 (0.29, 0.78) | -2.9 | 0.004 | 0.13 | 0.83 |
| TPA | 320.9 | 0.566 | 1.58 (1.09, 2.34) | 2.4 | 0.018 | 0.42 |  |
| ADA | 321.1 | 0.565 | 2.21 (1.15, 4.35) | 2.3 | 0.019 | 0.42 |  |
| UPA | 321.9 | 0.563 | 2.00 (1.08, 3.58) | 2.3 | 0.020 | 0.42 |  |
| ACE2 | 321.8 | 0.564 | 1.68 (1.07, 2.69) | 2.2 | 0.027 | 0.42 |  |
| AGRP | 321.9 | 0.564 | 1.88 (1.08, 3.33) | 2.2 | 0.028 | 0.43 |  |
| IL8 | 321.8 | 0.564 | 1.63 (1.06, 2.56) | 2.2 | 0.028 | 0.43 |  |
| PCSK9 | 322.0 | 0.563 | 2.51 (1.10, 5.85) | 2.2 | 0.030 | 0.43 |  |
| CCL16 | 322.1 | 0.563 | 1.61 (1.04, 2.50) | 2.2 | 0.031 | 0.43 |  |
| VWF | 322.2 | 0.563 | 1.29 (1.02, 1.64) | 2.1 | 0.036 | 0.43 |  |
| NTproBNP (Log) | 322.4 | 0.562 | 1.59 (1.03, 2.49) | 2.1 | 0.040 | 0.43 | 0.52 |
| IL5 | 322.7 | 0.562 | 0.75 (0.57, 0.99) | -2.1 | 0.040 | 0.43 | 0.55 |
| HSTNT (Log) | 322.6 | 0.562 | 1.90 (1.03, 3.58) | 2.0 | 0.042 | 0.43 |  |
| HAOX1 | 322.6 | 0.562 | 1.27 (1.01, 1.60) | 2.0 | 0.042 | 0.43 |  |
| MMP12 | 322.5 | 0.562 | 1.58 (1.02, 2.49) | 2.0 | 0.042 | 0.43 |  |
| BLMHYDROLASE | 322.5 | 0.562 | 2.02 (1.04, 4.06) | 2.0 | 0.043 | 0.43 |  |
| GAL4 | 322.7 | 0.562 | 1.74 (1.02, 3.04) | 2.0 | 0.045 | 0.43 |  |
| CXCL6 | 322.6 | 0.562 | 1.36 (1.01, 1.86) | 2.0 | 0.046 | 0.43 |  |
| TNFRSF10A | 322.8 | 0.562 | 2.37 (1.02, 5.67) | 2.0 | 0.048 | 0.43 |  |
| IGFBP7 | 322.7 | 0.562 | 2.64 (1.03, 7.11) | 2.0 | 0.049 | 0.43 |  |
| PIGR | 323.2 | 0.561 | 5.54 (0.93, 35.89) | 1.8 | 0.065 | 0.43 |  |
| TRAIL | 323.4 | 0.560 | 0.41 (0.15, 1.06) | -1.8 | 0.070 | 0.43 |  |
| IL1RA | 323.5 | 0.560 | 1.58 (0.97, 2.64) | 1.8 | 0.072 | 0.56 |  |
| IDUA | 323.6 | 0.560 | 1.82 (0.95, 3.57) | 1.8 | 0.075 | 0.58 |  |
| PRSS8 | 323.6 | 0.560 | 2.24 (0.92, 5.58) | 1.8 | 0.077 | 0.58 |  |
| MCP1 | 323.6 | 0.560 | 1.89 (0.94, 3.94) | 1.7 | 0.081 | 0.59 |  |
| MMP2 | 323.7 | 0.560 | 2.23 (0.91, 5.68) | 1.7 | 0.085 | 0.59 |  |
| CST5 | 323.9 | 0.559 | 0.61 (0.34, 1.08) | -1.7 | 0.091 | 0.61 |  |
| ALCAM | 323.9 | 0.559 | 2.60 (0.86, 8.05) | 1.7 | 0.093 | 0.61 |  |
| EPCAM | 323.9 | 0.559 | 1.33 (0.96, 1.87) | 1.7 | 0.094 | 0.61 |  |
| TNFSF13B | 323.9 | 0.559 | 1.93 (0.90, 4.28) | 1.7 | 0.095 | 0.61 |  |
| TR | 324.0 | 0.559 | 0.70 (0.46, 1.07) | -1.6 | 0.10 | 0.61 |  |
| TIE2 | 324.2 | 0.558 | 0.43 (0.15, 1.21) | -1.6 | 0.11 | 0.61 |  |
| TGFALPHA | 324.3 | 0.558 | 0.46 (0.17, 1.21) | -1.6 | 0.12 | 0.63 |  |
| TNFRSF13B | 324.2 | 0.558 | 0.60 (0.31, 1.12) | -1.6 | 0.12 | 0.68 |  |
| IL24 | 323.1 | 0.561 | 1.90 (0.99, 4.89) | 1.6 | 0.12 | 0.68 |  |
| CASP3 | 324.3 | 0.558 | 1.17 (0.96, 1.43) | 1.5 | 0.12 | 0.68 |  |
| JAMA | 324.4 | 0.558 | 1.23 (0.95, 1.60) | 1.5 | 0.12 | 0.68 |  |
| PAI | 324.4 | 0.558 | 1.26 (0.94, 1.71) | 1.5 | 0.13 | 0.69 |  |
| FLT3L | 324.5 | 0.558 | 1.60 (0.87, 2.98) | 1.5 | 0.13 | 0.69 |  |
| HSP27 | 324.6 | 0.558 | 1.55 (0.87, 2.80) | 1.5 | 0.14 | 0.70 |  |
| PECAM1 | 324.6 | 0.558 | 1.31 (0.92, 1.90) | 1.5 | 0.14 | 0.71 |  |
| IL4RA | 324.6 | 0.558 | 1.81 (0.82, 4.05) | 1.5 | 0.14 | 0.72 |  |
| IL27 | 324.7 | 0.557 | 1.73 (0.83, 3.67) | 1.5 | 0.15 | 0.72 |  |
| IL12B | 324.7 | 0.557 | 0.75 (0.50, 1.11) | -1.4 | 0.15 | 0.72 |  |
| MB | 324.7 | 0.557 | 1.46 (0.87, 2.46) | 1.4 | 0.15 | 0.72 |  |
| GDF2 | 324.7 | 0.557 | 1.77 (0.82, 3.90) | 1.4 | 0.15 | 0.72 |  |
| SERPINA12 | 324.8 | 0.557 | 0.73 (0.46, 1.13) | -1.4 | 0.16 | 0.72 |  |
| SELP | 324.8 | 0.557 | 1.28 (0.91, 1.83) | 1.4 | 0.16 | 0.72 |  |
| GAL9 | 324.8 | 0.557 | 0.49 (0.18, 1.33) | -1.4 | 0.16 | 0.73 |  |
| PRELP | 324.9 | 0.557 | 2.46 (0.69, 9.02) | 1.4 | 0.17 | 0.73 |  |
| TLT2 | 324.9 | 0.557 | 0.65 (0.35, 1.20) | -1.4 | 0.17 | 0.73 |  |
| MPO | 324.3 | 0.558 | 3.15 (0.78, 20.27) | 1.4 | 0.17 | 0.73 |  |
| IL13 | 325.0 | 0.557 | 0.82 (0.62, 1.11) | -1.4 | 0.17 | 0.73 |  |
| ADAMTS13 | 324.9 | 0.557 | 2.45 (0.68, 9.13) | 1.4 | 0.18 | 0.73 |  |
| GDNF | 325.0 | 0.557 | 1.67 (0.79, 3.56) | 1.3 | 0.18 | 0.73 |  |
| GDF15 (Log) | 325.1 | 0.557 | 1.56 (0.80, 3.06) | 1.3 | 0.19 | 0.73 |  |
| OSM | 325.1 | 0.557 | 0.79 (0.55, 1.12) | -1.3 | 0.19 | 0.73 |  |
| SHPS1 | 325.1 | 0.557 | 1.56 (0.80, 3.10) | 1.3 | 0.20 | 0.76 |  |
| XCL1 | 325.2 | 0.556 | 0.72 (0.43, 1.19) | -1.3 | 0.21 | 0.78 |  |
| CTSZ | 325.2 | 0.556 | 1.66 (0.75, 3.68) | 1.3 | 0.21 | 0.78 |  |
| AZU1 | 324.8 | 0.557 | 1.49 (0.88, 3.07) | 1.2 | 0.21 | 0.79 |  |
| IL17D | 325.3 | 0.556 | 1.46 (0.78, 2.84) | 1.2 | 0.22 | 0.79 |  |
| CCL23 | 325.3 | 0.556 | 0.65 (0.32, 1.30) | -1.2 | 0.22 | 0.80 |  |
| TSLP | 325.1 | 0.556 | 0.50 (0.11, 1.43) | -1.2 | 0.23 | 0.80 |  |
| PDL1 | 325.4 | 0.556 | 0.66 (0.33, 1.31) | -1.2 | 0.23 | 0.81 |  |
| CITP (Log) | 325.3 | 0.556 | 0.72 (0.41, 1.23) | -1.2 | 0.23 | 0.82 |  |
| MCP4 | 325.4 | 0.556 | 1.27 (0.86, 1.91) | 1.2 | 0.24 | 0.82 |  |
| ITGB1BP2 | 325.4 | 0.556 | 1.13 (0.92, 1.39) | 1.2 | 0.24 | 0.82 |  |
| PGLYRP1 | 325.4 | 0.556 | 0.71 (0.39, 1.26) | -1.2 | 0.25 | 0.82 |  |
| TNFRSF9 | 325.5 | 0.556 | 0.71 (0.40, 1.27) | -1.1 | 0.26 | 0.83 |  |
| IL33 | 325.0 | 0.557 | 19.05 (0.41, 1.27E4) | 1.1 | 0.26 | 0.83 |  |
| PSGL1 | 325.5 | 0.556 | 1.74 (0.66, 4.63) | 1.1 | 0.26 | 0.83 |  |
| LDLRECEPTOR | 325.5 | 0.556 | 1.42 (0.77, 2.66) | 1.1 | 0.27 | 0.84 |  |
| SRC | 325.6 | 0.555 | 1.16 (0.89, 1.52) | 1.1 | 0.27 | 0.84 |  |
| FABP4 | 325.6 | 0.555 | 1.31 (0.80, 2.17) | 1.1 | 0.28 | 0.84 |  |
| CXCL9 | 325.6 | 0.555 | 0.79 (0.52, 1.21) | -1.1 | 0.28 | 0.84 |  |
| NEMO | 325.6 | 0.555 | 1.12 (0.91, 1.39) | 1.1 | 0.28 | 0.85 |  |
| CCL28 | 325.6 | 0.555 | 0.64 (0.28, 1.46) | -1.1 | 0.29 | 0.85 |  |
| ICAM2 | 325.7 | 0.555 | 0.68 (0.33, 1.39) | -1.1 | 0.29 | 0.85 |  |
| MMP1 | 325.7 | 0.555 | 0.87 (0.66, 1.13) | -1.1 | 0.29 | 0.85 |  |
| BP14E | 325.7 | 0.555 | 1.21 (0.85, 1.74) | 1.1 | 0.29 | 0.85 |  |
| FGF21 | 325.7 | 0.555 | 1.14 (0.89, 1.46) | 1.0 | 0.30 | 0.85 |  |
| CXCL11 | 325.7 | 0.555 | 1.17 (0.87, 1.60) | 1.0 | 0.30 | 0.85 |  |
| LIF | 325.7 | 0.555 | 0.56 (0.16, 1.72) | -1.0 | 0.31 | 0.85 |  |
| SPON2 | 325.8 | 0.555 | 2.17 (0.48, 9.78) | 1.0 | 0.31 | 0.85 |  |
| IL17RA | 325.8 | 0.555 | 1.29 (0.79, 2.14) | 1.0 | 0.31 | 0.85 |  |
| RARRES2 | 325.8 | 0.555 | 1.72 (0.59, 4.97) | 1.0 | 0.32 | 0.85 |  |
| PICP (Log) | 325.8 | 0.555 | 0.72 (0.37, 1.37) | -1.0 | 0.32 | 0.85 |  |
| CXCL16 | 325.8 | 0.555 | 1.72 (0.59, 5.05) | 1.0 | 0.32 | 0.85 |  |
| IL2RB | 325.8 | 0.555 | 1.42 (0.73, 2.98) | 1.0 | 0.32 | 0.85 |  |
| SPON1 | 325.8 | 0.555 | 1.60 (0.63, 4.12) | 1.0 | 0.33 | 0.85 |  |
| FGF19 | 325.8 | 0.555 | 1.17 (0.85, 1.62) | 1.0 | 0.33 | 0.85 |  |
| RAGE | 325.8 | 0.555 | 1.42 (0.70, 2.90) | 1.0 | 0.33 | 0.85 |  |
| EPHB4 | 325.9 | 0.555 | 0.64 (0.26, 1.58) | -1.0 | 0.34 | 0.85 |  |
| IgGFcreceptorIlb | 325.9 | 0.555 | 1.18 (0.83, 1.68) | 0.9 | 0.34 | 0.85 |  |
| FAS | 325.9 | 0.555 | 1.43 (0.68, 3.05) | 0.9 | 0.35 | 0.85 |  |
| STK4 | 325.9 | 0.555 | 1.12 (0.89, 1.41) | 0.9 | 0.35 | 0.86 |  |
| ARTN | 326.0 | 0.555 | 0.72 (0.35, 1.48) | -0.9 | 0.37 | 0.86 |  |
| PRTN3 | 325.7 | 0.555 | 1.93 (0.65, 11.98) | 0.9 | 0.37 | 0.86 |  |
| TNF | 325.8 | 0.555 | 0.71 (0.32, 1.35) | -0.9 | 0.37 | 0.87 |  |
| LTBR | 326.0 | 0.555 | 0.68 (0.29, 1.59) | -0.9 | 0.38 | 0.87 |  |
| TRAILR2 | 326.0 | 0.554 | 1.42 (0.64, 3.18) | 0.9 | 0.38 | 0.87 |  |
| GIF | 326.0 | 0.555 | 1.12 (0.87, 1.45) | 0.9 | 0.38 | 0.87 |  |
| hOSCAR | 326.0 | 0.554 | 0.60 (0.18, 1.90) | -0.9 | 0.38 | 0.87 |  |
| CD163 | 326.0 | 0.554 | 0.76 (0.41, 1.40) | -0.9 | 0.39 | 0.87 |  |
| OPN | 326.0 | 0.554 | 0.77 (0.43, 1.39) | -0.9 | 0.39 | 0.87 |  |
| CD93 | 326.0 | 0.554 | 1.51 (0.59, 3.90) | 0.9 | 0.39 | 0.87 |  |
| SIRT2 | 326.0 | 0.554 | 1.09 (0.89, 1.34) | 0.9 | 0.39 | 0.87 |  |
| CXCL5 | 326.1 | 0.554 | 1.07 (0.91, 1.27) | 0.9 | 0.39 | 0.87 |  |
| GT | 326.1 | 0.554 | 1.19 (0.79, 1.80) | 0.8 | 0.40 | 0.87 |  |
| CHI3L1 | 326.1 | 0.554 | 1.16 (0.83, 1.63) | 0.8 | 0.40 | 0.87 |  |
| CEACAM8 | 326.1 | 0.554 | 0.81 (0.50, 1.33) | -0.8 | 0.40 | 0.87 |  |
| AXIN1 | 326.1 | 0.554 | 1.09 (0.90, 1.33) | 0.8 | 0.40 | 0.87 |  |
| SLAMF7 | 326.1 | 0.554 | 0.82 (0.52, 1.29) | -0.8 | 0.41 | 0.87 |  |
| CXCL1 | 326.1 | 0.554 | 1.09 (0.89, 1.34) | 0.8 | 0.41 | 0.87 |  |
| STAMBP | 326.1 | 0.554 | 1.12 (0.86, 1.47) | 0.8 | 0.41 | 0.87 |  |
| FGF23 | 326.1 | 0.554 | 1.21 (0.76, 1.95) | 0.8 | 0.42 | 0.87 |  |
| PDGFsubunitB | 326.1 | 0.554 | 0.93 (0.76, 1.12) | -0.8 | 0.42 | 0.87 |  |
| CD84 | 326.1 | 0.554 | 0.79 (0.44, 1.41) | -0.8 | 0.42 | 0.87 |  |
| SORT1 | 326.2 | 0.554 | 1.45 (0.58, 3.71) | 0.8 | 0.43 | 0.87 |  |
| TGM2 | 326.2 | 0.554 | 1.14 (0.82, 1.61) | 0.8 | 0.43 | 0.87 |  |
| SOD2 | 326.2 | 0.554 | 2.34 (0.28, 19.45) | 0.8 | 0.43 | 0.87 |  |
| CHIT1 | 326.2 | 0.554 | 0.91 (0.71, 1.15) | -0.8 | 0.44 | 0.87 |  |
| TNFB | 326.2 | 0.554 | 0.79 (0.42, 1.43) | -0.8 | 0.44 | 0.87 |  |
| KLK6 | 326.2 | 0.554 | 1.29 (0.67, 2.51) | 0.8 | 0.45 | 0.87 |  |
| GAL3 (Log) | 326.2 | 0.554 | 0.75 (0.36, 1.58) | -0.8 | 0.45 | 0.87 |  |
| TFF3 | 326.2 | 0.554 | 0.76 (0.36, 1.55) | -0.8 | 0.45 | 0.87 |  |
| CASP8 | 326.2 | 0.554 | 1.20 (0.76, 1.94) | 0.7 | 0.45 | 0.87 |  |
| LAPTGFbeta1 | 326.2 | 0.554 | 1.25 (0.70, 2.29) | 0.7 | 0.46 | 0.87 |  |
| TM | 326.2 | 0.554 | 0.70 (0.28, 1.77) | -0.7 | 0.46 | 0.87 |  |
| MCP2 | 326.2 | 0.554 | 1.20 (0.75, 1.93) | 0.7 | 0.46 | 0.87 |  |
| TNFRSF14 | 326.3 | 0.554 | 1.29 (0.64, 2.66) | 0.7 | 0.47 | 0.87 |  |
| CCL15 | 326.3 | 0.554 | 1.27 (0.66, 2.49) | 0.7 | 0.48 | 0.87 |  |
| FGF5 | 326.3 | 0.554 | 1.95 (0.33, 13.90) | 0.7 | 0.48 | 0.87 |  |
| IL10 | 326.3 | 0.554 | 1.18 (0.73, 1.82) | 0.7 | 0.48 | 0.89 |  |
| FS | 326.3 | 0.554 | 1.27 (0.64, 2.55) | 0.7 | 0.49 | 0.89 |  |
| PI3 | 326.3 | 0.554 | 0.83 (0.48, 1.42) | -0.7 | 0.49 | 0.89 |  |
| CTRC | 326.3 | 0.554 | 0.89 (0.63, 1.25) | -0.7 | 0.50 | 0.89 |  |
| ANG1 | 326.3 | 0.554 | 0.93 (0.74, 1.16) | -0.7 | 0.50 | 0.90 |  |
| IL20 | 326.3 | 0.554 | 0.77 (0.31, 1.55) | -0.7 | 0.50 | 0.90 |  |
| TNFR2 | 326.3 | 0.554 | 0.80 (0.40, 1.56) | -0.7 | 0.51 | 0.90 |  |
| CPA1 | 326.3 | 0.554 | 1.13 (0.79, 1.63) | 0.7 | 0.51 | 0.90 |  |
| PON3 | 326.4 | 0.554 | 0.83 (0.48, 1.44) | -0.6 | 0.52 | 0.90 |  |
| UPAR | 326.4 | 0.554 | 1.29 (0.60, 2.84) | 0.6 | 0.52 | 0.90 |  |
| BMP6 | 326.4 | 0.554 | 0.81 (0.42, 1.54) | -0.6 | 0.52 | 0.90 |  |
| IL2RA | 326.4 | 0.554 | 0.84 (0.47, 1.40) | -0.6 | 0.52 | 0.90 |  |
| GLO1 | 326.4 | 0.554 | 1.12 (0.79, 1.61) | 0.6 | 0.53 | 0.90 |  |
| IL7 | 326.4 | 0.554 | 1.12 (0.79, 1.59) | 0.6 | 0.54 | 0.90 |  |
| CCL20 | 326.4 | 0.554 | 0.91 (0.68, 1.22) | -0.6 | 0.55 | 0.90 |  |
| IL1alpha | 326.3 | 0.554 | 1.41 (0.58, 5.94) | 0.6 | 0.55 | 0.90 |  |
| PLC | 326.5 | 0.554 | 1.42 (0.42, 4.90) | 0.6 | 0.57 | 0.91 |  |
| IL18BP | 326.5 | 0.554 | 0.79 (0.35, 1.78) | -0.6 | 0.57 | 0.93 |  |
| NRTN | 326.5 | 0.553 | 0.79 (0.34, 1.85) | -0.6 | 0.58 | 0.93 |  |
| CCL3 | 326.5 | 0.553 | 0.89 (0.59, 1.33) | -0.5 | 0.59 | 0.95 |  |
| PIIINP (Log) | 326.5 | 0.553 | 1.14 (0.70, 1.88) | 0.5 | 0.59 | 0.95 |  |
| LPL | 326.5 | 0.553 | 0.84 (0.45, 1.58) | -0.5 | 0.60 | 0.95 |  |
| IL20RA | 326.5 | 0.553 | 1.14 (0.70, 2.06) | 0.5 | 0.60 | 0.95 |  |
| SELE | 326.5 | 0.553 | 1.14 (0.70, 1.86) | 0.5 | 0.60 | 0.95 |  |
| MMP10 | 326.5 | 0.553 | 1.13 (0.71, 1.83) | 0.5 | 0.61 | 0.95 |  |
| TIMP4 | 326.5 | 0.553 | 0.86 (0.48, 1.55) | -0.5 | 0.61 | 0.95 |  |
| TNFRSF11A | 326.5 | 0.553 | 1.20 (0.59, 2.47) | 0.5 | 0.61 | 0.95 |  |
| THBS2 | 326.5 | 0.553 | 0.74 (0.22, 2.48) | -0.5 | 0.62 | 0.95 |  |
| IL15RA | 326.5 | 0.553 | 0.76 (0.25, 2.27) | -0.5 | 0.62 | 0.95 |  |
| CX3CL1 | 326.5 | 0.553 | 0.84 (0.41, 1.71) | -0.5 | 0.63 | 0.95 |  |
| BetaNGF | 326.5 | 0.553 | 0.80 (0.32, 2.03) | -0.5 | 0.63 | 0.95 |  |
| TRAP | 326.5 | 0.553 | 1.22 (0.55, 2.71) | 0.5 | 0.63 | 0.95 |  |
| Egfr | 326.5 | 0.553 | 1.37 (0.38, 4.88) | 0.5 | 0.63 | 0.95 |  |
| IL22R1 | 326.6 | 0.553 | 1.22 (0.56, 2.77) | 0.5 | 0.63 | 0.95 |  |
| TFPI | 326.6 | 0.553 | 0.84 (0.39, 1.72) | -0.5 | 0.63 | 0.95 |  |
| CD8A | 326.6 | 0.553 | 0.91 (0.61, 1.36) | -0.5 | 0.65 | 0.95 |  |
| APN | 326.6 | 0.553 | 1.24 (0.49, 3.13) | 0.5 | 0.65 | 0.95 |  |
| PAPPA | 326.6 | 0.553 | 1.13 (0.66, 1.97) | 0.5 | 0.65 | 0.95 |  |
| IL18 | 326.6 | 0.553 | 0.89 (0.53, 1.51) | -0.4 | 0.66 | 0.95 |  |
| DLK1 | 326.6 | 0.553 | 1.12 (0.68, 1.84) | 0.4 | 0.66 | 0.95 |  |
| DCN | 326.6 | 0.553 | 1.20 (0.53, 2.78) | 0.4 | 0.66 | 0.95 |  |
| THPO | 326.6 | 0.553 | 1.20 (0.53, 2.76) | 0.4 | 0.66 | 0.95 |  |
| CCL4 | 326.6 | 0.553 | 0.91 (0.59, 1.40) | -0.4 | 0.66 | 0.95 |  |
| VSIG2 | 326.6 | 0.553 | 1.11 (0.70, 1.77) | 0.4 | 0.67 | 0.95 |  |
| TD | 326.6 | 0.553 | 1.22 (0.48, 3.13) | 0.4 | 0.67 | 0.95 |  |
| CD40 | 326.6 | 0.553 | 1.15 (0.59, 2.27) | 0.4 | 0.68 | 0.95 |  |
| IL1RL2 | 326.6 | 0.553 | 1.17 (0.56, 1.48) | 0.4 | 0.68 | 0.95 |  |
| LOX1 | 326.6 | 0.553 | 0.91 (0.56, 1.48) | -0.4 | 0.69 | 0.95 |  |
| GP6 | 326.6 | 0.553 | 1.07 (0.76, 1.51) | 0.4 | 0.69 | 0.95 |  |
| IL1RT1 | 326.6 | 0.553 | 1.18 (0.51, 2.76) | 0.4 | 0.69 | 0.95 |  |
| NOTCH3 | 326.6 | 0.553 | 1.18 (0.52, 2.72) | 0.4 | 0.70 | 0.95 |  |
| CL24 | 326.6 | 0.553 | 1.06 (0.77, 1.47) | 0.4 | 0.70 | 0.95 |  |
| CNTN1 | 326.6 | 0.553 | 0.84 (0.35, 3.00) | -0.4 | 0.70 | 0.95 |  |
| IL2 | 326.1 | 0.554 | 4.64E+8 (0.00, 5.45E+66) | 0.4 | 0.70 | 0.95 |  |
| GH | 326.6 | 0.553 | 1.03 (0.87, 1.23) | 0.4 | 0.70 | 0.95 |  |
| CCL11 | 326.6 | 0.553 | 0.88 (0.46, 1.69) | -0.4 | 0.70 | 0.95 |  |
| ST2 | 326.6 | 0.553 | 1.12 (0.63, 1.99) | 0.4 | 0.71 | 0.95 |  |
| CD244 | 326.6 | 0.553 | 0.89 (0.48, 1.64) | -0.4 | 0.71 | 0.95 |  |
| DECR1 | 326.7 | 0.553 | 1.04 (0.86, 1.26) | 0.4 | 0.72 | 0.95 |  |
| LIFR | 326.7 | 0.553 | 1.18 (0.47, 2.96) | 0.4 | 0.72 | 0.95 |  |
| LEP | 326.7 | 0.553 | 0.94 (0.66, 1.34) | -0.4 | 0.72 | 0.95 |  |
| FABP2 | 326.7 | 0.553 | 1.07 (0.74, 1.54) | 0.4 | 0.72 | 0.95 |  |
| SCF | 326.7 | 0.553 | 1.15 (0.50, 2.69) | 0.3 | 0.74 | 0.95 |  |
| PGF | 326.7 | 0.553 | 1.17 (0.46, 2.96) | 0.3 | 0.74 | 0.95 |  |
| HGF | 326.7 | 0.553 | 1.12 (0.57, 2.20) | 0.3 | 0.75 | 0.95 |  |
| VEGFD | 326.7 | 0.553 | 1.14 (0.52, 2.49) | 0.3 | 0.75 | 0.97 |  |
| CSTB | 326.7 | 0.553 | 1.10 (0.58, 2.12) | 0.3 | 0.77 | 0.97 |  |
| CA5A | 326.7 | 0.553 | 0.95 (0.69, 1.31) | -0.3 | 0.77 | 0.97 |  |
| CXCL10 | 326.7 | 0.553 | 0.94 (0.63, 1.40) | -0.3 | 0.77 | 0.98 |  |
| HBEGF | 326.7 | 0.553 | 0.95 (0.64, 1.39) | -0.3 | 0.78 | 0.98 |  |
| IL10RB | 326.7 | 0.553 | 0.89 (0.37, 2.15) | -0.3 | 0.79 | 0.98 |  |
| CD6 | 326.7 | 0.553 | 1.07 (0.65, 1.81) | 0.3 | 0.79 | 0.98 |  |
| SLAMF1 | 326.7 | 0.553 | 0.94 (0.58, 1.50) | -0.3 | 0.79 | 0.98 |  |
| IGFBP1 | 326.7 | 0.553 | 1.04 (0.77, 1.40) | 0.3 | 0.80 | 0.98 |  |
| REN | 326.7 | 0.553 | 1.04 (0.75, 1.45) | 0.3 | 0.80 | 0.98 |  |
| CCL17 | 326.7 | 0.553 | 1.03 (0.80, 1.35) | 0.2 | 0.81 | 0.98 |  |
| CD40L | 326.7 | 0.553 | 1.03 (0.84, 1.26) | 0.2 | 0.81 | 0.98 |  |
| RETN | 326.7 | 0.553 | 1.07 (0.61, 1.90) | 0.2 | 0.81 | 0.98 |  |
| CSF1 | 326.7 | 0.553 | 0.86 (0.25, 2.95) | -0.2 | 0.82 | 0.98 |  |
| IGFBP22 | 326.7 | 0.553 | 0.94 (0.55, 1.61) | -0.2 | 0.83 | 0.98 |  |
| ITGB2 | 326.7 | 0.553 | 1.07 (0.60, 1.97) | 0.2 | 0.83 | 0.98 |  |
| CPB1 | 326.7 | 0.553 | 1.04 (0.71, 1.53) | 0.2 | 0.83 | 0.98 |  |
| IL17A | 326.7 | 0.553 | 0.96 (0.63, 1.45) | -0.2 | 0.83 | 0.98 |  |
| PTX3 | 326.7 | 0.553 | 1.07 (0.55, 2.09) | 0.2 | 0.84 | 0.98 |  |
| CD5 | 326.7 | 0.553 | 0.93 (0.47, 1.87) | -0.2 | 0.84 | 0.98 |  |
| CCL25 | 326.7 | 0.553 | 0.94 (0.52, 1.71) | -0.2 | 0.85 | 0.98 |  |
| PRSS27 | 326.7 | 0.553 | 1.06 (0.57, 1.97) | 0.2 | 0.85 | 0.98 |  |
| APR1 | 326.7 | 0.553 | 1.07 (0.53, 2.19) | 0.2 | 0.85 | 0.98 |  |
| IL17C | 326.8 | 0.553 | 0.96 (0.60, 1.50) | -0.2 | 0.85 | 0.98 |  |
| BOC | 326.8 | 0.553 | 0.91 (0.33, 2.51) | -0.2 | 0.86 | 0.98 |  |
| MARCO | 326.8 | 0.553 | 0.89 (0.25, 3.23) | -0.2 | 0.86 | 0.98 |  |
| COL1A1 | 326.8 | 0.553 | 0.94 (0.45, 1.95) | -0.2 | 0.86 | 0.98 |  |
| IL4 | 326.8 | 0.553 | 0.96 (0.60, 1.52) | -0.2 | 0.86 | 0.98 |  |
| MEPE | 326.8 | 0.553 | 1.07 (0.50, 2.25) | 0.2 | 0.86 | 0.98 |  |
| HO1 | 326.8 | 0.553 | 0.94 (0.47, 1.88) | -0.2 | 0.87 | 0.98 |  |
| ADM | 326.8 | 0.553 | 0.93 (0.40, 2.18) | -0.2 | 0.87 | 0.98 |  |
| CD4 | 326.8 | 0.553 | 0.93 (0.36, 2.48) | -0.1 | 0.89 | 0.98 |  |
| AXL | 326.8 | 0.553 | 0.94 (0.39, 2.29) | -0.1 | 0.89 | 0.98 |  |
| IL16 | 326.8 | 0.553 | 1.04 (0.60, 1.82) | 0.1 | 0.90 | 0.98 |  |
| PARP1 | 326.8 | 0.553 | 1.02 (0.75, 1.44) | 0.1 | 0.90 | 0.98 |  |
| TNFRSF10C | 326.8 | 0.553 | 1.04 (0.60, 1.82) | 0.1 | 0.90 | 0.98 |  |
| MMP9 | 326.8 | 0.553 | 0.96 (0.48, 2.00) | -0.1 | 0.91 | 0.98 |  |
| ST1A1 | 326.8 | 0.553 | 1.02 (0.74, 1.41) | 0.1 | 0.91 | 0.98 |  |
| MERTK | 326.8 | 0.553 | 1.04 (0.50, 2.19) | 0.1 | 0.91 | 0.98 |  |
| MCP3 | 326.8 | 0.553 | 1.03 (0.63, 1.63) | 0.1 | 0.91 | 0.98 |  |
| PDL2 | 326.8 | 0.553 | 0.96 (0.42, 2.18) | -0.1 | 0.91 | 0.98 |  |
| ENRAGE | 326.8 | 0.553 | 1.02 (0.72, 1.47) | 0.1 | 0.92 | 0.98 |  |
| IL10RA | 326.8 | 0.553 | 0.98 (0.59, 1.58) | -0.1 | 0.92 | 0.98 |  |
| IFNGAMMA | 326.8 | 0.553 | 0.68 (0.00, 27.20) | -0.1 | 0.93 | 0.98 |  |
| CTSL1 | 326.8 | 0.553 | 0.96 (0.43, 2.18) | -0.1 | 0.93 | 0.98 |  |
| TWEAK | 326.8 | 0.553 | 1.03 (0.48, 2.20) | 0.1 | 0.93 | 0.98 |  |
| CDCP1 | 326.8 | 0.553 | 0.98 (0.62, 1.55) | -0.1 | 0.93 | 0.98 |  |
| TNFR1 | 326.8 | 0.553 | 1.04 (0.46, 2.35) | 0.1 | 0.93 | 0.98 |  |
| CCL19 | 326.8 | 0.553 | 0.99 (0.70, 1.40) | -0.1 | 0.93 | 0.98 |  |
| IL6RA | 326.8 | 0.553 | 0.97 (0.45, 2.05) | -0.1 | 0.94 | 0.98 |  |
| SCGB32 | 326.8 | 0.553 | 0.99 (0.66, 1.48) | -0.1 | 0.94 | 0.98 |  |
| DKK1 | 326.8 | 0.553 | 0.99 (0.66, 1.48) | -0.1 | 0.95 | 0.98 |  |
| PDGFsubunitA | 326.8 | 0.553 | 0.99 (0.76, 1.29) | -0.1 | 0.95 | 0.98 |  |
| CDH5 | 326.8 | 0.553 | 0.98 (0.45, 2.16) | -0.1 | 0.96 | 0.98 |  |
| AMBP | 326.8 | 0.553 | 0.97 (0.22, 4.35) | 0.0 | 0.97 | 0.99 |  |
| TNFSF14 | 326.8 | 0.553 | 0.99 (0.63, 1.58) | 0.0 | 0.97 | 0.99 |  |
| DNER | 326.8 | 0.553 | 1.01 (0.39, 2.64) | 0.0 | 0.98 | 0.99 |  |
| VEGFA | 326.8 | 0.553 | 1.01 (0.47, 2.20) | 0.0 | 0.98 | 0.99 |  |
| IL1RT2 | 326.8 | 0.553 | 0.99 (0.43, 2.27) | 0.0 | 0.98 | 0.99 |  |
| IL6 | 326.8 | 0.553 | 1.00 (0.71, 1.42) | 0.0 | 0.98 | 0.99 |  |
| IL18R1 | 326.8 | 0.553 | 1.01 (0.52, 1.95) | 0.0 | 0.98 | 0.99 |  |
| MMP3 | 326.8 | 0.553 | 1.00 (0.62, 1.60) | 0.0 | 0.99 | 0.99 |  |

Supplemental table 6 - Proteins included in the logistic regression model, adjusted to the defined clinically relevant variables, comparing “MI” *versus* “CAD” participants. Full names of proteins can be found in *supplemental table 1*.

| Protein | AIC | r2 | OR (95% CI) | Z | *p*-value | FDR adjusted *p*-value | Proportion of significant FDR adjusted p-values (bootstraped models) |
| --- | --- | --- | --- | --- | --- | --- | --- |
| MMP9 | 521.84 | 0.086 | 2.29 (1.26, 4.47) | 2.58 | 0.010 | 0.97 | 0.75 |
| NTproBNP (Log) | 524.97 | 0.076 | 1.45 (1.03, 2.06) | 2.10 | 0.036 | 0.97 |  |
| PAI | 525.05 | 0.076 | 0.80 (0.64, 0.99) | -2.08 | 0.038 | 0.97 | 0.39 |
| MB | 525.07 | 0.076 | 1.49 (1.03, 2.21) | 2.06 | 0.040 | 0.97 |  |
| IL7 | 525.18 | 0.075 | 0.75 (0.57, 0.99) | -2.05 | 0.041 | 0.97 |  |
| CHI3L1 | 525.20 | 0.075 | 1.28 (1.01, 1.63) | 2.04 | 0.042 | 0.97 | 0.37 |
| KIM1 | 525.50 | 0.074 | 1.35 (1.00, 1.83) | 1.97 | 0.049 | 0.97 |  |
| IL1RA | 525.55 | 0.074 | 1.48 (1.00, 2.22) | 1.94 | 0.052 | 0.97 |  |
| HSTNT (Log) | 525.87 | 0.073 | 1.59 (0.98, 2.62) | 1.86 | 0.062 | 0.97 |  |
| CA5A | 525.85 | 0.073 | 1.29 (0.99, 1.70) | 1.86 | 0.063 | 0.97 |  |
| IL2RA | 526.01 | 0.072 | 1.59 (0.97, 2.64) | 1.84 | 0.066 | 0.97 |  |
| CPB1 | 526.01 | 0.072 | 0.75 (0.55, 1.02) | -1.83 | 0.067 | 0.97 |  |
| TWEAK | 526.18 | 0.072 | 0.56 (0.30, 1.05) | -1.79 | 0.074 | 0.97 |  |
| CCL16 | 526.19 | 0.072 | 1.38 (0.97, 1.97) | 1.78 | 0.075 | 0.97 |  |
| MMP7 | 526.36 | 0.071 | 0.63 (0.37, 1.05) | -1.74 | 0.083 | 0.97 |  |
| OSM | 526.39 | 0.071 | 1.25 (0.97, 1.62) | 1.73 | 0.083 | 0.97 |  |
| LIF | 525.85 | 0.073 | 0.54 (0.23, 1.02) | -1.70 | 0.089 | 0.97 |  |
| GDNF | 526.51 | 0.071 | 0.62 (0.35, 1.07) | -1.70 | 0.089 | 0.97 |  |
| CNTN1 | 526.55 | 0.071 | 0.59 (0.32, 1.08) | -1.69 | 0.091 | 0.97 |  |
| CXCL5 | 526.58 | 0.071 | 0.90 (0.80, 1.02) | -1.68 | 0.092 | 0.97 |  |
| PARP1 | 526.29 | 0.071 | 1.29 (0.97, 1.79) | 1.68 | 0.094 | 0.97 |  |
| UPAR | 526.64 | 0.070 | 1.64 (0.92, 2.97) | 1.65 | 0.098 | 0.97 |  |
| PTX3 | 526.71 | 0.070 | 1.51 (0.93, 2.47) | 1.64 | 0.10 | 0.97 |  |
| TGM2 | 526.75 | 0.070 | 1.25 (0.96, 1.63) | 1.63 | 0.10 | 0.97 |  |
| GAL4 | 526.76 | 0.070 | 0.73 (0.49, 1.06) | -1.63 | 0.10 | 0.97 |  |
| PECAM1 | 526.77 | 0.070 | 0.81 (0.62, 1.04) | -1.63 | 0.10 | 0.97 |  |
| IL18R1 | 526.80 | 0.070 | 0.66 (0.40, 1.09) | -1.62 | 0.11 | 0.97 |  |
| IGFBP2 | 526.85 | 0.070 | 1.39 (0.93, 2.08) | 1.60 | 0.11 | 0.97 |  |
| TNFRSF9 | 526.89 | 0.069 | 1.50 (0.91, 2.51) | 1.58 | 0.11 | 0.97 |  |
| IL1RT2 | 526.98 | 0.069 | 0.61 (0.33, 1.13) | -1.56 | 0.12 | 0.97 |  |
| TGFALPHA | 527.01 | 0.069 | 1.73 (0.87, 3.48) | 1.55 | 0.12 | 0.97 |  |
| TRAILR2 | 527.03 | 0.069 | 1.59 (0.89, 2.91) | 1.54 | 0.12 | 0.97 |  |
| CHIT1 | 527.02 | 0.069 | 0.87 (0.72, 1.04) | -1.53 | 0.12 | 0.97 |  |
| CASP3 | 527.07 | 0.069 | 0.89 (0.77, 1.03) | -1.53 | 0.12 | 0.97 |  |
| TRAIL | 527.16 | 0.069 | 0.57 (0.27, 1.18) | -1.50 | 0.13 | 0.97 |  |
| SLAMF7 | 527.15 | 0.069 | 0.79 (0.58, 1.07) | -1.50 | 0.13 | 0.97 |  |
| MMP1 | 527.21 | 0.068 | 1.16 (0.95, 1.42) | 1.48 | 0.14 | 0.97 |  |
| IL18 | 527.28 | 0.068 | 1.36 (0.90, 2.08) | 1.46 | 0.14 | 0.97 |  |
| PIGR | 527.33 | 0.068 | 2.59 (0.72, 9.57) | 1.44 | 0.15 | 0.97 |  |
| JAMA | 527.37 | 0.068 | 0.88 (0.73, 1.05) | -1.43 | 0.15 | 0.97 |  |
| LEP | 527.44 | 0.068 | 0.81 (0.60, 1.08) | -1.41 | 0.16 | 0.97 |  |
| GDF15 (Log) | 527.47 | 0.068 | 1.46 (0.86, 2.52) | 1.39 | 0.16 | 0.97 |  |
| MMP10 | 527.48 | 0.067 | 1.31 (0.90, 1.94) | 1.39 | 0.16 | 0.97 |  |
| CD8A | 527.49 | 0.067 | 1.25 (0.91, 1.73) | 1.39 | 0.16 | 0.97 |  |
| IL1RL2 | 527.54 | 0.067 | 0.69 (0.40, 1.17) | -1.37 | 0.17 | 0.97 |  |
| IL10RA | 527.60 | 0.067 | 0.80 (0.57, 1.10) | -1.34 | 0.18 | 0.97 |  |
| EGFR | 527.63 | 0.067 | 0.55 (0.23, 1.31) | -1.34 | 0.18 | 0.97 |  |
| ICAM2 | 527.65 | 0.067 | 0.71 (0.42, 1.17) | -1.33 | 0.18 | 0.97 |  |
| GAL3 (Log) | 527.74 | 0.067 | 1.45 (0.83, 2.56) | 1.30 | 0.20 | 0.97 |  |
| CST5 | 527.76 | 0.067 | 1.34 (0.86, 2.11) | 1.29 | 0.20 | 0.97 |  |
| ADAMTS13 | 527.78 | 0.066 | 0.54 (0.21, 1.37) | -1.28 | 0.20 | 0.97 |  |
| SERPINA12 | 527.78 | 0.066 | 0.80 (0.56, 1.12) | -1.27 | 0.20 | 0.97 |  |
| CPA1 | 527.82 | 0.066 | 0.84 (0.63, 1.10) | -1.27 | 0.20 | 0.97 |  |
| LPL | 527.82 | 0.066 | 0.76 (0.49, 1.16) | -1.27 | 0.21 | 0.97 |  |
| ST1A1 | 527.85 | 0.066 | 0.85 (0.67, 1.09) | -1.26 | 0.21 | 0.97 |  |
| CTRC | 527.87 | 0.066 | 0.84 (0.64, 1.10) | -1.24 | 0.21 | 0.97 |  |
| HSP27 | 527.89 | 0.066 | 0.75 (0.47, 1.18) | -1.23 | 0.22 | 0.97 |  |
| CTSL1 | 527.91 | 0.066 | 1.48 (0.80, 2.77) | 1.23 | 0.22 | 0.97 |  |
| GLO1 | 527.91 | 0.066 | 0.83 (0.62, 1.11) | -1.23 | 0.22 | 0.97 |  |
| IL16 | 527.91 | 0.066 | 1.32 (0.85, 2.08) | 1.23 | 0.22 | 0.97 |  |
| TNFRSF10C | 527.98 | 0.066 | 1.32 (0.84, 2.06) | 1.21 | 0.23 | 0.97 |  |
| SRC | 527.99 | 0.066 | 0.88 (0.71, 1.08) | -1.20 | 0.23 | 0.97 |  |
| PCSK9 | 528.04 | 0.066 | 0.71 (0.40, 1.25) | -1.18 | 0.24 | 0.97 |  |
| CD93 | 528.05 | 0.066 | 1.51 (0.76, 3.03) | 1.18 | 0.24 | 0.97 |  |
| IL10RB | 528.13 | 0.065 | 1.45 (0.77, 2.74) | 1.14 | 0.25 | 0.97 |  |
| LOX1 | 528.13 | 0.065 | 1.24 (0.86, 1.79) | 1.14 | 0.26 | 0.97 |  |
| PI3 | 528.14 | 0.065 | 1.26 (0.85, 1.88) | 1.14 | 0.26 | 0.97 |  |
| THPO | 528.16 | 0.065 | 0.71 (0.39, 1.28) | -1.13 | 0.26 | 0.97 |  |
| IL22RA1 | 528.06 | 0.066 | 1.53 (0.76, 3.43) | 1.13 | 0.26 | 0.97 |  |
| TFPI | 528.16 | 0.065 | 0.70 (0.38, 1.29) | -1.13 | 0.26 | 0.97 |  |
| PDGFsubunitA | 528.19 | 0.065 | 0.90 (0.75, 1.08) | -1.12 | 0.26 | 0.97 |  |
| SELP | 528.20 | 0.065 | 0.87 (0.68, 1.11) | -1.11 | 0.26 | 0.97 |  |
| TIMP4 | 528.20 | 0.065 | 0.77 (0.48, 1.22) | -1.11 | 0.27 | 0.97 |  |
| HO1 | 528.21 | 0.065 | 1.37 (0.79, 2.38) | 1.11 | 0.27 | 0.97 |  |
| GH | 528.23 | 0.065 | 0.94 (0.84, 1.05) | -1.10 | 0.27 | 0.97 |  |
| LIFR | 528.27 | 0.065 | 0.68 (0.33, 1.37) | -1.08 | 0.28 | 0.97 |  |
| IL10 | 528.25 | 0.065 | 0.83 (0.58, 1.15) | -1.07 | 0.29 | 0.97 |  |
| CCL19 | 528.30 | 0.065 | 0.87 (0.68, 1.12) | -1.07 | 0.29 | 0.97 |  |
| CXCL10 | 528.30 | 0.065 | 0.85 (0.62, 1.15) | -1.06 | 0.29 | 0.97 |  |
| THBS2 | 528.31 | 0.065 | 0.62 (0.25, 1.50) | -1.06 | 0.29 | 0.97 |  |
| CTSZ | 528.31 | 0.065 | 1.34 (0.78, 2.31) | 1.06 | 0.29 | 0.97 |  |
| GIF | 528.35 | 0.065 | 0.90 (0.74, 1.09) | -1.04 | 0.30 | 0.97 |  |
| TSLP | 528.15 | 0.065 | 0.61 (0.19, 1.40) | -1.04 | 0.30 | 0.97 |  |
| NT3 | 528.39 | 0.064 | 1.21 (0.84, 1.76) | 1.02 | 0.31 | 0.97 |  |
| ENRAGE | 528.41 | 0.064 | 1.15 (0.88, 1.50) | 1.01 | 0.31 | 0.97 |  |
| IL15RA | 528.42 | 0.064 | 0.66 (0.29, 1.49) | -1.01 | 0.31 | 0.97 |  |
| CCL23 | 528.42 | 0.064 | 0.78 (0.49, 1.26) | -1.01 | 0.31 | 0.97 |  |
| IL18BP | 528.43 | 0.064 | 1.36 (0.75, 2.48) | 1.00 | 0.32 | 0.97 |  |
| STAMBP | 528.46 | 0.064 | 0.90 (0.74, 1.10) | -0.99 | 0.32 | 0.97 |  |
| DNER | 528.49 | 0.064 | 0.69 (0.32, 1.46) | -0.97 | 0.33 | 0.97 |  |
| CCL4 | 528.50 | 0.064 | 0.87 (0.65, 1.16) | -0.97 | 0.33 | 0.97 |  |
| STK4 | 528.53 | 0.064 | 0.92 (0.77, 1.09) | -0.95 | 0.34 | 0.97 |  |
| TNFR1 | 528.55 | 0.064 | 1.35 (0.73, 2.53) | 0.94 | 0.35 | 0.97 |  |
| TNFRSF11A | 528.55 | 0.064 | 1.31 (0.75, 2.29) | 0.94 | 0.35 | 0.97 |  |
| MCP1 | 528.53 | 0.064 | 0.84 (0.57, 1.20) | -0.94 | 0.35 | 0.97 |  |
| ITGB1BP2 | 528.59 | 0.064 | 0.93 (0.80, 1.08) | -0.92 | 0.36 | 0.97 |  |
| ST2 | 528.60 | 0.064 | 0.82 (0.53, 1.26) | -0.92 | 0.36 | 0.97 |  |
| MEPE | 528.60 | 0.064 | 0.77 (0.45, 1.34) | -0.91 | 0.36 | 0.97 |  |
| IL6RA | 528.61 | 0.064 | 1.27 (0.76, 2.15) | 0.91 | 0.36 | 0.97 |  |
| ADA | 528.61 | 0.064 | 1.26 (0.77, 2.07) | 0.91 | 0.36 | 0.97 |  |
| IL33 | 528.44 | 0.064 | 2.36 (0.49, 26.53) | 0.89 | 0.37 | 0.97 |  |
| PRELP | 528.65 | 0.064 | 0.64 (0.23, 1.73) | -0.89 | 0.38 | 0.97 |  |
| TNFRSF10A | 528.67 | 0.063 | 0.76 (0.41, 1.40) | -0.88 | 0.38 | 0.97 |  |
| AGRP | 528.68 | 0.063 | 1.21 (0.79, 1.85) | 0.87 | 0.38 | 0.97 |  |
| EPCAM | 528.69 | 0.063 | 0.91 (0.71, 1.13) | -0.86 | 0.39 | 0.97 |  |
| CX3CL1 | 528.71 | 0.063 | 0.80 (0.48, 1.33) | -0.85 | 0.39 | 0.97 |  |
| IL4RA | 528.74 | 0.063 | 1.31 (0.69, 2.51) | 0.84 | 0.40 | 0.97 |  |
| HGF | 528.74 | 0.063 | 1.26 (0.73, 2.21) | 0.83 | 0.40 | 0.97 |  |
| FS | 528.75 | 0.063 | 0.80 (0.48, 1.35) | -0.83 | 0.41 | 0.97 |  |
| IL12B | 528.77 | 0.063 | 0.87 (0.62, 1.21) | -0.82 | 0.41 | 0.97 |  |
| IL24 | 528.75 | 0.063 | 1.16 (0.82, 1.72) | 0.81 | 0.42 | 0.97 |  |
| PIIINP (Log) | 528.78 | 0.063 | 0.85 (0.57, 1.26) | -0.81 | 0.42 | 0.97 |  |
| TNF | 528.70 | 0.063 | 1.41 (0.68, 3.96) | 0.80 | 0.42 | 0.97 |  |
| IL20 | 528.76 | 0.063 | 0.70 (0.26, 1.60) | -0.80 | 0.42 | 0.97 |  |
| SIRT2 | 528.81 | 0.063 | 0.94 (0.81, 1.09) | -0.79 | 0.43 | 0.97 |  |
| CCL20 | 528.83 | 0.063 | 1.09 (0.88, 1.35) | 0.78 | 0.44 | 0.97 |  |
| TPA | 528.86 | 0.063 | 0.90 (0.70, 1.17) | -0.76 | 0.45 | 0.97 |  |
| SORT1 | 528.88 | 0.063 | 0.76 (0.38, 1.54) | -0.75 | 0.45 | 0.97 |  |
| IL4 | 528.85 | 0.063 | 1.17 (0.79, 1.92) | 0.75 | 0.45 | 0.97 |  |
| IL17D | 528.88 | 0.063 | 0.77 (0.38, 1.53) | -0.75 | 0.45 | 0.97 |  |
| PAPPA | 528.89 | 0.063 | 1.16 (0.78, 1.72) | 0.74 | 0.46 | 0.97 |  |
| FLT3L | 528.89 | 0.063 | 0.83 (0.50, 1.36) | -0.74 | 0.46 | 0.97 |  |
| FAS | 528.89 | 0.063 | 1.20 (0.74, 2.00) | 0.74 | 0.46 | 0.97 |  |
| TNFR2 | 528.92 | 0.063 | 1.22 (0.71, 2.09) | 0.72 | 0.47 | 0.97 |  |
| IL20RA | 528.90 | 0.063 | 1.20 (0.75, 2.04 | 0.72 | 0.47 | 0.97 |  |
| PGLYRP1 | 528.93 | 0.063 | 1.18 (0.75, 1.85) | 0.72 | 0.47 | 0.97 |  |
| CCL17 | 528.93 | 0.063 | 0.93 (0.76, 1.14) | -0.71 | 0.48 | 0.97 |  |
| BOC | 528.94 | 0.063 | 1.22 (0.60, 2.95) | 0.71 | 0.48 | 0.97 |  |
| ACE2 | 528.95 | 0.063 | 1.14 (0.80, 1.63) | 0.70 | 0.48 | 0.97 |  |
| IL6 | 528.94 | 0.063 | 1.09 (0.86, 1.40) | 0.70 | 0.48 | 0.97 |  |
| CCL25 | 528.95 | 0.063 | 1.17 (0.76, 1.80) | 0.70 | 0.48 | 0.97 |  |
| CEACAM8 | 528.98 | 0.062 | 1.12 (0.79, 1.62) | 0.67 | 0.50 | 0.97 |  |
| TRAP | 528.99 | 0.062 | 1.22 (0.68, 2.20) | 0.67 | 0.50 | 0.97 |  |
| CD40L | 528.99 | 0.062 | 0.95 (0.82, 1.10) | -0.67 | 0.50 | 0.97 |  |
| CXCL11 | 529.00 | 0.062 | 0.93 (0.74, 1.16) | -0.67 | 0.51 | 0.97 |  |
| CD5 | 529.00 | 0.062 | 1.19 (0.71, 2.01) | 0.66 | 0.51 | 0.97 |  |
| ADM | 529.01 | 0.062 | 0.80 (0.41, 1.56) | -0.66 | 0.51 | 0.97 |  |
| GDF2 | 529.01 | 0.062 | 0.83 (0.47, 1.45) | -0.65 | 0.51 | 0.97 |  |
| AXL | 529.02 | 0.062 | 1.22 (0.67, 2.21) | 0.65 | 0.52 | 0.97 |  |
| CD84 | 529.04 | 0.062 | 0.88 (0.58, 1.32) | -0.63 | 0.53 | 0.97 |  |
| FGF23 | 529.04 | 0.062 | 0.88 (0.60, 1.30) | -0.63 | 0.53 | 0.97 |  |
| CXCL1 | 529.04 | 0.062 | 0.95 (0.81, 1.11) | -0.63 | 0.53 | 0.97 |  |
| ITGB2 | 529.05 | 0.062 | 1.16 (0.73, 1.89) | 0.63 | 0.53 | 0.97 |  |
| IL1alpha | 528.90 | 0.063 | 1.46 (0.59, 10.90) | 0.62 | 0.53 | 0.97 |  |
| CXCL9 | 529.08 | 0.062 | 0.91 (0.67, 1.24) | -0.60 | 0.55 | 0.97 |  |
| IGFBP1 | 529.09 | 0.062 | 1.08 (0.84, 1.38) | 0.59 | 0.55 | 0.97 |  |
| GP6 | 529.10 | 0.062 | 0.93 (0.71, 1.20) | -0.59 | 0.56 | 0.97 |  |
| TIE2 | 529.10 | 0.062 | 0.80 (0.37, 1.70) | -0.58 | 0.56 | 0.97 |  |
| RETN | 529.10 | 0.062 | 1.12 (0.76, 1.67) | 0.58 | 0.56 | 0.97 |  |
| DLK1 | 529.11 | 0.062 | 0.90 (0.61, 1.31) | -0.57 | 0.57 | 0.97 |  |
| PRSS27 | 529.11 | 0.062 | 1.14 (0.73, 1.77) | 0.57 | 0.57 | 0.97 |  |
| TLT2 | 529.12 | 0.062 | 1.12 (0.75, 1.69) | 0.57 | 0.57 | 0.97 |  |
| GRN | 529.12 | 0.062 | 0.84 (0.45, 1.55) | -0.57 | 0.57 | 0.97 |  |
| CCL28 | 529.13 | 0.062 | 0.84 (0.45, 1.56) | -0.56 | 0.58 | 0.97 |  |
| IFNGAMME | 529.10 | 0.062 | 0.19 (0.00, 54.55) | -0.56 | 0.58 | 0.97 |  |
| IL27 | 529.14 | 0.062 | 0.86 (0.50, 1.48) | -0.55 | 0.58 | 0.97 |  |
| NRTN | 529.15 | 0.062 | 0.87 (0.51, 1.45) | -0.54 | 0.59 | 0.97 |  |
| BP14E | 529.15 | 0.062 | 0.94 (0.73, 1.19) | -0.54 | 0.59 | 0.97 |  |
| NOTCH3 | 529.15 | 0.062 | 0.85 (0.48, 1.53) | -0.54 | 0.59 | 0.97 |  |
| DCN | 529.16 | 0.062 | 0.84 (0.44, 1.59) | -0.53 | 0.59 | 0.97 |  |
| IL13 | 529.16 | 0.062 | 1.07 (0.83, 1.42) | 0.52 | 0.60 | 0.97 |  |
| BMP6 | 529.17 | 0.062 | 0.89 (0.57, 1.37) | -0.52 | 0.60 | 0.97 |  |
| PRSS8 | 529.17 | 0.062 | 1.19 (0.61, 2.32) | 0.52 | 0.60 | 0.97 |  |
| NEMO | 529.17 | 0.062 | 0.96 (0.82, 1.13) | -0.52 | 0.60 | 0.97 |  |
| FGF5 | 529.17 | 0.062 | 0.72 (0.20, 2.56) | -0.52 | 0.60 | 0.97 |  |
| AXIN1 | 529.18 | 0.062 | 0.96 (0.83, 1.12) | -0.51 | 0.61 | 0.97 |  |
| GT | 529.19 | 0.062 | 1.08 (0.80, 1.46) | 0.50 | 0.61 | 0.97 |  |
| PLC | 529.19 | 0.062 | 1.22 (0.56, 2.67) | 0.50 | 0.62 | 0.97 |  |
| CTSD | 529.19 | 0.062 | 1.13 (0.71, 1.81) | 0.50 | 0.62 | 0.97 |  |
| MCP4 | 529.19 | 0.062 | 1.08 (0.80, 1.46) | 0.50 | 0.62 | 0.97 |  |
| OPN | 529.19 | 0.062 | 1.13 (0.70, 1.82) | 0.50 | 0.62 | 0.97 |  |
| CCL3 | 529.20 | 0.062 | 0.92 (0.68, 1.26) | -0.49 | 0.62 | 0.97 |  |
| MERTK | 529.22 | 0.062 | 0.87 (0.49, 1.54) | -0.48 | 0.63 | 0.97 |  |
| TF | 529.22 | 0.062 | 1.17 (0.60, 2.29) | 0.47 | 0.64 | 0.97 |  |
| APN | 529.23 | 0.062 | 0.85 (0.43, 1.69) | -0.46 | 0.64 | 0.97 |  |
| FABP4 | 529.23 | 0.062 | 1.09 (0.76, 1.56) | 0.46 | 0.64 | 0.97 |  |
| CDH5 | 529.23 | 0.062 | 0.87 (0.49, 1.56) | -0.46 | 0.65 | 0.97 |  |
| IL5 | 529.24 | 0.062 | 0.95 (0.78, 1.18) | -0.46 | 0.65 | 0.97 |  |
| SPON1 | 529.23 | 0.062 | 0.86 (0.45, 1.65) | -0.46 | 0.65 | 0.97 |  |
| PDL2 | 529.24 | 0.062 | 0.87 (0.47, 1.61) | -0.45 | 0.65 | 0.97 |  |
| MMP12 | 529.25 | 0.062 | 0.93 (0.68, 1.28) | -0.44 | 0.66 | 0.97 |  |
| IL2RB | 529.25 | 0.062 | 0.90 (0.56, 1.45) | -0.44 | 0.66 | 0.97 |  |
| IL8 | 529.26 | 0.061 | 0.93 (0.66, 1.31) | -0.43 | 0.67 | 0.97 |  |
| AMBP | 529.26 | 0.061 | 1.27 (0.42, 3.80) | 0.43 | 0.67 | 0.97 |  |
| VWF | 529.26 | 0.061 | 1.03 (0.88, 1.22) | 0.42 | 0.67 | 0.97 |  |
| MARCO | 529.26 | 0.061 | 1.23 (0.46, 3.31) | 0.42 | 0.67 | 0.97 |  |
| CD244 | 529.27 | 0.061 | 0.90 (0.55, 1.47) | -0.42 | 0.68 | 0.97 |  |
| SPON2 | 529.28 | 0.061 | 1.25 (0.42, 3.80) | 0.40 | 0.69 | 0.97 |  |
| hOSCAR | 529.28 | 0.061 | 1.18 (0.52, 2.66) | 0.40 | 0.69 | 0.97 |  |
| VSIG2 | 529.28 | 0.061 | 1.07 (0.77, 1.50) | 0.40 | 0.69 | 0.97 |  |
| CASP8 | 529.30 | 0.061 | 1.07 (0.76, 1.51) | 0.37 | 0.71 | 0.97 |  |
| BLMHYDROLASE | 529.31 | 0.061 | 0.92 (0.57, 1.47) | -0.37 | 0.71 | 0.97 |  |
| FGF21 | 529.31 | 0.061 | 0.97 (0.81, 1.16) | -0.36 | 0.72 | 0.97 |  |
| SCF | 529.32 | 0.061 | 0.90 (0.49, 1.63) | -0.35 | 0.73 | 0.97 |  |
| OPG | 529.32 | 0.061 | 1.11 (0.61, 2.06) | 0.35 | 0.73 | 0.97 |  |
| HAOX1 | 529.32 | 0.061 | 1.03 (0.86, 1.24) | 0.34 | 0.73 | 0.97 |  |
| BetaNGF | 529.32 | 0.061 | 1.13 (0.55, 2.39) | 0.34 | 0.73 | 0.97 |  |
| MMP2 | 529.33 | 0.061 | 1.11 (0.59, 2.10) | 0.33 | 0.74 | 0.97 |  |
| MPO | 529.33 | 0.061 | 0.89 (0.43, 1.81) | -0.33 | 0.74 | 0.97 |  |
| PSGL1 | 529.33 | 0.061 | 1.14 (0.52, 2.49) | 0.33 | 0.74 | 0.97 |  |
| CD40 | 529.33 | 0.061 | 0.92 (0.57, 1.49) | -0.33 | 0.74 | 0.97 |  |
| CCL24 | 529.34 | 0.061 | 1.04 (0.82, 1.31) | 0.33 | 0.74 | 0.97 |  |
| PDGFsubunitB | 529.34 | 0.061 | 0.98 (0.85, 1.13) | -0.31 | 0.75 | 0.97 |  |
| PON3 | 529.34 | 0.061 | 0.94 (0.63, 1.39) | -0.31 | 0.76 | 0.97 |  |
| VEGFA | 529.35 | 0.061 | 0.91 (0.51, 1.63) | -0.31 | 0.76 | 0.97 |  |
| CXCL6 | 529.35 | 0.061 | 0.96 (0.75, 1.24) | -0.30 | 0.77 | 0.97 |  |
| TNFSF13B | 529.35 | 0.061 | 1.09 (0.61, 1.96) | 0.30 | 0.77 | 0.97 |  |
| PICP (Log) | 529.36 | 0.061 | 1.08 (0.64, 1.82) | 0.28 | 0.78 | 0.97 |  |
| CITP (Log) | 529.36 | 0.061 | 0.94 (0.62, 1.43) | -0.28 | 0.78 | 0.97 |  |
| AZU1 | 529.36 | 0.061 | 1.04 (0.78, 1.40) | 0.28 | 0.78 | 0.97 |  |
| CD4 | 529.37 | 0.061 | 0.91 (0.45, 1.85) | -0.27 | 0.78 | 0.97 |  |
| FGF19 | 529.37 | 0.061 | 1.03 (0.82, 1.30) | 0.27 | 0.79 | 0.97 |  |
| XCL1 | 529.37 | 0.061 | 0.94 (0.62, 1.44) | -0.27 | 0.79 | 0.97 |  |
| TNFB | 529.37 | 0.061 | 0.94 (0.57, 1.54) | -0.26 | 0.79 | 0.97 |  |
| IL17RA | 529.37 | 0.061 | 0.95 (0.66, 1.37) | -0.26 | 0.79 | 0.97 |  |
| CD6 | 529.37 | 0.061 | 0.95 (0.63, 1.43) | -0.26 | 0.79 | 0.97 |  |
| CDCP1 | 529.38 | 0.061 | 0.95 (0.66, 1.37) | -0.25 | 0.80 | 0.97 |  |
| DKK1 | 529.38 | 0.061 | 0.96 (0.72, 1.30) | -0.25 | 0.80 | 0.97 |  |
| SOD2 | 529.38 | 0.061 | 1.23 (0.24, 6.25) | 0.25 | 0.80 | 0.97 |  |
| LAPTGFbeta1 | 529.38 | 0.061 | 0.94 (0.60, 1.49) | -0.25 | 0.81 | 0.97 |  |
| HBEGF | 529.38 | 0.061 | 1.04 (0.78, 1.39) | 0.24 | 0.81 | 0.97 |  |
| TM | 529.39 | 0.061 | 0.93 (0.49, 1.74) | -0.24 | 0.81 | 0.97 |  |
| MCP2 | 529.39 | 0.061 | 0.96 (0.69, 1.34) | -0.23 | 0.81 | 0.97 |  |
| LDLRECEPTOR | 529.39 | 0.061 | 0.95 (0.61, 1.47) | -0.23 | 0.82 | 0.97 |  |
| RARRES2 | 529.39 | 0.061 | 0.92 (0.48, 1.79) | -0.23 | 0.82 | 0.97 |  |
| VEGFD | 529.39 | 0.061 | 0.94 (0.54, 1.62) | -0.23 | 0.82 | 0.97 |  |
| SELE | 529.39 | 0.061 | 0.96 (0.68, 1.36) | -0.23 | 0.82 | 0.97 |  |
| FABP2 | 529.39 | 0.061 | 1.03 (0.79, 1.35) | 0.22 | 0.82 | 0.97 |  |
| DECR1 | 529.40 | 0.061 | 0.98 (0.85, 1.13) | -0.21 | 0.83 | 0.97 |  |
| TR | 529.40 | 0.061 | 1.04 (0.74, 1.45) | 0.21 | 0.83 | 0.97 |  |
| CCL11 | 529.40 | 0.061 | 0.95 (0.57, 1.57) | -0.21 | 0.83 | 0.97 |  |
| CXCL16 | 529.40 | 0.061 | 0.93 (0.44, 1.94) | -0.20 | 0.84 | 0.97 |  |
| CSTB | 529.40 | 0.061 | 1.05 (0.66, 1.67) | 0.20 | 0.84 | 0.97 |  |
| TNFRSF13B | 529.40 | 0.061 | 1.05 (0.65, 1.69) | 0.20 | 0.84 | 0.97 |  |
| ALCAM | 529.41 | 0.061 | 1.08 (0.49, 2.39) | 0.19 | 0.85 | 0.97 |  |
| SHPS1 | 529.41 | 0.061 | 1.05 (0.62, 1.78) | 0.18 | 0.86 | 0.97 |  |
| TFF3 | 529.41 | 0.061 | 1.05 (0.59, 1.87) | 0.17 | 0.86 | 0.97 |  |
| CD163 | 529.41 | 0.061 | 0.96 (0.61, 1.51) | -0.17 | 0.87 | 0.97 |  |
| REN | 529.42 | 0.061 | 0.98 (0.76, 1.27) | -0.16 | 0.87 | 0.97 |  |
| GAL9 | 529.42 | 0.061 | 1.06 (0.51, 2.19) | 0.15 | 0.88 | 0.98 |  |
| CSF1 | 529.42 | 0.061 | 1.07 (0.44, 2.58) | 0.15 | 0.88 | 0.98 |  |
| PSPD | 529.42 | 0.061 | 0.98 (0.76, 1.27) | -0.15 | 0.88 | 0.98 |  |
| IL17C | 529.42 | 0.061 | 1.02 (0.73, 1.45) | 0.14 | 0.89 | 0.98 |  |
| SCGB3A2 | 529.42 | 0.061 | 0.98 (0.73, 1.32) | -0.13 | 0.89 | 0.98 |  |
| COL1A1 | 529.43 | 0.061 | 1.04 (0.59, 1.81) | 0.12 | 0.90 | 0.98 |  |
| ANG1 | 529.43 | 0.061 | 1.01 (0.85, 1.19) | 0.12 | 0.90 | 0.98 |  |
| LTBR | 529.43 | 0.061 | 0.96 (0.50, 1.84) | -0.12 | 0.91 | 0.98 |  |
| IDUA | 529.43 | 0.061 | 1.03 (0.64, 1.64) | 0.12 | 0.91 | 0.98 |  |
| PDL1 | 529.43 | 0.061 | 0.97 (0.58, 1.63) | -0.12 | 0.91 | 0.98 |  |
| TNFRSF14 | 529.43 | 0.061 | 1.03 (0.64, 1.64) | 0.11 | 0.91 | 0.98 |  |
| RAGE | 529.43 | 0.061 | 0.97 (0.57, 1.65) | -0.11 | 0.91 | 0.98 |  |
| EPHB4 | 529.43 | 0.061 | 1.04 (0.54, 2.01) | 0.11 | 0.91 | 0.98 |  |
| PGF | 529.43 | 0.061 | 0.97 (0.48, 1.94) | -0.10 | 0.92 | 0.98 |  |
| IL1RT1 | 529.43 | 0.061 | 0.97 (0.53, 1.77) | -0.09 | 0.93 | 0.98 |  |
| TRANCE | 529.44 | 0.061 | 1.01 (0.72, 1.43) | 0.08 | 0.94 | 0.98 |  |
| PAR1 | 529.44 | 0.061 | 1.02 (0.63, 1.63) | 0.06 | 0.95 | 0.98 |  |
| IGFBP7 | 529.44 | 0.061 | 0.98 (0.54, 1.79) | -0.06 | 0.95 | 0.98 |  |
| IL17A | 529.44 | 0.061 | 0.99 (0.73, 1.34) | -0.05 | 0.96 | 0.99 |  |
| CCL15 | 529.44 | 0.061 | 0.99 (0.60, 1.63) | -0.04 | 0.96 | 0.99 |  |
| MMP3 | 529.44 | 0.061 | 1.01 (0.68, 1.49) | 0.04 | 0.97 | 0.99 |  |
| MCP3 | 529.44 | 0.061 | 0.99 (0.68, 1.47) | -0.04 | 0.97 | 0.99 |  |
| TNFSF14 | 529.44 | 0.061 | 1.01 (0.71, 1.44) | 0.04 | 0.97 | 0.99 |  |
| UPA | 529.44 | 0.061 | 0.99 (0.60, 1.62) | -0.04 | 0.97 | 0.99 |  |
| SLAMF1 | 529.44 | 0.061 | 0.99 (0.68, 1.47) | -0.03 | 0.98 | 0.99 |  |
| ARTN | 529.44 | 0.061 | 1.01 (0.59, 1.80) | 0.03 | 0.98 | 0.99 |  |
| PRTN3 | 529.44 | 0.061 | 0.99 (0.50, 1.97) | -0.03 | 0.98 | 0.99 |  |
| IL2 | 528.32 | 0.065 | 3.73e27 (0, NA) | 0.02 | 0.98 | 0.99 |  |
| IgGFcreceptorIlb | 529.44 | 0.061 | 1.00 (0.77, 1.30) | 0.01 | 0.99 | 0.99 |  |
| KLK6 | 529.44 | 0.061 | 1.00 (0.59, 1.70) | 0.01 | 1.00 | 0.99 |  |

Supplemental table 7- Proteins included in the logistic regression model, adjusted to the defined clinically relevant variables and medication, comparing “CAD” *versus* “no CAD/no MI” participants. Full names of proteins can be found in *supplemental table 1*.

| Protein | AIC | *r*^2^ | OR (95% CI) | Z | *p*-value | FDR adjusted *p*-value |
| --- | --- | --- | --- | --- | --- | --- |
| GAL4 | 238.1 | 0.704 | 3.27 (1.54, 1.84) | 3.01 | 0.003 | 0.95 |
| MMP7 | 237.5 | 0.706 | 3.14 (1.53, 1.69) | 2.95 | 0.003 | 0.85 |
| PAI | 239 | 0.703 | 1.79 (1.22, 2.04) | 2.87 | 0.004 | 0.92 |
| CCL23 | 240.2 | 0.700 | 0.32 (0.13, 1.92) | -2.7 | 0.01 | 0.98 |
| ADAMTS13 | 240.4 | 0.700 | 8.66 (1.8, 1.78) | 2.6 | 0.01 | 0.61 |
| TPA | 241.0 | 0.699 | 2.02 (1.19, 1.79) | 2.57 | 0.01 | 0.95 |
| OPN | 241.8 | 0.697 | 0.41 (0.19, 1.88) | -2.43 | 0.02 | 0.95 |
| TGFALPHA | 242.5 | 0.695 | 0.28 (0.09, 1.84) | -2.28 | 0.02 | 0.95 |
| TRANCE | 242.7 | 0.695 | 0.52 (0.29, 2) | -2.22 | 0.03 | 0.98 |
| IL8 | 243.2 | 0.694 | 1.81 (1.06, 1.99) | 2.19 | 0.03 | 0.95 |
| OSM | 243.3 | 0.694 | 0.61 (0.38, 1.77) | -2.09 | 0.04 | 0.95 |
| MB | 243.5 | 0.693 | 0.5 (0.25, 1.91) | -2.06 | 0.04 | 0.95 |
| TNFRSF9 | 243.5 | 0.693 | 0.47 (0.22, 1.72) | -2.04 | 0.04 | 0.99 |
| CA5A | 243.6 | 0.693 | 0.66 (0.44, 1.99) | -2.02 | 0.04 | 0.88 |
| FGF5 | 243.5 | 0.693 | 13.09 (1.15, 1.98) | 2 | 0.05 | 0.96 |
| NT3 | 244 | 0.693 | 1.94 (1, 2.02) | 1.98 | 0.05 | 0.99 |
| IL12B | 244.1 | 0.692 | 0.61 (0.37, 1.8) | -1.96 | 0.05 | 0.98 |
| CD8A | 243.9 | 0.693 | 0.59 (0.34, 1.82) | -1.94 | 0.05 | 0.95 |
| PSGL1 | 243.9 | 0.693 | 3.65 (1.02, 1.72) | 1.93 | 0.05 | 0.85 |
| TM | 244.2 | 0.692 | 0.31 (0.09, 1.89) | -1.89 | 0.06 | 0.81 |
| ITGB2 | 244.3 | 0.692 | 0.42 (0.17, 1.96) | -1.85 | 0.06 | 0.89 |
| TLT2 | 244.5 | 0.691 | 0.5 (0.24, 1.85) | -1.83 | 0.07 | 0.92 |
| IL24 | 243.7 | 0.693 | 2.91 (1.03, 1.83) | 1.82 | 0.07 | 0.98 |
| IL2RA | 243.9 | 0.693 | 0.56 (0.27, 1.82) | -1.81 | 0.07 | 0.89 |
| MMP10 | 244.5 | 0.691 | 0.55 (0.28, 1.82) | -1.8 | 0.07 | 0.98 |
| IL16 | 244.5 | 0.692 | 0.51 (0.24, 1.78) | -1.79 | 0.07 | 0.83 |
| LTBR | 244.8 | 0.691 | 0.38 (0.12, 1.9) | -1.73 | 0.08 | 0.92 |
| CTRC | 245 | 0.69 | 0.73 (0.51, 1.9) | -1.72 | 0.09 | 0.77 |
| GH | 244.9 | 0.691 | 1.2 (0.97, 1.91) | 1.69 | 0.09 | 0.77 |
| TRAP | 245 | 0.691 | 0.41 (0.14, 1.93) | -1.68 | 0.09 | 0.94 |
| CD93 | 245 | 0.69 | 0.33 (0.09, 1.85) | -1.68 | 0.09 | 0.95 |
| NTproBNP (Log) | 245.1 | 0.69 | 0.59 (0.31, 2.08) | -1.65 | 0.1 | 0.99 |
| MPO | 244.5 | 0.691 | 4.69 (0.9, 1.88) | 1.64 | 0.1 | 0.93 |
| PGLYRP1 | 245.2 | 0.690 | 0.56 (0.28, 1.74) | -1.61 | 0.11 | 0.95 |
| IL10 | 245 | 0.690 | 1.57 (0.94, 1.93) | 1.61 | 0.11 | 0.98 |
| IL18BP | 245.3 | 0.690 | 0.44 (0.15, 1.92) | -1.59 | 0.11 | 0.95 |
| KIM1 | 245.3 | 0.690 | 0.66 (0.39, 1.9) | -1.58 | 0.11 | 0.8 |
| PRTN3 | 244.6 | 0.691 | 5.47 (0.88, 1.89) | 1.58 | 0.11 | 0.95 |
| LDLRECEPTOR | 245.4 | 0.690 | 0.56 (0.26, 1.97) | -1.55 | 0.12 | 0.89 |
| CXCL6 | 245.5 | 0.689 | 1.35 (0.92, 2) | 1.55 | 0.12 | 0.98 |
| IGFBP2 | 245.4 | 0.69 | 0.59 (0.29, 1.68) | -1.54 | 0.12 | 0.95 |
| PECAM1 | 245.5 | 0.689 | 1.43 (0.91, 1.95) | 1.54 | 0.12 | 0.95 |
| IL7 | 245.5 | 0.689 | 1.4 (0.91, 1.91) | 1.54 | 0.12 | 0.95 |
| EPCAM | 245.5 | 0.689 | 1.36 (0.92, 1.93) | 1.53 | 0.12 | 0.94 |
| TGM2 | 245.6 | 0.689 | 0.73 (0.48, 1.83) | -1.53 | 0.13 | 0.85 |
| FABP2 | 245.5 | 0.689 | 1.46 (0.9, 1.88) | 1.51 | 0.13 | 0.85 |
| CST5 | 245.6 | 0.689 | 0.58 (0.27, 1.73) | -1.49 | 0.14 | 0.95 |
| TIE2 | 245.6 | 0.689 | 0.39 (0.11, 1.86) | -1.48 | 0.14 | 0.77 |
| MARCO | 245.7 | 0.689 | 0.27 (0.05, 1.94) | -1.47 | 0.14 | 0.85 |
| TNFR2 | 245.8 | 0.689 | 0.56 (0.24, 1.81) | -1.43 | 0.15 | 0.89 |
| AZU1 | 245.5 | 0.689 | 1.67 (0.88, 1.89) | 1.42 | 0.16 | 0.93 |
| PGF | 245.8 | 0.689 | 0.44 (0.14, 1.91) | -1.42 | 0.16 | 0.48 |
| CASP3 | 245.9 | 0.689 | 1.19 (0.94, 1.94) | 1.41 | 0.16 | 0.95 |
| UPAR | 245.8 | 0.689 | 0.5 (0.19, 1.84) | -1.41 | 0.16 | 0.95 |
| CSF1 | 245.9 | 0.689 | 0.32 (0.06, 1.89) | -1.39 | 0.16 | 0.99 |
| CITP (Log) | 245.9 | 0.689 | 0.63 (0.32, 1.78) | -1.37 | 0.17 | 1 |
| PARP1 | 245.7 | 0.689 | 0.69 (0.39, 1.78) | -1.37 | 0.17 | 0.89 |
| JAMA | 246.1 | 0.688 | 1.24 (0.9, 1.93) | 1.31 | 0.19 | 0.95 |
| SELP | 246.2 | 0.688 | 1.32 (0.87, 1.93) | 1.29 | 0.2 | 0.91 |
| CDCP1 | 246.2 | 0.688 | 0.68 (0.38, 1.92) | -1.28 | 0.2 | 0.95 |
| FAS | 246.3 | 0.688 | 1.62 (0.74, 1.81) | 1.28 | 0.2 | 0.95 |
| IL17RA | 246.2 | 0.688 | 0.66 (0.35, 1.92) | -1.27 | 0.2 | 0.89 |
| GIF | 246.2 | 0.688 | 1.23 (0.9, 1.73) | 1.26 | 0.21 | 0.77 |
| BLMHYDROLASE | 246.3 | 0.688 | 1.72 (0.74, 1.89) | 1.25 | 0.21 | 0.92 |
| PCSK9 | 246.3 | 0.688 | 2.01 (0.68, 1.8) | 1.25 | 0.21 | 0.95 |
| VWF | 246.3 | 0.688 | 1.21 (0.9, 1.93) | 1.24 | 0.21 | 0.95 |
| AXL | 246.3 | 0.688 | 0.52 (0.18, 1.88) | -1.24 | 0.21 | 0.95 |
| CCL16 | 246.4 | 0.688 | 0.7 (0.39, 1.98) | -1.22 | 0.22 | 0.95 |
| CEACAM8 | 246.4 | 0.688 | 0.69 (0.37, 1.81) | -1.21 | 0.23 | 0.84 |
| GAL9 | 246.4 | 0.688 | 0.45 (0.12, 1.9) | -1.21 | 0.23 | 0.77 |
| PICP (Log) | 246.4 | 0.688 | 0.59 (0.25, 1.76) | -1.21 | 0.23 | 1 |
| MCP1 | 246.1 | 0.688 | 1.82 (0.8, 1.83) | 1.21 | 0.23 | 0.92 |
| VEGFD | 246.4 | 0.688 | 0.58 (0.23, 1.87) | -1.2 | 0.23 | 0.89 |
| PI3 | 246.4 | 0.687 | 0.7 (0.38, 1.87) | -1.2 | 0.23 | 0.94 |
| BOC | 246.4 | 0.687 | 0.45 (0.12, 1.85) | -1.19 | 0.23 | 0.73 |
| GRN | 246.4 | 0.688 | 2.02 (0.65, 1.77) | 1.18 | 0.24 | 0.92 |
| IL18 | 246.5 | 0.687 | 0.67 (0.34, 1.85) | -1.16 | 0.24 | 0.77 |
| AGRP | 246.6 | 0.687 | 0.66 (0.33, 1.92) | -1.16 | 0.25 | 0.85 |
| PDL1 | 246.5 | 0.687 | 0.61 (0.27, 1.81) | -1.15 | 0.25 | 0.98 |
| IL6RA | 246.5 | 0.687 | 0.55 (0.19, 1.8) | -1.15 | 0.25 | 0.93 |
| CXCL9 | 246.5 | 0.687 | 0.76 (0.47, 1.84) | -1.15 | 0.25 | 0.95 |
| PRELP | 246.6 | 0.687 | 2.58 (0.51, 1.86) | 1.14 | 0.26 | 0.82 |
| CD163 | 246.6 | 0.687 | 0.64 (0.29, 1.92) | -1.1 | 0.27 | 0.92 |
| TRAILR2 | 246.6 | 0.687 | 0.57 (0.21, 1.83) | -1.1 | 0.27 | 0.77 |
| MEPE | 246.6 | 0.687 | 0.59 (0.23, 1.85) | -1.1 | 0.27 | 0.95 |
| CXCL1 | 246.7 | 0.687 | 1.16 (0.89, 1.96) | 1.09 | 0.28 | 0.77 |
| EPHB4 | 246.7 | 0.687 | 0.56 (0.19, 1.92) | -1.08 | 0.28 | 0.89 |
| ITGB1BP2 | 246.8 | 0.687 | 1.14 (0.89, 1.89) | 1.04 | 0.3 | 0.85 |
| CTSD | 246.8 | 0.687 | 1.52 (0.69, 1.81) | 1.03 | 0.3 | 0.95 |
| HSTNT (Log) | 246.8 | 0.687 | 0.66 (0.3, 1.97) | -1.02 | 0.31 | 1 |
| GDF2 | 246.8 | 0.687 | 1.58 (0.67, 1.87) | 1.02 | 0.31 | 0.85 |
| GDNF | 246.8 | 0.687 | 1.58 (0.66, 1.81) | 1.02 | 0.31 | 0.95 |
| TNFR1 | 246.8 | 0.687 | 0.61 (0.23, 1.86) | -1 | 0.32 | 0.95 |
| CX3CL1 | 246.8 | 0.687 | 0.66 (0.28, 1.82) | -1 | 0.32 | 0.99 |
| ADA | 246.9 | 0.687 | 1.5 (0.68, 1.89) | 1 | 0.32 | 0.99 |
| IL10RB | 246.9 | 0.687 | 0.59 (0.2, 1.83) | -1 | 0.32 | 0.98 |
| TNFRSF11A | 246.9 | 0.687 | 0.66 (0.29, 1.89) | -0.99 | 0.32 | 0.77 |
| PDGFsubunitA | 246.9 | 0.687 | 1.18 (0.85, 1.92) | 0.99 | 0.32 | 0.95 |
| LOX1 | 246.9 | 0.686 | 0.75 (0.41, 1.82) | -0.96 | 0.34 | 0.77 |
| SRC | 246.9 | 0.686 | 1.19 (0.84, 1.88) | 0.96 | 0.34 | 0.73 |
| MMP9 | 247 | 0.686 | 0.6 (0.2, 1.82) | -0.95 | 0.34 | 0.89 |
| CDH5 | 247 | 0.686 | 0.62 (0.22, 1.88) | -0.93 | 0.35 | 0.92 |
| UPA | 247.1 | 0.686 | 1.35 (0.69, 1.81) | 0.92 | 0.36 | 0.95 |
| CCL20 | 247 | 0.686 | 0.84 (0.58, 1.81) | -0.92 | 0.36 | 0.99 |
| CSTB | 247 | 0.686 | 0.71 (0.33, 1.84) | -0.91 | 0.36 | 0.92 |
| HSP27 | 247 | 0.686 | 1.43 (0.66, 1.88) | 0.9 | 0.37 | 0.88 |
| CD6 | 247.1 | 0.686 | 1.35 (0.69, 1.86) | 0.9 | 0.37 | 0.95 |
| STK4 | 247.1 | 0.686 | 1.14 (0.85, 1.89) | 0.88 | 0.38 | 0.77 |
| RAGE | 247.1 | 0.686 | 0.67 (0.28, 1.87) | -0.88 | 0.38 | 0.77 |
| NEMO | 247.1 | 0.686 | 1.13 (0.86, 1.87) | 0.88 | 0.38 | 0.88 |
| AMBP | 247.1 | 0.686 | 0.41 (0.06, 1.89) | -0.88 | 0.38 | 0.82 |
| CD244 | 247.1 | 0.686 | 0.68 (0.27, 1.89) | -0.86 | 0.39 | 0.95 |
| HGF | 247.1 | 0.686 | 0.71 (0.3, 1.83) | -0.86 | 0.39 | 0.98 |
| PAR1 | 247.1 | 0.686 | 0.73 (0.34, 1.85) | -0.85 | 0.39 | 0.77 |
| SLAMF7 | 247.1 | 0.686 | 0.79 (0.47, 1.88) | -0.85 | 0.4 | 0.48 |
| FS | 247.1 | 0.686 | 1.44 (0.62, 1.86) | 0.85 | 0.4 | 0.77 |
| CXCL5 | 247.2 | 0.686 | 1.09 (0.89, 1.88) | 0.84 | 0.4 | 0.98 |
| ARTN | 247.2 | 0.686 | 0.68 (0.26, 1.84) | -0.83 | 0.41 | 0.98 |
| IL27 | 247.2 | 0.686 | 1.46 (0.6, 1.81) | 0.83 | 0.41 | 0.77 |
| GAL3 (Log) | 247.2 | 0.686 | 0.64 (0.22, 1.87) | -0.81 | 0.42 | 1.00 |
| XCL1 | 247.2 | 0.686 | 0.78 (0.41, 1.87) | -0.8 | 0.42 | 0.82 |
| VSIG2 | 247.2 | 0.686 | 0.79 (0.45, 1.9) | -0.8 | 0.43 | 0.82 |
| TFPI | 247.2 | 0.686 | 0.72 (0.32, 1.87) | -0.8 | 0.43 | 0.92 |
| FLT3L | 247.2 | 0.686 | 1.43 (0.59, 1.79) | 0.79 | 0.43 | 0.98 |
| AXIN1 | 247.2 | 0.686 | 1.11 (0.86, 1.88) | 0.79 | 0.43 | 0.95 |
| PSPD | 247.2 | 0.686 | 1.2 (0.76, 1.79) | 0.78 | 0.43 | 0.94 |
| REN | 247.3 | 0.686 | 1.19 (0.77, 1.79) | 0.77 | 0.44 | 0.77 |
| OPG | 247.3 | 0.686 | 1.57 (0.5, 1.82) | 0.76 | 0.45 | 0.89 |
| IL18R1 | 247.3 | 0.686 | 1.41 (0.58, 1.78) | 0.75 | 0.45 | 0.98 |
| CD5 | 247.3 | 0.686 | 0.71 (0.29, 1.79) | -0.75 | 0.45 | 0.98 |
| MMP1 | 247.3 | 0.686 | 0.88 (0.64, 1.75) | -0.75 | 0.45 | 0.96 |
| BNP | 247.3 | 0.686 | 0.88 (0.62, 1.9) | -0.74 | 0.46 | 0.85 |
| CCL24 | 247.3 | 0.686 | 1.17 (0.76, 1.8) | 0.73 | 0.47 | 0.92 |
| SERPINA12 | 247.3 | 0.686 | 0.83 (0.5, 1.9) | -0.73 | 0.47 | 0.77 |
| HOSCAR | 247.3 | 0.686 | 0.6 (0.15, 1.82) | -0.72 | 0.47 | 0.85 |
| COL1A1 | 247.4 | 0.686 | 0.71 (0.27, 1.79) | -0.71 | 0.48 | 0.95 |
| CCL19 | 247.4 | 0.686 | 0.87 (0.58, 1.83) | -0.71 | 0.48 | 0.97 |
| CPA1 | 247.4 | 0.686 | 1.17 (0.76, 1.83) | 0.7 | 0.48 | 0.95 |
| IL17D | 247.4 | 0.685 | 1.27 (0.59, 1.89) | 0.68 | 0.5 | 0.77 |
| ST2 | 247.4 | 0.685 | 1.27 (0.64, 1.82) | 0.67 | 0.5 | 0.95 |
| CTSZ | 247.4 | 0.685 | 0.72 (0.26, 1.87) | -0.66 | 0.51 | 0.95 |
| IL33 | 247.5 | 0.685 | 13.76 (0.01, 1.86) | 0.65 | 0.52 | 0.98 |
| TNFRSF10C | 247.4 | 0.685 | 0.77 (0.35, 1.86) | -0.65 | 0.52 | 0.92 |
| ADM | 247.5 | 0.685 | 0.72 (0.26, 1.89) | -0.64 | 0.52 | 0.38 |
| IL15RA | 247.5 | 0.685 | 0.68 (0.2, 1.87) | -0.63 | 0.53 | 0.98 |
| SELE | 247.5 | 0.685 | 1.22 (0.66, 1.83) | 0.63 | 0.53 | 0.93 |
| PLC | 247.5 | 0.685 | 0.63 (0.15, 1.84) | -0.62 | 0.54 | 0.92 |
| CD40 | 247.5 | 0.685 | 0.78 (0.35, 1.81) | -0.62 | 0.54 | 0.98 |
| BMP6 | 247.5 | 0.685 | 0.79 (0.35, 1.8) | -0.62 | 0.54 | 0.38 |
| CCL3 | 247.5 | 0.685 | 0.86 (0.53, 1.81) | -0.61 | 0.54 | 0.85 |
| THPO | 247.5 | 0.685 | 1.39 (0.48, 1.88) | 0.6 | 0.55 | 0.85 |
| TR | 247.5 | 0.685 | 0.84 (0.48, 1.86) | -0.6 | 0.55 | 0.92 |
| CCL25 | 247.5 | 0.685 | 1.24 (0.62, 1.9) | 0.6 | 0.55 | 0.99 |
| PIGR | 247.5 | 0.685 | 1.89 (0.24, 1.84) | 0.6 | 0.55 | 0.77 |
| TNF | 247.3 | 0.686 | 0.53 (0.05, 1.79) | -0.6 | 0.55 | 0.98 |
| IDUA | 247.5 | 0.685 | 0.77 (0.32, 1.87) | -0.59 | 0.55 | 0.77 |
| PON3 | 247.5 | 0.685 | 0.81 (0.4, 1.81) | -0.59 | 0.55 | 0.95 |
| EGFR | 247.5 | 0.685 | 1.56 (0.34, 1.83) | 0.58 | 0.56 | 0.95 |
| TNFRSF13B | 247.5 | 0.685 | 0.81 (0.39, 1.85) | -0.58 | 0.56 | 0.85 |
| IL17A | 247.5 | 0.685 | 1.14 (0.74, 1.82) | 0.57 | 0.57 | 0.95 |
| IL1RA | 247.5 | 0.685 | 0.83 (0.43, 1.87) | -0.57 | 0.57 | 0.73 |
| STAMBP | 247.6 | 0.685 | 1.1 (0.79, 1.86) | 0.55 | 0.58 | 0.99 |
| ST1A1 | 247.6 | 0.685 | 1.12 (0.75, 1.88) | 0.55 | 0.58 | 0.99 |
| BetaNGF | 247.6 | 0.685 | 0.78 (0.3, 1.81) | -0.54 | 0.59 | 0.98 |
| HBEGF | 247.6 | 0.685 | 0.87 (0.51, 1.77) | -0.54 | 0.59 | 0.85 |
| MMP3 | 247.6 | 0.685 | 0.86 (0.49, 1.87) | -0.53 | 0.59 | 0.95 |
| IL2RB | 247.6 | 0.685 | 1.26 (0.54, 1.77) | 0.53 | 0.6 | 0.95 |
| CCL28 | 247.6 | 0.685 | 0.73 (0.23, 1.79) | -0.53 | 0.6 | 0.98 |
| DNER | 247.6 | 0.685 | 0.73 (0.21, 1.84) | -0.52 | 0.6 | 0.98 |
| CCL15 | 247.6 | 0.685 | 1.25 (0.54, 1.84) | 0.51 | 0.61 | 0.95 |
| IL1RT2 | 247.6 | 0.685 | 1.33 (0.44, 1.79) | 0.51 | 0.61 | 0.95 |
| IL17C | 247.6 | 0.685 | 0.87 (0.51, 1.88) | -0.51 | 0.61 | 0.95 |
| PDL2 | 247.6 | 0.685 | 0.75 (0.24, 1.88) | -0.5 | 0.61 | 0.85 |
| TNFRSF10A | 247.6 | 0.685 | 1.3 (0.46, 1.84) | 0.5 | 0.62 | 0.77 |
| IGFBP1 | 247.6 | 0.685 | 0.91 (0.63, 1.82) | -0.49 | 0.62 | 0.94 |
| FGF19 | 247.6 | 0.685 | 1.09 (0.75, 1.86) | 0.48 | 0.63 | 0.98 |
| TNFRSF14 | 247.6 | 0.685 | 0.81 (0.35, 1.84) | -0.47 | 0.63 | 0.89 |
| TRAIL | 247.6 | 0.685 | 0.72 (0.17, 1.86) | -0.47 | 0.64 | 0.95 |
| TSLP | 247.6 | 0.685 | 0.81 (0.32, 1.81) | -0.47 | 0.64 | 0.95 |
| BP14E | 247.6 | 0.685 | 1.11 (0.72, 1.84) | 0.46 | 0.64 | 0.98 |
| CNTN1 | 247.6 | 0.685 | 1.3 (0.43, 1.84) | 0.46 | 0.64 | 0.92 |
| SIRT2 | 247.7 | 0.685 | 1.06 (0.83, 1.86) | 0.45 | 0.65 | 0.98 |
| MCP3 | 247.7 | 0.685 | 0.87 (0.46, 1.83) | -0.44 | 0.66 | 0.95 |
| GLO1 | 247.7 | 0.685 | 1.11 (0.7, 1.85) | 0.44 | 0.66 | 0.77 |
| IL20RA | 247.7 | 0.685 | 0.87 (0.4, 1.83) | -0.44 | 0.66 | 0.95 |
| IL1RT1 | 247.7 | 0.685 | 0.77 (0.24, 1.84) | -0.43 | 0.66 | 0.95 |
| MMP2 | 247.7 | 0.685 | 1.26 (0.45, 1.84) | 0.43 | 0.67 | 0.95 |
| DLK1 | 247.7 | 0.685 | 0.86 (0.44, 1.84) | -0.43 | 0.67 | 0.93 |
| APN | 247.7 | 0.685 | 0.79 (0.27, 1.85) | -0.43 | 0.67 | 0.94 |
| PIIINP (Log) | 247.7 | 0.685 | 0.88 (0.47, 1.84) | -0.41 | 0.68 | 1 |
| TFF3 | 247.7 | 0.685 | 0.83 (0.33, 1.85) | -0.4 | 0.69 | 0.91 |
| IL6 | 247.7 | 0.685 | 1.08 (0.74, 1.85) | 0.39 | 0.69 | 0.77 |
| FGF23 | 247.7 | 0.685 | 1.11 (0.64, 1.82) | 0.39 | 0.7 | 0.77 |
| CD4 | 247.7 | 0.685 | 0.77 (0.21, 1.86) | -0.39 | 0.7 | 0.88 |
| IL1alpha | 247.7 | 0.685 | 1.45 (0.09, 1.85) | 0.38 | 0.7 | 0.95 |
| DCN | 247.7 | 0.685 | 1.22 (0.43, 1.84) | 0.38 | 0.71 | 0.85 |
| SCGB3A2 | 247.7 | 0.685 | 0.91 (0.53, 1.78) | -0.38 | 0.71 | 0.95 |
| PRSS27 | 247.7 | 0.685 | 1.16 (0.54, 1.83) | 0.37 | 0.71 | 0.77 |
| PRSS8 | 247.7 | 0.685 | 0.82 (0.27, 1.86) | -0.37 | 0.71 | 0.85 |
| FABP4 | 247.7 | 0.685 | 0.9 (0.51, 1.85) | -0.36 | 0.72 | 0.92 |
| HAOX1 | 247.7 | 0.685 | 1.06 (0.78, 1.81) | 0.36 | 0.72 | 0.89 |
| TNFSF14 | 247.7 | 0.685 | 0.9 (0.48, 1.81) | -0.35 | 0.72 | 0.95 |
| CHI3L1 | 247.7 | 0.685 | 0.93 (0.63, 1.84) | -0.35 | 0.73 | 0.95 |
| IL22RA1 | 247.7 | 0.685 | 0.76 (0.14, 1.8) | -0.34 | 0.73 | 0.98 |
| IgGFcreceptorIlb | 247.7 | 0.685 | 1.08 (0.69, 1.8) | 0.34 | 0.73 | 0.85 |
| LIF | 247.7 | 0.685 | 0.85 (0.36, 1.84) | -0.34 | 0.73 | 0.99 |
| IFNGAMMA | 247.8 | 0.685 | 2.71 (0, 1.84) | 0.34 | 0.74 | 0.98 |
| HO1 | 247.8 | 0.685 | 0.86 (0.36, 1.82) | -0.33 | 0.74 | 0.82 |
| PAPPA | 247.8 | 0.685 | 0.9 (0.47, 1.85) | -0.33 | 0.74 | 0.77 |
| KLK6 | 247.8 | 0.685 | 0.87 (0.37, 1.84) | -0.33 | 0.74 | 0.95 |
| LPL | 247.8 | 0.685 | 0.89 (0.43, 1.81) | -0.32 | 0.75 | 0.85 |
| CCL17 | 247.8 | 0.685 | 1.06 (0.75, 1.85) | 0.32 | 0.75 | 0.85 |
| VEGFA | 247.8 | 0.685 | 0.86 (0.31, 1.83) | -0.31 | 0.76 | 0.95 |
| TIMP4 | 247.8 | 0.685 | 0.89 (0.42, 1.84) | -0.3 | 0.76 | 0.92 |
| GT | 247.8 | 0.685 | 0.93 (0.57, 1.85) | -0.29 | 0.77 | 0.85 |
| ICAM2 | 247.8 | 0.685 | 0.88 (0.36, 1.85) | -0.28 | 0.78 | 0.95 |
| CTSL1 | 247.8 | 0.685 | 0.87 (0.33, 1.84) | -0.28 | 0.78 | 0.85 |
| TWEAK | 247.8 | 0.685 | 1.14 (0.42, 1.84) | 0.27 | 0.79 | 0.99 |
| SOD2 | 247.8 | 0.685 | 1.43 (0.1, 1.84) | 0.27 | 0.79 | 0.77 |
| SPON1 | 247.8 | 0.685 | 0.87 (0.31, 1.84) | -0.26 | 0.79 | 0.93 |
| GDF15 (Log) | 247.8 | 0.685 | 1.09 (0.56, 1.82) | 0.25 | 0.8 | 0.92 |
| CD40L | 247.8 | 0.685 | 1.03 (0.8, 1.85) | 0.25 | 0.8 | 0.48 |
| LEP | 247.8 | 0.685 | 0.94 (0.59, 1.85) | -0.24 | 0.81 | 0.85 |
| IGFBP7 | 247.8 | 0.685 | 1.14 (0.39, 1.81) | 0.24 | 0.81 | 0.95 |
| ACE2 | 247.8 | 0.685 | 0.93 (0.52, 1.85) | -0.23 | 0.82 | 0.85 |
| CD84 | 247.8 | 0.685 | 0.92 (0.44, 1.81) | -0.23 | 0.82 | 0.77 |
| ENRAGE | 247.8 | 0.685 | 0.95 (0.61, 1.81) | -0.23 | 0.82 | 0.98 |
| ALCAM | 247.8 | 0.685 | 1.18 (0.27, 1.83) | 0.22 | 0.82 | 0.89 |
| LAPTGFbeta1 | 247.8 | 0.685 | 1.09 (0.49, 1.85) | 0.21 | 0.83 | 0.95 |
| CXCL11 | 247.8 | 0.685 | 1.04 (0.7, 1.84) | 0.21 | 0.83 | 0.95 |
| NRTN | 247.8 | 0.685 | 1.09 (0.47, 1.83) | 0.19 | 0.85 | 0.99 |
| SHPS1 | 247.8 | 0.685 | 1.09 (0.43, 1.84) | 0.19 | 0.85 | 0.95 |
| CXCL10 | 247.8 | 0.685 | 0.96 (0.62, 1.84) | -0.19 | 0.85 | 0.98 |
| GDF15 (Log) | 247.8 | 0.685 | 0.93 (0.42, 1.84) | -0.18 | 0.86 | 1 |
| CASP8 | 247.8 | 0.685 | 1.05 (0.58, 1.84) | 0.17 | 0.87 | 0.99 |
| TF | 247.8 | 0.685 | 0.91 (0.31, 1.82) | -0.16 | 0.87 | 0.77 |
| IL1RL2 | 247.8 | 0.685 | 1.08 (0.43, 1.84) | 0.16 | 0.87 | 0.77 |
| FGF21 | 247.8 | 0.685 | 0.98 (0.73, 1.84) | -0.16 | 0.87 | 0.77 |
| MERTK | 247.8 | 0.685 | 1.07 (0.44, 1.83) | 0.15 | 0.88 | 0.8 |
| GP6 | 247.8 | 0.685 | 1.03 (0.68, 1.84) | 0.15 | 0.88 | 0.94 |
| IL5 | 247.8 | 0.685 | 0.97 (0.65, 1.84) | -0.15 | 0.88 | 0.99 |
| SLAMF1 | 247.8 | 0.685 | 0.95 (0.5, 1.84) | -0.15 | 0.88 | 0.95 |
| NOTCH3 | 247.8 | 0.685 | 0.93 (0.34, 1.84) | -0.14 | 0.89 | 0.92 |
| MMP12 | 247.8 | 0.685 | 1.04 (0.61, 1.82) | 0.14 | 0.89 | 0.85 |
| IL4 | 247.8 | 0.685 | 1.05 (0.57, 1.84) | 0.14 | 0.89 | 0.98 |
| IL13 | 247.8 | 0.685 | 0.97 (0.65, 1.83) | -0.14 | 0.89 | 0.98 |
| IL20 | 247.8 | 0.685 | 0.93 (0.3, 1.84) | -0.13 | 0.89 | 0.98 |
| CCL11 | 247.8 | 0.685 | 1.06 (0.43, 1.84) | 0.12 | 0.9 | 0.95 |
| LIFR | 247.9 | 0.685 | 0.94 (0.3, 1.84) | -0.1 | 0.92 | 0.97 |
| MCP4 | 247.9 | 0.685 | 0.97 (0.56, 1.82) | -0.1 | 0.92 | 0.95 |
| IL10RA | 247.9 | 0.685 | 1.02 (0.65, 1.83) | 0.09 | 0.93 | 0.96 |
| CHIT1 | 247.9 | 0.685 | 1.01 (0.75, 1.84) | 0.09 | 0.93 | 0.94 |
| PTX3 | 247.9 | 0.685 | 1.04 (0.44, 1.84) | 0.08 | 0.93 | 0.85 |
| CXCL16 | 247.9 | 0.685 | 1.06 (0.27, 1.83) | 0.08 | 0.94 | 0.93 |
| THBS2 | 247.9 | 0.685 | 1.06 (0.25, 1.84) | 0.08 | 0.94 | 0.8 |
| SCF | 247.9 | 0.685 | 1.04 (0.37, 1.84) | 0.08 | 0.94 | 0.77 |
| ANG1 | 247.9 | 0.685 | 1.01 (0.75, 1.84) | 0.08 | 0.94 | 0.38 |
| CPB1 | 247.9 | 0.685 | 1.02 (0.65, 1.83) | 0.07 | 0.95 | 0.95 |
| SPON2 | 247.9 | 0.685 | 1.07 (0.14, 1.84) | 0.07 | 0.95 | 0.77 |
| MCP2 | 247.9 | 0.685 | 1.01 (0.58, 1.83) | 0.05 | 0.96 | 0.99 |
| TNFSF13B | 247.9 | 0.685 | 1.03 (0.4, 1.84) | 0.05 | 0.96 | 0.95 |
| TNFB | 247.9 | 0.685 | 0.98 (0.43, 1.84) | -0.04 | 0.97 | 0.99 |
| SORT1 | 247.9 | 0.685 | 0.98 (0.31, 1.84) | -0.04 | 0.97 | 0.84 |
| CCL4 | 247.9 | 0.685 | 0.99 (0.63, 1.83) | -0.03 | 0.97 | 0.95 |
| IL4RA | 247.9 | 0.685 | 0.98 (0.35, 1.84) | -0.03 | 0.97 | 0.73 |
| DECR1 | 247.9 | 0.685 | 1 (0.79, 1.84) | 0.02 | 0.99 | 0.77 |
| RETN | 247.9 | 0.685 | 0.99 (0.46, 1.83) | -0.02 | 0.99 | 0.94 |
| RARRES2 | 247.9 | 0.685 | 0.99 (0.27, 1.84) | -0.01 | 0.99 | 0.95 |
| IL2 | 247.9 | 0.685 | 0 (NA, 1.84) | -0.01 | 0.99 | 0.95 |
| DKK1 | 247.9 | 0.685 | 1 (0.59, 1.83) | 0.01 | 1 | 0.85 |
| PDGFsubunitB | 247.9 | 0.685 | 1 (0.78, 1.83) | 0 | 1 | 0.77 |

Supplemental table 8- Proteins included in the logistic regression model, adjusted to the defined clinically relevant variables and medication, comparing “MI” *versus* “no CAD/no MI” participants. Full names of proteins can be found in *supplemental table 1*.

| Protein | AIC | r2 | OR (95% CI) | Z | *p*-value | FDR adjusted *p*-value |
| --- | --- | --- | --- | --- | --- | --- |
| NT3 | 233.5 | 0.754 | 3.46 (1.58, 1.93) | 3.0 | 0.003 | 0.97 |
| CTSD | 234.1 | 0.753 | 3.86 (1.61, 2.17) | 2.9 | 0.003 | 0.97 |
| AZU1 | 230.2 | 0.759 | 3.42 (1.68, 2.38) | 2.9 | 0.004 | 0.97 |
| PSPD | 234.6 | 0.752 | 2.11 (1.29, 1.87) | 2.9 | 0.004 | 0.97 |
| MPO | 235.0 | 0.751 | 11.31 (2.21, 2.35) | 2.7 | 0.006 | 0.97 |
| IL8 | 236.9 | 0.748 | 1.92 (1.17, 2.24) | 2.5 | 0.011 | 0.97 |
| HSTNT (Log) | 237.3 | 0.747 | 2.8 (1.24, 2.05) | 2.4 | 0.017 | 0.98 |
| MMP7 | 237.4 | 0.747 | 2.42 (1.19, 1.9) | 2.4 | 0.018 | 0.96 |
| OPN | 237.9 | 0.746 | 0.4 (0.18, 2.33) | -2.3 | 0.019 | 0.97 |
| PTX3 | 238.1 | 0.746 | 2.73 (1.17, 2.11) | 2.3 | 0.021 | 0.95 |
| GRN | 237.9 | 0.746 | 4.33 (1.28, 1.84) | 2.3 | 0.021 | 0.97 |
| IL12B | 238.5 | 0.745 | 0.56 (0.34, 2.21) | -2.2 | 0.025 | 0.97 |
| TSLP | 238.8 | 0.745 | 0.22 (0.04, 2.33) | -2.2 | 0.027 | 0.97 |
| TRANCE | 238.5 | 0.745 | 0.48 (0.25, 2.28) | -2.2 | 0.030 | 0.97 |
| UPA | 240.1 | 0.743 | 2.08 (0.96, 2.15) | 2.0 | 0.044 | 0.97 |
| LEP | 239.5 | 0.744 | 0.61 (0.38, 2.4) | -2.0 | 0.046 | 0.97 |
| IL5 | 239.6 | 0.743 | 0.67 (0.46, 2.3) | -2.0 | 0.047 | 0.97 |
| CCL19 | 239.6 | 0.743 | 0.65 (0.41, 2.31) | -2.0 | 0.049 | 0.97 |
| IL27 | 239.6 | 0.744 | 2.58 (1.01, 2.15) | 1.9 | 0.05 | 0.79 |
| TNFRSF13B | 239.6 | 0.744 | 0.41 (0.16, 2.26) | -1.9 | 0.05 | 0.97 |
| PRTN3 | 234.7 | 0.752 | 8.2 (1.84, 2.37) | 1.9 | 0.06 | 0.97 |
| GDF15 (Log) | 240.0 | 0.743 | 1.95 (0.97, 1.92) | 1.8 | 0.07 | 0.97 |
| TIE2 | 240.2 | 0.743 | 0.3 (0.08, 2.08) | -1.8 | 0.07 | 0.76 |
| MMP9 | 240.3 | 0.742 | 2.35 (0.93, 2.21) | 1.8 | 0.07 | 0.97 |
| KIM1 | 240.3 | 0.742 | 1.54 (0.96, 2.12) | 1.8 | 0.07 | 0.87 |
| CXCL6 | 240.3 | 0.742 | 1.4 (0.97, 2.15) | 1.8 | 0.08 | 0.97 |
| GAL9 | 240.3 | 0.742 | 0.28 (0.07, 2.28) | -1.8 | 0.08 | 0.79 |
| ADAMTS13 | 240.3 | 0.742 | 4.77 (0.87, 2.07) | 1.8 | 0.08 | 0.55 |
| AGRP | 240.4 | 0.742 | 1.86 (0.93, 2) | 1.7 | 0.08 | 0.96 |
| NTproBNP (Log) | 240.6 | 0.742 | 1.62 (0.93, 1.83) | 1.7 | 0.09 | 0.98 |
| TPA | 240.6 | 0.742 | 1.55 (0.94, 2.08) | 1.7 | 0.10 | 0.97 |
| CITP (Log) | 240.8 | 0.742 | 0.56 (0.28, 2.15) | -1.6 | 0.10 | 0.98 |
| BetaNGF | 240.9 | 0.741 | 0.41 (0.13, 2.29) | -1.6 | 0.10 | 0.97 |
| CCL16 | 240.9 | 0.741 | 1.65 (0.9, 1.94) | 1.6 | 0.11 | 0.97 |
| ADA | 240.9 | 0.741 | 1.98 (0.87, 2.02) | 1.6 | 0.11 | 0.98 |
| SLAMF7 | 240.8 | 0.741 | 0.62 (0.34, 2.29) | -1.6 | 0.11 | 0.35 |
| IL10 | 241.2 | 0.741 | 1.61 (0.87, 2.23) | 1.6 | 0.11 | 0.97 |
| CHIT1 | 241.0 | 0.741 | 0.78 (0.57, 2.14) | -1.6 | 0.12 | 0.97 |
| TRAIL | 241.0 | 0.741 | 0.34 (0.09, 2.29) | -1.6 | 0.12 | 0.97 |
| RETN | 241.0 | 0.741 | 1.83 (0.86, 2.29) | 1.6 | 0.12 | 0.97 |
| GDF2 | 241.0 | 0.741 | 2.15 (0.84, 2.21) | 1.6 | 0.12 | 0.96 |
| PIGR | 241.1 | 0.741 | 6.55 (0.6, 2.18) | 1.5 | 0.13 | 0.82 |
| CXCL10 | 241.2 | 0.741 | 0.68 (0.41, 2.28) | -1.5 | 0.13 | 0.97 |
| COL1A1 | 241.3 | 0.741 | 0.5 (0.2, 2.09) | -1.5 | 0.14 | 0.97 |
| VWF | 241.3 | 0.741 | 1.26 (0.93, 2.11) | 1.5 | 0.14 | 0.97 |
| OPG | 241.4 | 0.741 | 2.38 (0.74, 1.99) | 1.4 | 0.15 | 0.97 |
| CTSZ | 241.4 | 0.740 | 2.22 (0.75, 2.16) | 1.4 | 0.15 | 0.97 |
| CXCL9 | 241.5 | 0.740 | 0.68 (0.39, 2.27) | -1.4 | 0.15 | 0.97 |
| IL2RB | 241.4 | 0.740 | 1.79 (0.81, 2.16) | 1.4 | 0.16 | 0.97 |
| TNFSF13B | 241.5 | 0.740 | 2.12 (0.76, 2.13) | 1.4 | 0.17 | 0.97 |
| CTRC | 241.6 | 0.740 | 0.75 (0.49, 2.31) | -1.4 | 0.17 | 0.82 |
| TNFRSF10C | 241.6 | 0.740 | 1.73 (0.79, 2.1) | 1.4 | 0.17 | 0.97 |
| IL33 | 241.8 | 0.740 | 156.45 (0.25, 2.15) | 1.3 | 0.18 | 0.97 |
| BLMHYDROLASE | 241.8 | 0.740 | 1.77 (0.76, 2) | 1.3 | 0.19 | 0.97 |
| FS | 241.8 | 0.740 | 1.79 (0.76, 2.17) | 1.3 | 0.19 | 0.82 |
| LIF | 241.9 | 0.740 | 0.46 (0.14, 2.21) | -1.3 | 0.19 | 0.97 |
| MCP2 | 241.9 | 0.740 | 1.48 (0.81, 2.15) | 1.3 | 0.20 | 0.97 |
| PICP (Log) | 241.9 | 0.740 | 0.59 (0.26, 2.05) | -1.3 | 0.21 | 0.99 |
| CASP8 | 241.9 | 0.740 | 1.45 (0.81, 2.07) | 1.2 | 0.21 | 0.97 |
| TM | 241.9 | 0.740 | 0.48 (0.14, 2.18) | -1.2 | 0.21 | 0.87 |
| GDF15 (Log) | 242.0 | 0.740 | 1.67 (0.74, 1.98) | 1.2 | 0.22 | 0.98 |
| PDL1 | 242.0 | 0.740 | 0.57 (0.24, 2.3) | -1.2 | 0.22 | 0.97 |
| CD163 | 242.0 | 0.739 | 0.61 (0.27, 2.26) | -1.2 | 0.22 | 0.97 |
| SERPINA12 | 242.0 | 0.740 | 0.69 (0.37, 2.17) | -1.2 | 0.22 | 0.86 |
| GAL4 | 242.0 | 0.739 | 1.55 (0.77, 2.11) | 1.2 | 0.23 | 0.97 |
| TNFB | 242.0 | 0.739 | 0.61 (0.27, 2.21) | -1.2 | 0.23 | 0.98 |
| TNFRSF9 | 242.1 | 0.739 | 0.62 (0.28, 2.16) | -1.2 | 0.23 | 0.97 |
| FGF19 | 242.2 | 0.739 | 1.26 (0.85, 2.2) | 1.2 | 0.25 | 0.97 |
| PAI | 242.2 | 0.739 | 1.25 (0.85, 2.17) | 1.2 | 0.25 | 0.97 |
| CDCP1 | 242.2 | 0.739 | 0.71 (0.39, 2.26) | -1.2 | 0.25 | 0.97 |
| HAOX1 | 242.2 | 0.739 | 1.19 (0.89, 2.18) | 1.2 | 0.25 | 0.97 |
| IL24 | 241.5 | 0.740 | 1.9 (0.84, 2.15) | 1.1 | 0.25 | 0.97 |
| CDH5 | 242.3 | 0.739 | 0.57 (0.21, 2.17) | -1.1 | 0.27 | 0.97 |
| MMP2 | 242.3 | 0.739 | 1.86 (0.62, 2.12) | 1.1 | 0.27 | 0.97 |
| CD6 | 242.3 | 0.739 | 1.42 (0.75, 2.18) | 1.1 | 0.28 | 0.97 |
| ST2 | 242.3 | 0.739 | 1.54 (0.71, 2.09) | 1.1 | 0.29 | 0.97 |
| IL15RA | 242.4 | 0.739 | 0.46 (0.11, 2.21) | -1.1 | 0.29 | 0.97 |
| VSIG2 | 242.4 | 0.739 | 0.72 (0.39, 2.23) | -1.1 | 0.29 | 0.88 |
| MARCO | 242.4 | 0.739 | 0.4 (0.07, 2.2) | -1.0 | 0.30 | 0.97 |
| FABP4 | 242.4 | 0.739 | 0.71 (0.37, 2.29) | -1.0 | 0.30 | 0.97 |
| TIMP4 | 242.5 | 0.739 | 0.68 (0.33, 2.2) | -1.0 | 0.31 | 0.97 |
| IL17C | 242.5 | 0.739 | 1.35 (0.75, 2.15) | 1.0 | 0.31 | 0.97 |
| IL2RA | 242.5 | 0.739 | 0.73 (0.37, 2.17) | -1.0 | 0.31 | 0.97 |
| ENRAGE | 242.5 | 0.739 | 1.25 (0.81, 2.19) | 1.0 | 0.32 | 0.97 |
| IL1RT1 | 242.5 | 0.739 | 1.74 (0.58, 2.16) | 1.0 | 0.32 | 0.97 |
| PSGL1 | 242.5 | 0.739 | 1.88 (0.54, 2.03) | 1.0 | 0.32 | 0.96 |
| THBS2 | 242.6 | 0.739 | 0.45 (0.09, 2.12) | -1.0 | 0.33 | 0.87 |
| CD244 | 242.5 | 0.739 | 0.67 (0.29, 2.3) | -1.0 | 0.33 | 0.97 |
| MCP4 | 242.6 | 0.739 | 1.27 (0.77, 2.16) | 1.0 | 0.33 | 0.97 |
| ALCAM | 242.6 | 0.739 | 2.09 (0.48, 2.08) | 1.0 | 0.34 | 0.97 |
| HOSCAR | 242.6 | 0.738 | 0.49 (0.1, 2.16) | -0.9 | 0.35 | 0.97 |
| LDLRECEPTOR | 242.6 | 0.738 | 0.69 (0.31, 2.27) | -0.9 | 0.35 | 0.97 |
| TRAP | 242.6 | 0.738 | 0.59 (0.19, 2.23) | -0.9 | 0.35 | 0.97 |
| LOX1 | 242.7 | 0.738 | 1.36 (0.71, 2.16) | 0.9 | 0.36 | 0.79 |
| EPCAM | 242.7 | 0.738 | 1.22 (0.8, 2.1) | 0.9 | 0.36 | 0.97 |
| CA5A | 242.7 | 0.738 | 0.84 (0.58, 2.22) | -0.9 | 0.36 | 0.97 |
| CD4 | 242.7 | 0.738 | 0.56 (0.16, 2.2) | -0.9 | 0.36 | 0.97 |
| FABP2 | 242.7 | 0.738 | 1.23 (0.78, 2.17) | 0.9 | 0.36 | 0.96 |
| EPHB4 | 242.7 | 0.738 | 0.6 (0.2, 2.2) | -0.9 | 0.37 | 0.97 |
| IL10RB | 242.7 | 0.738 | 0.61 (0.2, 2.18) | -0.9 | 0.37 | 0.97 |
| PCSK9 | 242.7 | 0.738 | 1.59 (0.56, 2.11) | 0.9 | 0.38 | 0.97 |
| CSTB | 242.8 | 0.738 | 0.69 (0.29, 2.26) | -0.9 | 0.38 | 0.97 |
| TLT2 | 242.8 | 0.738 | 0.7 (0.31, 2.18) | -0.9 | 0.39 | 0.97 |
| IL4RA | 242.8 | 0.738 | 1.58 (0.56, 2.18) | 0.9 | 0.39 | 0.55 |
| ITGB1BP2 | 242.8 | 0.738 | 1.12 (0.86, 2.13) | 0.8 | 0.40 | 0.96 |
| CXCL1 | 242.8 | 0.738 | 1.12 (0.86, 2.18) | 0.8 | 0.40 | 0.79 |
| IL6 | 242.8 | 0.738 | 1.22 (0.76, 2.18) | 0.8 | 0.41 | 0.58 |
| TNF | 242.8 | 0.738 | 0.62 (0.22, 2.2) | -0.8 | 0.41 | 0.97 |
| DLK1 | 242.8 | 0.738 | 0.77 (0.41, 2.11) | -0.8 | 0.42 | 0.97 |
| XCL1 | 242.8 | 0.738 | 0.75 (0.36, 2.19) | -0.8 | 0.42 | 0.94 |
| ACE2 | 242.9 | 0.738 | 1.27 (0.71, 2.14) | 0.8 | 0.42 | 0.97 |
| LAPTGFbeta1 | 242.9 | 0.738 | 1.35 (0.63, 2.1) | 0.8 | 0.43 | 0.97 |
| CEACAM8 | 242.9 | 0.738 | 1.32 (0.66, 2.18) | 0.8 | 0.43 | 0.94 |
| TNFR2 | 242.9 | 0.738 | 0.72 (0.31, 2.15) | -0.8 | 0.44 | 0.97 |
| CNTN1 | 242.9 | 0.738 | 0.65 (0.21, 2.17) | -0.8 | 0.45 | 0.97 |
| PRSS8 | 242.9 | 0.738 | 1.52 (0.52, 2.09) | 0.8 | 0.45 | 0.96 |
| IL1alpha | 242.8 | 0.738 | 1.54 (0.62, 2.1) | 0.8 | 0.45 | 0.97 |
| PDGFsubunitB | 243.0 | 0.738 | 0.91 (0.7, 2.15) | -0.7 | 0.46 | 0.79 |
| UPAR | 243.0 | 0.738 | 1.48 (0.53, 2.13) | 0.7 | 0.46 | 0.97 |
| IL18BP | 243.0 | 0.738 | 0.68 (0.24, 2.17) | -0.7 | 0.46 | 0.97 |
| CASP3 | 243.0 | 0.738 | 1.1 (0.86, 2.13) | 0.7 | 0.46 | 0.97 |
| ICAM2 | 243.0 | 0.738 | 0.71 (0.28, 2.15) | -0.7 | 0.46 | 0.97 |
| MEPE | 243.0 | 0.738 | 0.69 (0.25, 2.18) | -0.7 | 0.46 | 0.97 |
| TFF3 | 243.0 | 0.738 | 0.71 (0.26, 2.22) | -0.7 | 0.49 | 0.97 |
| IL13 | 243.0 | 0.738 | 0.88 (0.6, 2.1) | -0.7 | 0.49 | 0.97 |
| IL1RA | 243.1 | 0.738 | 1.24 (0.67, 2.03) | 0.7 | 0.50 | 0.55 |
| CD5 | 243.1 | 0.738 | 1.36 (0.55, 2.16) | 0.7 | 0.51 | 0.97 |
| CD84 | 243.1 | 0.738 | 0.77 (0.36, 2.17) | -0.7 | 0.51 | 0.86 |
| GIF | 243.1 | 0.738 | 0.9 (0.65, 2.22) | -0.7 | 0.51 | 0.79 |
| LTBR | 243.1 | 0.738 | 0.69 (0.23, 2.17) | -0.7 | 0.51 | 0.97 |
| BOC | 243.1 | 0.738 | 0.65 (0.17, 2.2) | -0.6 | 0.52 | 0.55 |
| CCL23 | 243.1 | 0.738 | 0.75 (0.31, 2.16) | -0.6 | 0.52 | 0.97 |
| IL17D | 243.1 | 0.738 | 1.28 (0.53, 2.14) | 0.6 | 0.52 | 0.79 |
| PLC | 243.1 | 0.738 | 0.6 (0.12, 2.19) | -0.6 | 0.52 | 0.97 |
| BNP | 243.1 | 0.738 | 1.12 (0.8, 1.97) | 0.6 | 0.52 | 0.97 |
| ANG1 | 243.1 | 0.738 | 0.91 (0.67, 2.15) | -0.6 | 0.53 | 0.30 |
| SELP | 243.1 | 0.738 | 1.15 (0.74, 2.13) | 0.6 | 0.53 | 0.97 |
| BP14E | 243.1 | 0.738 | 1.15 (0.74, 2.01) | 0.6 | 0.53 | 0.97 |
| SCGB3A2 | 243.1 | 0.738 | 1.18 (0.7, 2.21) | 0.6 | 0.53 | 0.97 |
| IFNGAMMA | 243.3 | 0.737 | 7.34 (NA, 2.15) | 0.6 | 0.54 | 0.97 |
| JAMA | 243.1 | 0.738 | 1.11 (0.8, 2.14) | 0.6 | 0.54 | 0.97 |
| TNFSF14 | 243.1 | 0.738 | 1.2 (0.66, 2.14) | 0.6 | 0.54 | 0.97 |
| CCL25 | 243.1 | 0.738 | 1.27 (0.59, 2.22) | 0.6 | 0.54 | 0.97 |
| CPA1 | 243.1 | 0.738 | 1.15 (0.73, 2.1) | 0.6 | 0.54 | 0.97 |
| CCL15 | 243.1 | 0.738 | 1.3 (0.56, 2.1) | 0.6 | 0.54 | 0.97 |
| PARP1 | 243.2 | 0.738 | 1.13 (0.74, 2.17) | 0.6 | 0.54 | 0.97 |
| TFPI | 243.1 | 0.738 | 0.76 (0.29, 2.2) | -0.6 | 0.55 | 0.97 |
| PECAM1 | 243.1 | 0.738 | 1.15 (0.73, 2.13) | 0.6 | 0.55 | 0.97 |
| VEGFA | 243.2 | 0.738 | 1.38 (0.48, 2.08) | 0.6 | 0.55 | 0.97 |
| LPL | 243.2 | 0.738 | 0.79 (0.35, 2.06) | -0.6 | 0.56 | 0.96 |
| CHI3L1 | 243.2 | 0.738 | 1.13 (0.75, 2.16) | 0.6 | 0.57 | 0.97 |
| TNFRSF10A | 243.2 | 0.738 | 1.34 (0.48, 2.1) | 0.6 | 0.58 | 0.59 |
| CXCL11 | 243.2 | 0.737 | 1.11 (0.77, 2.1) | 0.6 | 0.58 | 0.97 |
| MMP12 | 243.2 | 0.737 | 0.85 (0.48, 2.2) | -0.6 | 0.58 | 0.97 |
| GDNF | 243.2 | 0.737 | 1.32 (0.49, 2.13) | 0.5 | 0.58 | 0.97 |
| PGLYRP1 | 243.2 | 0.737 | 0.81 (0.37, 2.15) | -0.5 | 0.58 | 0.97 |
| CD8A | 243.2 | 0.737 | 1.15 (0.69, 2.17) | 0.5 | 0.59 | 0.97 |
| AMBP | 243.2 | 0.737 | 0.57 (0.07, 2.17) | -0.5 | 0.59 | 0.90 |
| RARRES2 | 243.2 | 0.737 | 1.45 (0.36, 2.12) | 0.5 | 0.59 | 0.97 |
| GAL3 (Log) | 243.2 | 0.737 | 0.77 (0.29, 2.19) | -0.5 | 0.59 | 0.98 |
| MMP10 | 243.2 | 0.737 | 1.18 (0.64, 2.12) | 0.5 | 0.60 | 0.97 |
| PI3 | 243.2 | 0.737 | 0.83 (0.41, 2.18) | -0.5 | 0.60 | 0.97 |
| IL22RA1 | 243.2 | 0.737 | 1.31 (0.48, 2.15) | 0.5 | 0.61 | 0.97 |
| DKK1 | 243.3 | 0.737 | 0.87 (0.51, 2.17) | -0.5 | 0.61 | 0.96 |
| FGF5 | 243.3 | 0.737 | 1.82 (0.2, 2.09) | 0.5 | 0.63 | 0.97 |
| CCL11 | 243.3 | 0.737 | 0.8 (0.33, 2.17) | -0.5 | 0.63 | 0.97 |
| THPO | 243.3 | 0.737 | 1.29 (0.45, 2.16) | 0.5 | 0.63 | 0.96 |
| CX3CL1 | 243.3 | 0.737 | 0.81 (0.33, 2.18) | -0.5 | 0.65 | 0.97 |
| NOTCH3 | 243.3 | 0.737 | 1.28 (0.44, 2.16) | 0.5 | 0.65 | 0.97 |
| CXCL16 | 243.3 | 0.737 | 1.39 (0.33, 2.12) | 0.4 | 0.66 | 0.97 |
| ADM | 243.3 | 0.737 | 0.79 (0.27, 2.19) | -0.4 | 0.66 | 0.30 |
| TNFRSF11A | 243.3 | 0.737 | 1.22 (0.5, 2.11) | 0.4 | 0.66 | 0.76 |
| TNFRSF14 | 243.3 | 0.737 | 1.23 (0.48, 2.12) | 0.4 | 0.67 | 0.97 |
| ARTN | 243.3 | 0.737 | 0.8 (0.29, 2.16) | -0.4 | 0.67 | 0.97 |
| EGFR | 243.3 | 0.737 | 1.41 (0.28, 2.15) | 0.4 | 0.68 | 0.97 |
| CPB1 | 243.3 | 0.737 | 0.9 (0.55, 2.19) | -0.4 | 0.68 | 0.97 |
| IL17A | 243.3 | 0.737 | 1.12 (0.65, 2.12) | 0.4 | 0.68 | 0.97 |
| IL20RA | 243.3 | 0.737 | 1.12 (0.62, 2.12) | 0.4 | 0.68 | 0.97 |
| AXIN1 | 243.3 | 0.737 | 1.05 (0.82, 2.13) | 0.4 | 0.68 | 0.97 |
| TNFR1 | 243.3 | 0.737 | 1.24 (0.44, 2.15) | 0.4 | 0.69 | 0.97 |
| PRELP | 243.3 | 0.737 | 1.38 (0.28, 2.13) | 0.4 | 0.69 | 0.91 |
| PRSS27 | 243.4 | 0.737 | 0.85 (0.37, 2.18) | -0.4 | 0.69 | 0.76 |
| SIRT2 | 243.4 | 0.737 | 1.05 (0.81, 2.12) | 0.4 | 0.70 | 0.97 |
| CCL17 | 243.4 | 0.737 | 0.93 (0.66, 2.15) | -0.4 | 0.70 | 0.96 |
| HSP27 | 243.4 | 0.737 | 1.15 (0.55, 2.16) | 0.4 | 0.70 | 0.97 |
| IL18R1 | 243.4 | 0.737 | 0.86 (0.38, 2.18) | -0.4 | 0.70 | 0.97 |
| DECR1 | 243.4 | 0.737 | 0.95 (0.74, 2.16) | -0.4 | 0.71 | 0.87 |
| NRTN | 243.4 | 0.737 | 0.82 (0.26, 2.18) | -0.4 | 0.72 | 0.97 |
| TGFALPHA | 243.4 | 0.737 | 0.8 (0.23, 2.17) | -0.4 | 0.72 | 0.97 |
| GT | 243.4 | 0.737 | 1.09 (0.67, 2.13) | 0.4 | 0.72 | 0.97 |
| CD40 | 243.4 | 0.737 | 0.86 (0.37, 2.18) | -0.4 | 0.72 | 0.97 |
| PAPPA | 243.4 | 0.737 | 1.13 (0.58, 2.11) | 0.4 | 0.72 | 0.86 |
| SELE | 243.4 | 0.737 | 0.89 (0.47, 2.15) | -0.4 | 0.72 | 0.97 |
| MMP3 | 243.4 | 0.737 | 1.11 (0.6, 2.09) | 0.3 | 0.73 | 0.97 |
| RAGE | 243.4 | 0.737 | 1.18 (0.46, 2.14) | 0.3 | 0.73 | 0.82 |
| PIIINP (Log) | 243.4 | 0.737 | 0.9 (0.48, 2.15) | -0.3 | 0.74 | 0.99 |
| MMP1 | 243.4 | 0.737 | 0.94 (0.65, 2.14) | -0.3 | 0.74 | 0.97 |
| CSF1 | 243.4 | 0.737 | 1.31 (0.27, 2.11) | 0.3 | 0.74 | 0.98 |
| CCL24 | 243.4 | 0.737 | 1.07 (0.7, 2.15) | 0.3 | 0.75 | 0.97 |
| BMP6 | 243.4 | 0.737 | 0.88 (0.38, 2.17) | -0.3 | 0.75 | 0.30 |
| IL18 | 243.4 | 0.737 | 0.89 (0.44, 2.15) | -0.3 | 0.75 | 0.82 |
| FAS | 243.4 | 0.737 | 1.19 (0.42, 2.15) | 0.3 | 0.76 | 0.97 |
| HBEGF | 243.4 | 0.737 | 0.92 (0.55, 2.16) | -0.3 | 0.76 | 0.96 |
| CCL20 | 243.4 | 0.737 | 1.06 (0.71, 2.17) | 0.3 | 0.76 | 0.97 |
| IL17RA | 243.4 | 0.737 | 0.9 (0.47, 2.17) | -0.3 | 0.76 | 0.97 |
| MCP1 | 243.4 | 0.737 | 1.14 (0.55, 2.13) | 0.3 | 0.76 | 0.97 |
| GLO1 | 243.4 | 0.737 | 0.93 (0.57, 2.17) | -0.3 | 0.77 | 0.82 |
| CST5 | 243.4 | 0.737 | 0.89 (0.42, 2.14) | -0.3 | 0.77 | 0.97 |
| HGF | 243.4 | 0.737 | 1.12 (0.48, 2.13) | 0.3 | 0.78 | 0.97 |
| VEGFD | 243.4 | 0.737 | 0.87 (0.32, 2.17) | -0.3 | 0.78 | 0.97 |
| CCL28 | 243.4 | 0.737 | 0.86 (0.29, 2.16) | -0.3 | 0.78 | 0.97 |
| SHPS1 | 243.4 | 0.737 | 1.13 (0.46, 2.15) | 0.3 | 0.78 | 0.97 |
| GH | 243.4 | 0.737 | 1.03 (0.84, 2.16) | 0.3 | 0.79 | 0.82 |
| IGFBP2 | 243.4 | 0.737 | 1.1 (0.55, 2.16) | 0.3 | 0.79 | 0.97 |
| STAMBP | 243.4 | 0.737 | 1.05 (0.74, 2.12) | 0.3 | 0.80 | 0.97 |
| STK4 | 243.5 | 0.737 | 1.04 (0.77, 2.16) | 0.2 | 0.81 | 0.60 |
| MB | 243.5 | 0.737 | 1.08 (0.54, 2.13) | 0.2 | 0.82 | 0.97 |
| TR | 243.5 | 0.737 | 0.94 (0.54, 2.16) | -0.2 | 0.83 | 0.97 |
| FGF23 | 243.5 | 0.737 | 0.93 (0.5, 2.16) | -0.2 | 0.83 | 0.82 |
| IL6RA | 243.5 | 0.737 | 1.11 (0.41, 2.16) | 0.2 | 0.83 | 0.97 |
| TRAILR2 | 243.5 | 0.737 | 1.11 (0.41, 2.14) | 0.2 | 0.83 | 0.76 |
| SLAMF1 | 243.5 | 0.737 | 0.93 (0.49, 2.16) | -0.2 | 0.83 | 0.97 |
| CCL3 | 243.5 | 0.737 | 1.06 (0.6, 2.15) | 0.2 | 0.83 | 0.96 |
| TGM2 | 243.5 | 0.737 | 1.05 (0.69, 2.15) | 0.2 | 0.84 | 0.97 |
| PGF | 243.5 | 0.737 | 0.89 (0.28, 2.16) | -0.2 | 0.84 | 0.52 |
| SORT1 | 243.5 | 0.737 | 1.13 (0.34, 2.15) | 0.2 | 0.84 | 0.94 |
| IGFBP7 | 243.5 | 0.737 | 1.14 (0.34, 2.12) | 0.2 | 0.84 | 0.97 |
| IL7 | 243.5 | 0.737 | 1.05 (0.65, 2.11) | 0.2 | 0.84 | 0.97 |
| OSM | 243.5 | 0.737 | 1.05 (0.66, 2.14) | 0.2 | 0.84 | 0.97 |
| SRC | 243.5 | 0.737 | 1.03 (0.74, 2.16) | 0.2 | 0.85 | 0.55 |
| DNER | 243.5 | 0.737 | 0.9 (0.27, 2.16) | -0.2 | 0.86 | 0.97 |
| IL10RA | 243.5 | 0.737 | 0.95 (0.52, 2.16) | -0.2 | 0.86 | 0.97 |
| HO1 | 243.5 | 0.737 | 0.92 (0.36, 2.14) | -0.2 | 0.86 | 0.91 |
| KLK6 | 243.5 | 0.737 | 1.08 (0.47, 2.15) | 0.2 | 0.86 | 0.97 |
| PDGFsubunitA | 243.5 | 0.737 | 0.97 (0.68, 2.16) | -0.2 | 0.86 | 0.97 |
| SOD2 | 243.5 | 0.737 | 0.79 (0.05, 2.16) | -0.2 | 0.87 | 0.82 |
| CXCL5 | 243.5 | 0.737 | 1.02 (0.82, 2.15) | 0.2 | 0.87 | 0.97 |
| DCN | 243.5 | 0.737 | 0.92 (0.32, 2.16) | -0.2 | 0.87 | 0.96 |
| PDL2 | 243.5 | 0.737 | 1.09 (0.38, 2.15) | 0.2 | 0.88 | 0.97 |
| CTSL1 | 243.5 | 0.737 | 1.08 (0.4, 2.15) | 0.1 | 0.88 | 0.97 |
| IL20 | 243.5 | 0.737 | 0.93 (0.3, 2.15) | -0.1 | 0.89 | 0.97 |
| CD93 | 243.5 | 0.737 | 1.09 (0.32, 2.16) | 0.1 | 0.89 | 0.97 |
| PAR1 | 243.5 | 0.737 | 0.94 (0.38, 2.15) | -0.1 | 0.89 | 0.76 |
| GP6 | 243.5 | 0.737 | 0.97 (0.62, 2.16) | -0.1 | 0.89 | 0.97 |
| IL16 | 243.5 | 0.737 | 1.05 (0.51, 2.16) | 0.1 | 0.89 | 0.94 |
| APN | 243.5 | 0.737 | 0.93 (0.3, 2.16) | -0.1 | 0.89 | 0.97 |
| IL2 | 243.3 | 0.737 | 1.2E12(0, 2.15) | 0.1 | 0.89 | 0.97 |
| MCP3 | 243.5 | 0.737 | 0.96 (0.53, 2.16) | -0.1 | 0.90 | 0.97 |
| CCL4 | 243.5 | 0.737 | 0.96 (0.54, 2.16) | -0.1 | 0.90 | 0.97 |
| ITGB2 | 243.5 | 0.737 | 1.05 (0.46, 2.16) | 0.1 | 0.90 | 0.97 |
| ST1A1 | 243.5 | 0.737 | 1.03 (0.68, 2.15) | 0.1 | 0.91 | 0.97 |
| REN | 243.5 | 0.737 | 0.98 (0.63, 2.15) | -0.1 | 0.91 | 0.86 |
| MERTK | 243.5 | 0.737 | 1.05 (0.44, 2.16) | 0.1 | 0.91 | 0.87 |
| CD40L | 243.5 | 0.737 | 0.99 (0.76, 2.16) | -0.1 | 0.92 | 0.30 |
| IL1RL2 | 243.5 | 0.737 | 0.95 (0.36, 2.15) | -0.1 | 0.92 | 0.79 |
| PON3 | 243.5 | 0.737 | 0.96 (0.46, 2.16) | -0.1 | 0.92 | 0.97 |
| AXL | 243.5 | 0.737 | 1.06 (0.33, 2.15) | 0.1 | 0.92 | 0.97 |
| FGF21 | 243.5 | 0.737 | 0.99 (0.73, 2.15) | -0.1 | 0.92 | 0.82 |
| IDUA | 243.5 | 0.737 | 1.03 (0.44, 2.14) | 0.1 | 0.94 | 0.76 |
| LIFR | 243.5 | 0.737 | 0.95 (0.29, 2.16) | -0.1 | 0.94 | 0.97 |
| TWEAK | 243.5 | 0.737 | 1.04 (0.4, 2.14) | 0.1 | 0.94 | 0.97 |
| IL1RT2 | 243.5 | 0.737 | 0.96 (0.33, 2.16) | -0.1 | 0.95 | 0.97 |
| TF | 243.5 | 0.737 | 1.04 (0.31, 2.15) | 0.1 | 0.95 | 0.78 |
| IGFBP1 | 243.5 | 0.737 | 1.01 (0.7, 2.15) | 0.1 | 0.95 | 0.97 |
| NEMO | 243.5 | 0.737 | 1.01 (0.77, 2.15) | 0.1 | 0.96 | 0.97 |
| IgGFcreceptorIlb | 243.5 | 0.737 | 1.01 (0.64, 2.16) | 0.0 | 0.96 | 0.96 |
| SPON2 | 243.5 | 0.737 | 0.96 (0.14, 2.15) | 0.0 | 0.97 | 0.82 |
| FLT3L | 243.5 | 0.737 | 0.99 (0.45, 2.15) | 0.0 | 0.97 | 0.97 |
| SPON1 | 243.5 | 0.737 | 0.98 (0.32, 2.15) | 0.0 | 0.97 | 0.97 |
| IL4 | 243.5 | 0.737 | 0.99 (0.51, 2.15) | 0.0 | 0.99 | 0.97 |
| SCF | 243.5 | 0.737 | 1 (0.35, 2.15) | 0.0 | 0.99 | 0.82 |

Supplemental table 9- Proteins included in the logistic regression model, adjusted to the defined clinically relevant variables and medication, comparing “MI” *versus* “CAD” participants. Full names of proteins can be found in *supplemental table 1*.

| Protein | AIC | *r*^2^ | OR (95% CI) | Z | *p*-value | FDR adjusted *p*-value |
| --- | --- | --- | --- | --- | --- | --- |
| MMP9 | 508.9 | 0.166 | 2.55 (1.35, 0.75) | 2.8 | 0.005 | 0.91 |
| UPAR | 511.3 | 0.159 | 2.21 (1.18, 0.75) | 2.4 | 0.015 | 0.92 |
| PARP1 | 511.2 | 0.159 | 1.49 (1.09, 0.71) | 2.4 | 0.018 | 0.91 |
| GDF15 (Log) | 511.8 | 0.157 | 1.7 (1.1, 0.68) | 2.3 | 0.019 | 0.91 |
| IL2RA | 512.1 | 0.156 | 1.86 (1.1, 0.79) | 2.3 | 0.022 | 0.91 |
| IGFBP2 | 512.4 | 0.155 | 1.63 (1.06, 0.78) | 2.2 | 0.026 | 0.92 |
| CCL16 | 512.6 | 0.155 | 1.5 (1.05, 0.69) | 2.2 | 0.030 | 0.92 |
| TRAILR2 | 512.6 | 0.155 | 2 (1.08, 0.73) | 2.2 | 0.031 | 0.74 |
| CHI3L1 | 512.8 | 0.154 | 1.31 (1.02, 0.74) | 2.1 | 0.033 | 0.92 |
| PIGR | 513.1 | 0.153 | 4.24 (1.09, 0.71) | 2.1 | 0.040 | 0.78 |
| MB | 513.2 | 0.153 | 1.51 (1.02, 0.73) | 2.0 | 0.044 | 0.92 |
| PTX3 | 513.3 | 0.152 | 1.7 (1.02, 0.7) | 2.0 | 0.045 | 0.88 |
| KIM1 | 513.4 | 0.152 | 1.37 (1.01, 0.68) | 2.0 | 0.047 | 0.88 |
| IL16 | 513.4 | 0.152 | 1.62 (1.01, 0.72) | 2.0 | 0.048 | 0.88 |
| CD93 | 513.6 | 0.152 | 2.08 (1, 0.77) | 1.9 | 0.05 | 0.92 |
| CTSZ | 513.6 | 0.151 | 1.77 (1, 0.72) | 1.9 | 0.05 | 0.92 |
| CD8A | 513.6 | 0.151 | 1.39 (1, 0.71) | 1.9 | 0.05 | 0.92 |
| TNFRSF9 | 513.7 | 0.151 | 1.69 (0.99, 0.77) | 1.9 | 0.06 | 0.97 |
| IL1RA | 513.7 | 0.151 | 1.5 (0.99, 0.67) | 1.9 | 0.06 | 0.74 |
| TNFR1 | 513.8 | 0.151 | 1.92 (0.99, 0.75) | 1.9 | 0.06 | 0.92 |
| OSM | 513.8 | 0.151 | 1.29 (0.99, 0.71) | 1.9 | 0.06 | 0.92 |
| PAI | 513.9 | 0.151 | 0.8 (0.64, 0.73) | -1.9 | 0.06 | 0.91 |
| TGFALPHA | 513.9 | 0.151 | 1.98 (0.97, 0.71) | 1.9 | 0.06 | 0.95 |
| TGM2 | 514.0 | 0.150 | 1.3 (0.99, 0.71) | 1.9 | 0.06 | 0.91 |
| GDF15 (Log) | 514.1 | 0.150 | 1.68 (0.97, 0.67) | 1.8 | 0.07 | 0.98 |
| IL18BP | 514.2 | 0.150 | 1.79 (0.95, 0.73) | 1.8 | 0.07 | 0.92 |
| MMP7 | 514.3 | 0.149 | 0.61 (0.35, 0.72) | -1.8 | 0.08 | 0.88 |
| MMP1 | 514.3 | 0.149 | 1.21 (0.98, 0.73) | 1.7 | 0.08 | 0.95 |
| CST5 | 514.4 | 0.149 | 1.52 (0.95, 0.75) | 1.7 | 0.08 | 0.92 |
| NTproBNP (Log) | 514.5 | 0.149 | 1.37 (0.96, 0.7) | 1.7 | 0.09 | 0.98 |
| LIF | 513.8 | 0.151 | 0.51 (0.21, 0.69) | -1.7 | 0.09 | 0.97 |
| LEP | 514.5 | 0.149 | 0.76 (0.56, 0.76) | -1.7 | 0.09 | 0.91 |
| MMP10 | 514.6 | 0.149 | 1.42 (0.95, 0.75) | 1.7 | 0.09 | 0.95 |
| IL7 | 514.7 | 0.148 | 0.79 (0.59, 0.71) | -1.7 | 0.10 | 0.92 |
| CD5 | 514.7 | 0.148 | 1.6 (0.92, 0.72) | 1.6 | 0.10 | 0.95 |
| HSTNT (Log) | 514.7 | 0.148 | 1.52 (0.93, 0.72) | 1.6 | 0.10 | 0.98 |
| LOX1 | 514.8 | 0.148 | 1.39 (0.94, 0.72) | 1.6 | 0.11 | 0.78 |
| ADA | 514.8 | 0.148 | 1.53 (0.91, 0.72) | 1.6 | 0.11 | 0.98 |
| NT3 | 514.9 | 0.148 | 1.37 (0.93, 0.72) | 1.6 | 0.11 | 0.97 |
| CPB1 | 515.0 | 0.147 | 0.78 (0.56, 0.75) | -1.6 | 0.12 | 0.92 |
| CA5A | 515.2 | 0.147 | 1.23 (0.94, 0.69) | 1.5 | 0.13 | 0.91 |
| IL6RA | 515.3 | 0.146 | 1.5 (0.87, 0.71) | 1.5 | 0.14 | 0.91 |
| CTSL1 | 515.3 | 0.146 | 1.62 (0.85, 0.73) | 1.5 | 0.15 | 0.91 |
| TNFRSF10C | 515.3 | 0.146 | 1.41 (0.89, 0.7) | 1.5 | 0.15 | 0.91 |
| CHIT1 | 515.3 | 0.146 | 0.87 (0.71, 0.69) | -1.4 | 0.15 | 0.91 |
| TNFR2 | 515.4 | 0.146 | 1.53 (0.86, 0.73) | 1.4 | 0.15 | 0.91 |
| TWEAK | 515.4 | 0.146 | 0.62 (0.32, 0.69) | -1.4 | 0.15 | 0.97 |
| GAL4 | 515.5 | 0.146 | 0.75 (0.49, 0.72) | -1.4 | 0.16 | 0.92 |
| PECAM1 | 515.6 | 0.145 | 0.83 (0.63, 0.72) | -1.4 | 0.17 | 0.92 |
| CEACAM8 | 515.5 | 0.145 | 1.31 (0.89, 0.72) | 1.4 | 0.17 | 0.88 |
| GAL3 (Log) | 515.6 | 0.145 | 1.51 (0.84, 0.71 ) | 1.4 | 0.17 | 0.98 |
| TNFRSF11A | 515.6 | 0.145 | 1.5 (0.84, 0.71) | 1.4 | 0.17 | 0.74 |
| CXCL5 | 515.6 | 0.145 | 0.92 (0.8, 0.71) | -1.4 | 0.18 | 0.95 |
| CCL19 | 515.7 | 0.145 | 0.84 (0.64, 0.69) | -1.3 | 0.18 | 0.95 |
| PI3 | 515.7 | 0.145 | 1.32 (0.88, 0.7) | 1.3 | 0.19 | 0.91 |
| CCL25 | 515.7 | 0.145 | 1.37 (0.86, 0.74) | 1.3 | 0.19 | 0.97 |
| SERPINA12 | 515.6 | 0.145 | 0.78 (0.54, 0.71) | -1.3 | 0.19 | 0.88 |
| CNTN1 | 515.7 | 0.145 | 0.65 (0.34, 0.7) | -1.3 | 0.19 | 0.91 |
| IL18R1 | 515.8 | 0.145 | 0.71 (0.42, 0.71) | -1.3 | 0.20 | 0.95 |
| ITGB2 | 515.8 | 0.145 | 1.4 (0.84, 0.71) | 1.3 | 0.20 | 0.91 |
| IL10RB | 515.8 | 0.145 | 1.54 (0.8, 0.72) | 1.3 | 0.20 | 0.95 |
| GH | 515.8 | 0.145 | 0.93 (0.82, 0.72) | -1.3 | 0.20 | 0.80 |
| IL10RA | 515.8 | 0.145 | 0.81 (0.57, 0.7) | -1.3 | 0.20 | 0.95 |
| HGF | 515.8 | 0.145 | 1.45 (0.82, 0.71) | 1.3 | 0.20 | 0.95 |
| CASP3 | 515.9 | 0.144 | 0.91 (0.78, 0.72) | -1.2 | 0.21 | 0.92 |
| GIF | 515.9 | 0.144 | 0.88 (0.72, 0.73) | -1.2 | 0.21 | 0.78 |
| IL18 | 515.9 | 0.144 | 1.31 (0.85, 0.7) | 1.2 | 0.22 | 0.78 |
| AXL | 516.0 | 0.144 | 1.47 (0.79, 0.73) | 1.2 | 0.23 | 0.91 |
| CTRC | 516.0 | 0.144 | 0.85 (0.64, 0.72) | -1.2 | 0.24 | 0.80 |
| TNFSF13B | 516.0 | 0.144 | 1.45 (0.79, 0.73) | 1.2 | 0.24 | 0.92 |
| IL1RL2 | 516.1 | 0.144 | 0.72 (0.41, 0.69) | -1.2 | 0.24 | 0.74 |
| IL1RT2 | 516.1 | 0.144 | 0.68 (0.36, 0.7) | -1.2 | 0.24 | 0.92 |
| CTSD | 516.1 | 0.144 | 1.35 (0.82, 0.69) | 1.2 | 0.24 | 0.92 |
| ENRAGE | 516.1 | 0.144 | 1.18 (0.9, 0.72) | 1.2 | 0.24 | 0.96 |
| GDNF | 516.1 | 0.144 | 0.71 (0.4, 0.72) | -1.2 | 0.25 | 0.92 |
| TF | 516.1 | 0.144 | 1.51 (0.75, 0.73) | 1.1 | 0.25 | 0.74 |
| JAMA | 516.2 | 0.143 | 0.89 (0.74, 0.72) | -1.1 | 0.26 | 0.92 |
| FAS | 516.2 | 0.143 | 1.35 (0.81, 0.72) | 1.1 | 0.27 | 0.91 |
| IGFBP1 | 516.2 | 0.143 | 1.15 (0.89, 0.74) | 1.1 | 0.27 | 0.91 |
| IL4RA | 516.2 | 0.143 | 1.45 (0.75, 0.72) | 1.1 | 0.27 | 0.74 |
| AZU1 | 516.3 | 0.143 | 1.2 (0.87, 0.72) | 1.1 | 0.28 | 0.91 |
| TLT2 | 516.3 | 0.143 | 1.27 (0.83, 0.71) | 1.1 | 0.28 | 0.91 |
| OPG | 516.3 | 0.143 | 1.43 (0.75, 0.72) | 1.1 | 0.28 | 0.91 |
| ADAMTS13 | 516.3 | 0.143 | 0.59 (0.22, 0.72) | -1.1 | 0.29 | 0.74 |
| TRAIL | 516.4 | 0.143 | 0.67 (0.31, 0.7) | -1.0 | 0.30 | 0.92 |
| IL22RA1 | 516.3 | 0.143 | 1.46 (0.74, 0.72) | 1.0 | 0.30 | 0.95 |
| IL24 | 516.3 | 0.143 | 1.23 (0.85, 0.69) | 1.0 | 0.30 | 0.95 |
| GLO1 | 516.4 | 0.143 | 0.85 (0.62, 0.72) | -1.0 | 0.31 | 0.88 |
| PAPPA | 516.4 | 0.143 | 1.24 (0.82, 0.73) | 1.0 | 0.31 | 0.88 |
| BOC | 516.4 | 0.143 | 1.54 (0.68, 0.71) | 1.0 | 0.31 | 0.74 |
| HO1 | 516.4 | 0.143 | 1.35 (0.76, 0.72) | 1.0 | 0.31 | 0.88 |
| LPL | 516.4 | 0.143 | 0.79 (0.5, 0.67) | -1.0 | 0.31 | 0.90 |
| PDGFsubunitA | 516.4 | 0.143 | 0.9 (0.74, 0.71) | -1.0 | 0.31 | 0.92 |
| PSGL1 | 516.5 | 0.143 | 1.52 (0.67, 0.68) | 1.0 | 0.32 | 0.88 |
| AGRP | 516.5 | 0.142 | 1.25 (0.81, 0.7) | 1.0 | 0.32 | 0.91 |
| NRTN | 516.5 | 0.142 | 0.76 (0.43, 0.72) | -1.0 | 0.33 | 0.97 |
| IL15RA | 516.5 | 0.142 | 0.66 (0.28, 0.7) | -1.0 | 0.34 | 0.95 |
| PGLYRP1 | 516.5 | 0.142 | 1.26 (0.78, 0.73) | 1.0 | 0.34 | 0.92 |
| HSP27 | 516.5 | 0.142 | 0.79 (0.48, 0.71) | -0.9 | 0.34 | 0.91 |
| IL10 | 516.5 | 0.142 | 0.85 (0.6, 0.68) | -0.9 | 0.35 | 0.95 |
| PLC | 516.6 | 0.142 | 1.48 (0.65, 0.73) | 0.9 | 0.35 | 0.91 |
| RETN | 516.6 | 0.142 | 1.22 (0.8, 0.73) | 0.9 | 0.36 | 0.91 |
| ALCAM | 516.6 | 0.142 | 1.48 (0.64, 0.72) | 0.9 | 0.36 | 0.91 |
| CXCL10 | 516.6 | 0.142 | 0.86 (0.63, 0.69) | -0.9 | 0.36 | 0.95 |
| OPN | 516.7 | 0.142 | 1.25 (0.77, 0.72) | 0.9 | 0.37 | 0.92 |
| ICAM2 | 516.7 | 0.142 | 0.79 (0.46, 0.7) | -0.9 | 0.37 | 0.92 |
| SLAMF7 | 516.7 | 0.142 | 0.86 (0.62, 0.7) | -0.9 | 0.38 | 0.74 |
| TRAP | 516.7 | 0.142 | 1.32 (0.72, 0.72) | 0.9 | 0.38 | 0.91 |
| IL12B | 516.7 | 0.142 | 0.86 (0.6, 0.7) | -0.9 | 0.38 | 0.95 |
| CASP8 | 516.7 | 0.142 | 1.18 (0.82, 0.71) | 0.9 | 0.38 | 0.97 |
| PRTN3 | 516.7 | 0.142 | 1.4 (0.64, 0.72) | 0.9 | 0.38 | 0.92 |
| CPA1 | 516.7 | 0.142 | 0.88 (0.66, 0.72) | -0.9 | 0.38 | 0.92 |
| TSLP | 516.6 | 0.142 | 0.66 (0.2, 0.68) | -0.9 | 0.39 | 0.92 |
| EPHB4 | 516.7 | 0.142 | 1.36 (0.67, 0.72) | 0.9 | 0.39 | 0.91 |
| CSF1 | 516.7 | 0.142 | 1.5 (0.6, 0.72) | 0.9 | 0.39 | 0.98 |
| REN | 516.7 | 0.142 | 0.89 (0.68, 0.72) | -0.9 | 0.39 | 0.88 |
| CCL20 | 516.7 | 0.142 | 1.1 (0.88, 0.72) | 0.9 | 0.39 | 0.97 |
| TIMP4 | 516.7 | 0.142 | 0.81 (0.49, 0.7) | -0.9 | 0.39 | 0.91 |
| IL1RT1 | 516.8 | 0.142 | 1.31 (0.69, 0.72) | 0.8 | 0.41 | 0.91 |
| IL20 | 516.7 | 0.142 | 0.69 (0.26, 0.72) | -0.8 | 0.41 | 0.95 |
| SRC | 516.8 | 0.142 | 0.91 (0.72, 0.71) | -0.8 | 0.41 | 0.74 |
| TNFRSF14 | 516.8 | 0.142 | 1.23 (0.75, 0.71) | 0.8 | 0.41 | 0.91 |
| BetaNGF | 516.8 | 0.142 | 1.37 (0.66, 0.72) | 0.8 | 0.41 | 0.95 |
| PDL1 | 516.8 | 0.141 | 1.26 (0.72, 0.71) | 0.8 | 0.42 | 0.95 |
| THBS2 | 516.8 | 0.141 | 0.68 (0.26, 0.71) | -0.8 | 0.42 | 0.88 |
| HOSCAR | 516.8 | 0.141 | 1.41 (0.6, 0.72) | 0.8 | 0.43 | 0.91 |
| TR | 516.8 | 0.141 | 1.15 (0.81, 0.7) | 0.8 | 0.44 | 0.91 |
| MCP4 | 516.8 | 0.141 | 1.13 (0.83, 0.72) | 0.8 | 0.44 | 0.95 |
| PRSS8 | 516.9 | 0.141 | 1.32 (0.65, 0.68) | 0.8 | 0.44 | 0.91 |
| IL4 | 516.8 | 0.141 | 1.19 (0.78, 0.71) | 0.8 | 0.44 | 0.96 |
| PRELP | 516.9 | 0.141 | 0.66 (0.23, 0.72) | -0.8 | 0.44 | 0.88 |
| IL20RA | 516.8 | 0.141 | 1.22 (0.75, 0.7) | 0.8 | 0.44 | 0.92 |
| UPA | 516.9 | 0.141 | 1.22 (0.73, 0.72) | 0.8 | 0.44 | 0.92 |
| AMBP | 516.9 | 0.141 | 1.56 (0.5, 0.7) | 0.8 | 0.45 | 0.88 |
| IL6 | 516.9 | 0.141 | 1.1 (0.86, 0.71) | 0.8 | 0.45 | 0.74 |
| TNFSF14 | 516.9 | 0.141 | 1.16 (0.79, 0.71) | 0.8 | 0.45 | 0.95 |
| ST1A1 | 516.9 | 0.141 | 0.9 (0.69, 0.71) | -0.7 | 0.46 | 0.97 |
| MMP2 | 516.9 | 0.141 | 1.28 (0.67, 0.72) | 0.7 | 0.46 | 0.91 |
| THPO | 516.9 | 0.141 | 0.79 (0.43, 0.71) | -0.7 | 0.46 | 0.91 |
| TFF3 | 516.9 | 0.141 | 1.26 (0.69, 0.71) | 0.7 | 0.46 | 0.91 |
| MCP1 | 516.9 | 0.141 | 0.88 (0.6, 0.69) | -0.7 | 0.46 | 0.91 |
| FGF23 | 516.9 | 0.141 | 0.86 (0.58, 0.71) | -0.7 | 0.47 | 0.80 |
| CCL15 | 517.0 | 0.141 | 1.21 (0.72, 0.71) | 0.7 | 0.48 | 0.92 |
| PIIINP (Log) | 517.0 | 0.141 | 0.86 (0.57, 0.7) | -0.7 | 0.48 | 1.00 |
| ST2 | 517.0 | 0.141 | 0.85 (0.54, 0.7) | -0.7 | 0.48 | 0.92 |
| SELP | 517.0 | 0.141 | 0.91 (0.7, 0.71) | -0.7 | 0.48 | 0.91 |
| IL33 | 516.9 | 0.141 | 2.05 (0.39, 0.7) | 0.7 | 0.49 | 0.96 |
| SPON2 | 517.0 | 0.141 | 1.5 (0.47, 0.71) | 0.7 | 0.49 | 0.80 |
| FABP2 | 517.0 | 0.141 | 1.1 (0.83, 0.71) | 0.7 | 0.50 | 0.91 |
| SHPS1 | 517.0 | 0.141 | 1.21 (0.7, 0.71) | 0.7 | 0.50 | 0.92 |
| IDUA | 517.0 | 0.141 | 1.18 (0.72, 0.69) | 0.7 | 0.50 | 0.74 |
| PCSK9 | 517.0 | 0.141 | 0.82 (0.44, 0.72) | -0.7 | 0.52 | 0.92 |
| MEPE | 517.0 | 0.141 | 0.83 (0.47, 0.71) | -0.6 | 0.52 | 0.92 |
| ITGB1BP2 | 517.1 | 0.141 | 0.95 (0.81, 0.71) | -0.6 | 0.53 | 0.88 |
| LIFR | 517.1 | 0.141 | 0.79 (0.38, 0.7) | -0.6 | 0.53 | 0.95 |
| CSTB | 517.1 | 0.141 | 1.17 (0.72, 0.71) | 0.6 | 0.53 | 0.91 |
| CCL23 | 517.1 | 0.141 | 0.85 (0.52, 0.7) | -0.6 | 0.53 | 0.95 |
| STK4 | 517.1 | 0.141 | 0.94 (0.78, 0.71) | -0.6 | 0.53 | 0.74 |
| VWF | 517.1 | 0.141 | 1.05 (0.89, 0.71) | 0.6 | 0.53 | 0.92 |
| TFPI | 517.1 | 0.141 | 0.82 (0.43, 0.71) | -0.6 | 0.54 | 0.91 |
| PSPD | 517.1 | 0.141 | 1.09 (0.83, 0.7) | 0.6 | 0.54 | 0.91 |
| LTBR | 517.1 | 0.141 | 1.24 (0.62, 0.72) | 0.6 | 0.54 | 0.91 |
| SOD2 | 517.1 | 0.141 | 1.69 (0.3, 0.7) | 0.6 | 0.55 | 0.80 |
| ADM | 517.1 | 0.140 | 0.81 (0.4, 0.7) | -0.6 | 0.55 | 0.74 |
| ACE2 | 517.1 | 0.140 | 1.12 (0.77, 0.7) | 0.6 | 0.56 | 0.91 |
| EPCAM | 517.1 | 0.140 | 0.93 (0.74, 0.7) | -0.6 | 0.56 | 0.91 |
| VSIG2 | 517.1 | 0.140 | 1.11 (0.78, 0.7) | 0.6 | 0.56 | 0.88 |
| IL27 | 517.1 | 0.140 | 0.84 (0.47, 0.7) | -0.6 | 0.56 | 0.74 |
| IGFBP7 | 517.1 | 0.140 | 1.2 (0.64, 0.71) | 0.6 | 0.57 | 0.92 |
| CCL4 | 517.1 | 0.140 | 0.92 (0.67, 0.7) | -0.6 | 0.57 | 0.92 |
| IL5 | 517.1 | 0.140 | 0.94 (0.76, 0.72) | -0.6 | 0.58 | 0.98 |
| FGF19 | 517.1 | 0.140 | 1.07 (0.84, 0.7) | 0.6 | 0.58 | 0.96 |
| GT | 517.2 | 0.140 | 1.09 (0.8, 0.71) | 0.5 | 0.59 | 0.91 |
| FABP4 | 517.2 | 0.140 | 1.1 (0.76, 0.7) | 0.5 | 0.60 | 0.91 |
| TNF | 517.2 | 0.140 | 1.26 (0.59, 0.7) | 0.5 | 0.60 | 0.95 |
| FLT3L | 517.2 | 0.140 | 0.87 (0.52, 0.7) | -0.5 | 0.60 | 0.95 |
| STAMBP | 517.2 | 0.140 | 0.94 (0.76, 0.71) | -0.5 | 0.60 | 0.97 |
| CCL24 | 517.2 | 0.140 | 1.07 (0.84, 0.7) | 0.5 | 0.60 | 0.91 |
| RARRES2 | 517.2 | 0.140 | 1.19 (0.59, 0.71) | 0.5 | 0.62 | 0.92 |
| SLAMF1 | 517.2 | 0.140 | 1.11 (0.74, 0.71) | 0.5 | 0.62 | 0.93 |
| PRSS27 | 517.2 | 0.140 | 1.12 (0.71, 0.71) | 0.5 | 0.63 | 0.74 |
| VEGFD | 517.2 | 0.140 | 1.15 (0.65, 0.71) | 0.5 | 0.63 | 0.91 |
| PGF | 517.2 | 0.140 | 1.2 (0.58, 0.72) | 0.5 | 0.63 | 0.74 |
| KLK6 | 517.2 | 0.140 | 1.14 (0.66, 0.71) | 0.5 | 0.63 | 0.92 |
| HBEGF | 517.2 | 0.140 | 1.08 (0.79, 0.71) | 0.5 | 0.64 | 0.91 |
| MPO | 517.2 | 0.140 | 1.21 (0.54, 0.71) | 0.5 | 0.64 | 0.91 |
| DNER | 517.2 | 0.140 | 0.83 (0.38, 0.71) | -0.5 | 0.64 | 0.96 |
| IL1alpha | 517.2 | 0.140 | 1.35 (0.53, 0.71) | 0.5 | 0.64 | 0.92 |
| TNFRSF13B | 517.2 | 0.140 | 1.12 (0.68, 0.71) | 0.5 | 0.64 | 0.91 |
| IL13 | 517.2 | 0.140 | 1.07 (0.82, 0.71) | 0.5 | 0.64 | 0.95 |
| GAL9 | 517.3 | 0.140 | 1.19 (0.56, 0.71) | 0.5 | 0.65 | 0.78 |
| MARCO | 517.3 | 0.140 | 1.26 (0.45, 0.7) | 0.4 | 0.66 | 0.91 |
| PAR1 | 517.3 | 0.140 | 1.11 (0.68, 0.7) | 0.4 | 0.67 | 0.74 |
| TPA | 517.3 | 0.140 | 0.94 (0.72, 0.7) | -0.4 | 0.67 | 0.92 |
| SCGB3A2 | 517.3 | 0.140 | 1.07 (0.78, 0.71) | 0.4 | 0.67 | 0.92 |
| MCP3 | 517.3 | 0.140 | 1.09 (0.73, 0.7) | 0.4 | 0.67 | 0.92 |
| CX3CL1 | 517.3 | 0.140 | 0.89 (0.53, 0.69) | -0.4 | 0.68 | 0.97 |
| DCN | 517.3 | 0.140 | 0.87 (0.45, 0.7) | -0.4 | 0.69 | 0.90 |
| FGF5 | 517.3 | 0.140 | 0.77 (0.20, 0.7) | -0.4 | 0.69 | 0.95 |
| CD40L | 517.3 | 0.140 | 0.97 (0.83, 0.71) | -0.4 | 0.69 | 0.74 |
| SCF | 517.3 | 0.140 | 0.88 (0.47, 0.71) | -0.4 | 0.69 | 0.78 |
| RAGE | 517.3 | 0.140 | 1.12 (0.64, 0.71) | 0.4 | 0.70 | 0.80 |
| CXCL1 | 517.3 | 0.140 | 0.97 (0.82, 0.71) | -0.4 | 0.70 | 0.78 |
| IL2RB | 517.3 | 0.140 | 0.91 (0.56, 0.7) | -0.4 | 0.70 | 0.92 |
| GP6 | 517.3 | 0.140 | 0.95 (0.72, 0.71) | -0.4 | 0.70 | 0.91 |
| CD84 | 517.3 | 0.140 | 0.92 (0.59, 0.71) | -0.4 | 0.70 | 0.88 |
| SORT1 | 517.3 | 0.140 | 0.87 (0.41, 0.71) | -0.4 | 0.71 | 0.88 |
| BNP | 517.3 | 0.140 | 1.04 (0.84, 0.7) | 0.4 | 0.71 | 0.91 |
| SIRT2 | 517.3 | 0.140 | 0.97 (0.83, 0.71) | -0.4 | 0.73 | 0.95 |
| GRN | 517.3 | 0.140 | 1.12 (0.58, 0.71) | 0.3 | 0.73 | 0.91 |
| VEGFA | 517.3 | 0.140 | 1.11 (0.61, 0.71) | 0.3 | 0.73 | 0.92 |
| CCL11 | 517.3 | 0.140 | 1.09 (0.64, 0.71) | 0.3 | 0.74 | 0.95 |
| CITP (Log) | 517.4 | 0.140 | 0.93 (0.6, 0.7) | -0.3 | 0.75 | 0.98 |
| IL17D | 517.4 | 0.140 | 0.89 (0.43, 0.71) | -0.3 | 0.75 | 0.78 |
| TM | 517.4 | 0.140 | 1.11 (0.57, 0.71) | 0.3 | 0.76 | 0.88 |
| EGFR | 517.4 | 0.140 | 0.87 (0.34, 0.7) | -0.3 | 0.76 | 0.92 |
| LAPTGFbeta1 | 517.4 | 0.140 | 1.07 (0.67, 0.71) | 0.3 | 0.77 | 0.92 |
| ARTN | 517.4 | 0.140 | 1.09 (0.62, 0.7) | 0.3 | 0.77 | 0.95 |
| IL17RA | 517.4 | 0.140 | 1.06 (0.72, 0.7) | 0.3 | 0.77 | 0.91 |
| NEMO | 517.4 | 0.140 | 0.98 (0.82, 0.71) | -0.3 | 0.78 | 0.91 |
| CXCL16 | 517.4 | 0.140 | 1.11 (0.52, 0.71) | 0.3 | 0.78 | 0.91 |
| TNFRSF10A | 517.4 | 0.140 | 0.92 (0.48, 0.71) | -0.3 | 0.78 | 0.74 |
| IL17A | 517.4 | 0.140 | 0.96 (0.7, 0.71) | -0.3 | 0.79 | 0.92 |
| DLK1 | 517.4 | 0.140 | 0.95 (0.64, 0.71) | -0.3 | 0.79 | 0.91 |
| CCL3 | 517.4 | 0.140 | 0.96 (0.68, 0.7) | -0.3 | 0.79 | 0.88 |
| COL1A1 | 517.4 | 0.140 | 1.08 (0.6, 0.71) | 0.3 | 0.79 | 0.92 |
| CXCL9 | 517.4 | 0.140 | 0.96 (0.7, 0.7) | -0.3 | 0.80 | 0.92 |
| IFNGAMMA | 517.4 | 0.140 | 0.45 (0, 0.71) | -0.3 | 0.80 | 0.96 |
| TNFB | 517.4 | 0.140 | 0.94 (0.55, 0.71) | -0.2 | 0.80 | 0.98 |
| MCP2 | 517.4 | 0.140 | 1.04 (0.74, 0.71) | 0.2 | 0.81 | 0.97 |
| CXCL11 | 517.4 | 0.140 | 0.97 (0.77, 0.71) | -0.2 | 0.82 | 0.92 |
| LDLRECEPTOR | 517.4 | 0.140 | 1.06 (0.66, 0.7) | 0.2 | 0.82 | 0.91 |
| CD40 | 517.4 | 0.140 | 1.06 (0.64, 0.71) | 0.2 | 0.83 | 0.96 |
| PDL2 | 517.4 | 0.140 | 0.93 (0.49, 0.71) | -0.2 | 0.83 | 0.91 |
| CDH5 | 517.4 | 0.140 | 1.07 (0.58, 0.71) | 0.2 | 0.83 | 0.91 |
| ANG1 | 517.4 | 0.140 | 1.02 (0.85, 0.71) | 0.2 | 0.84 | 0.74 |
| CCL17 | 517.4 | 0.140 | 0.98 (0.79, 0.71) | -0.2 | 0.84 | 0.88 |
| MERTK | 517.4 | 0.140 | 0.94 (0.51, 0.71) | -0.2 | 0.84 | 0.88 |
| APN | 517.4 | 0.139 | 0.93 (0.46, 0.71) | -0.2 | 0.85 | 0.91 |
| CD4 | 517.4 | 0.139 | 1.08 (0.51, 0.71) | 0.2 | 0.85 | 0.91 |
| AXIN1 | 517.4 | 0.139 | 0.98 (0.84, 0.71) | -0.2 | 0.85 | 0.92 |
| TIE2 | 517.4 | 0.139 | 0.93 (0.42, 0.7) | -0.2 | 0.85 | 0.74 |
| DKK1 | 517.4 | 0.139 | 0.97 (0.71, 0.71) | -0.2 | 0.86 | 0.90 |
| FS | 517.4 | 0.139 | 0.95 (0.55, 0.7) | -0.2 | 0.87 | 0.86 |
| CD163 | 517.4 | 0.139 | 1.04 (0.65, 0.71) | 0.2 | 0.87 | 0.91 |
| PICP (Log) | 517.4 | 0.139 | 0.96 (0.56, 0.7) | -0.2 | 0.87 | 0.98 |
| MMP3 | 517.4 | 0.139 | 1.03 (0.69, 0.71) | 0.2 | 0.88 | 0.92 |
| FGF21 | 517.4 | 0.139 | 0.99 (0.82, 0.71) | -0.1 | 0.88 | 0.78 |
| TRANCE | 517.4 | 0.139 | 1.03 (0.72, 0.71) | 0.1 | 0.89 | 0.95 |
| NOTCH3 | 517.4 | 0.139 | 0.96 (0.52, 0.7) | -0.1 | 0.90 | 0.91 |
| CCL28 | 517.4 | 0.139 | 0.96 (0.51, 0.71) | -0.1 | 0.90 | 0.95 |
| CD244 | 517.4 | 0.139 | 1.03 (0.61, 0.71) | 0.1 | 0.90 | 0.92 |
| MMP12 | 517.4 | 0.139 | 1.02 (0.73, 0.71) | 0.1 | 0.91 | 0.91 |
| BMP6 | 517.4 | 0.139 | 0.98 (0.61, 0.71) | -0.1 | 0.92 | 0.74 |
| XCL1 | 517.4 | 0.139 | 1.02 (0.66, 0.71) | 0.1 | 0.92 | 0.88 |
| SPON1 | 517.4 | 0.139 | 1.03 (0.52, 0.71) | 0.1 | 0.92 | 0.91 |
| DECR1 | 517.4 | 0.139 | 0.99 (0.85, 0.71) | -0.1 | 0.93 | 0.88 |
| BLMHYDROLASE | 517.4 | 0.139 | 1.02 (0.62, 0.71) | 0.1 | 0.93 | 0.91 |
| GDF2 | 517.4 | 0.139 | 0.98 (0.55, 0.71) | -0.1 | 0.93 | 0.91 |
| CXCL6 | 517.4 | 0.139 | 0.99 (0.76, 0.71) | -0.1 | 0.94 | 0.95 |
| SELE | 517.5 | 0.139 | 1.01 (0.71, 0.71) | 0.1 | 0.94 | 0.91 |
| CD6 | 517.5 | 0.139 | 1.01 (0.66, 0.71) | 0.1 | 0.95 | 0.92 |
| IL17C | 517.5 | 0.139 | 1.01 (0.71, 0.71) | 0.1 | 0.95 | 0.92 |
| PDGFsubunitB | 517.5 | 0.139 | 1 (0.85, 0.71) | -0.1 | 0.96 | 0.74 |
| CDCP1 | 517.5 | 0.139 | 0.99 (0.68, 0.71) | -0.1 | 0.96 | 0.92 |
| IL8 | 517.5 | 0.139 | 0.99 (0.69, 0.71) | 0.0 | 0.96 | 0.92 |
| IgGFcreceptorIlb | 517.5 | 0.139 | 1.01 (0.76, 0.71) | 0.0 | 0.97 | 0.88 |
| PON3 | 517.5 | 0.139 | 0.99 (0.66, 0.71) | 0.0 | 0.97 | 0.92 |
| HAOX1 | 517.5 | 0.139 | 1 (0.83, 0.7) | 0.0 | 0.98 | 0.91 |
| IL2 | 516.9 | 0.141 | 8.94E+25 (0, 0.79) | 0.0 | 0.98 | 0.92 |
| BP14E | 517.5 | 0.139 | 1 (0.77, 0.71) | 0.0 | 1.00 | 0.95 |

Supplemental table 10 – Pearsons’ correlation coefficient between proteins included in the proteomic profile.

|  | **MMP7** | **MMP1** | **CD8A** | **GAL4** | **PAI** | **PGLYRP1** | **GRP** | **CST5** | **NT3** | **TRANCE** | **PAI** | **NTproBNP (Log)** | **IL5** |
| --- | --- | --- | --- | --- | --- | --- | --- | --- | --- | --- | --- | --- | --- |
| **MMP7** | 1.00 |  |  |  |  |  |  |  |  |  |  |  |  |
| **MMP1** | 0.05 | 1.00 |  |  |  |  |  |  |  |  |  |  |  |
| **CD8A** | 0.07 | 0.08 | 1.00 |  |  |  |  |  |  |  |  |  |  |
| **GAL4** | 0.23 | 0.21 | 0.11 | 1.00 |  |  |  |  |  |  |  |  |  |
| **PAI** | 0.17 | 0.49 | 0.09 | 0.28 | 1.00 |  |  |  |  |  |  |  |  |
| **PGLYRP1** | -0.02 | 0.14 | 0.23 | 0.17 | 0.16 | 1.00 |  |  |  |  |  |  |  |
| **GRN** | 0.26 | 0.15 | 0.22 | 0.36 | 0.24 | 0.22 | 1.00 |  |  |  |  |  |  |
| **CST5** | 0.15 | 0.19 | 0.21 | 0.20 | 0.05 | 0.11 | 0.18 | 1.00 |  |  |  |  |  |
| **NT3** | 0.11 | 0.04 | 0.09 | 0.07 | 0.16 | -0.03 | 0.15 | 0.14 | 1.00 |  |  |  |  |
| **TRANCE** | -0.01 | 0.09 | 0.18 | -0.05 | 0.08 | 0.09 | 0.16 | 0.03 | 0.09 | 1.00 |  |  |  |
| **PSPD** | 0.18 | 0.01 | 0.03 | 0.19 | 0.08 | 0.01 | 0.20 | 0.07 | 0.04 | 0.04 | 1.00 |  |  |
| **NTproBNP (Log)** | -0.02 | 0.06 | 0.13 | 0.02 | -0.04 | 0.15 | 0.05 | 0.14 | -0.03 | 0.01 | 0.01 | 1.00 |  |
| **IL5** | -0.04 | 0.05 | -0.03 | 0.03 | 0.02 | 0.02 | -0.02 | 0.04 | -0.07 | -0.02 | 0.01 | 0.02 | 1.00 |
